# Whole RNA-Seq Analysis Reveals Longitudinal Proteostasis Network Responses to Photoreceptor Outer Segment Trafficking and Degradation in RPE Cells

**DOI:** 10.3390/cells14151166

**Published:** 2025-07-29

**Authors:** Rebecca D. Miller, Isaac Mondon, Charles Ellis, Anna-Marie Muir, Stephanie Turner, Eloise Keeling, Htoo A. Wai, David S. Chatelet, David A. Johnson, David A. Tumbarello, Andrew J. Lotery, Diana Baralle, J. Arjuna Ratnayaka

**Affiliations:** 1 School of Clinical and Experimental Sciences (CES), Faculty of Medicine, University of Southampton, MP 806, Tremona Road, Southampton SO16 6YD, UK; r.d.miller@soton.ac.uk (R.D.M.); isaacmondon@gmail.com (I.M.); c.e.ellis@soton.ac.uk (C.E.); a.m.muir@soton.ac.uk (A.-M.M.); stephanie.turner@soton.ac.uk (S.T.); e.e.keeling@soton.ac.uk (E.K.); a.j.lotery@soton.ac.uk (A.J.L.); 2 School of Human Development and Health, Institute for Developmental Sciences Building, Tremona Road, Southampton, Hampshire SO16 6YD, UK; h.a.wai@soton.ac.uk (H.A.W.); d.baralle@soton.ac.uk (D.B.); 3 Biomedical Imaging Unit, University of Southampton, MP12, Tremona Road, Southampton SO16 6YD, UK; d.s.chatelet@soton.ac.uk (D.S.C.); d.a.johnston@soton.ac.uk (D.A.J.); 4 School of Biological Sciences, Faculty of Environmental and Life Sciences, Life Sciences Building 85, University of Southampton, Highfield Campus, Southampton SO17 1BJ, UK; d.a.tumbarello@soton.ac.uk; 5 Eye Unit, University Hospital Southampton NHS Foundation Trust, Southampton SO16 6YD, UK; 6 NIHR Southampton Biomedical Research Centre, Southampton Centre for Biomedical Research, Tremona Road, Southampton, Hampshire SO16 6YD, UK

**Keywords:** retinal pigment epithelium (RPE), RNA-seq, photoreceptor outer segments (POS), trafficking, proteostasis, age-related macular degeneration (AMD), inherited retinal dystrophies

## Abstract

RNA-seq analysis of the highly differentiated human retinal pigment epithelial (RPE) cell-line ARPE-19, cultured on transwells for ≥4 months, yielded 44,909 genes showing 83.35% alignment with the human reference genome. These included mRNA transcripts of RPE-specific genes and those involved in retinopathies. Monolayers were fed photoreceptor outer segments (POS), designed to be synchronously internalised, mimicking homeostatic RPE activity. Cells were subsequently fixed at 4, 6, 24 and 48 h when POS were previously shown to maximally co-localise with Rab5, Rab7, LAMP/lysosomes and LC3b/autophagic compartments. A comprehensive analysis of differentially expressed genes involved in proteolysis revealed a pattern of gene orchestration consistent with POS breakdown in the autophagy-lysosomal pathway. At 4 h, these included elevated upstream signalling events promoting early stages of cargo transport and endosome maturation compared to RPE without POS exposure. This transcriptional landscape altered from 6 h, transitioning to promoting cargo degradation in autolysosomes by 24–48 h. Longitudinal scrutiny of mRNA transcripts revealed nuanced differences even within linked gene networks. POS exposure also initiated transcriptional upregulation in ubiquitin proteasome and chaperone-mediated systems within 4–6 h, providing evidence of cross-talk with other proteolytic processes. These findings show detailed evidence of transcriptome-level responses to cargo trafficking and processing in RPE cells.

## 1. Introduction

Damage to the retinal pigment epithelium (RPE) plays a pivotal role in the onset and progression of irreversible blinding diseases such as age-related macular degeneration (AMD) and inherited retinal diseases (IRDs). AMD is the commonest cause of blindness amongst adults in the UK. A study published over a decade ago estimated ~150 million individuals to be affected globally with some form of AMD, with a further 10 million people suffering from the more advanced/sight-threatening stages of the condition [1]. RPE dysfunction is also linked with several IRDs such as Stargardt and Best disease that is present from childhood to conditions such as Sorsby fundus dystrophy which manifests later in life [2,3,4]. Although these retinopathies can be managed in some patients, none have any effective treatments to date. Consequently, there is considerable interest in unravelling the various disease-linked mechanisms through which RPE cells eventually become dysfunctional and atrophic. 

The RPE monolayer consists of neuroepithelial-derived post-mitotic cells and is located beneath the neuroretina where the RPE intimately associate with overlying photoreceptors. We and others have shown that each RPE cell is responsible for maintaining > 200 photoreceptors in the mammalian retina [5,6]. Hence, damage and eventual death of RPE cells can have disproportionate consequences for photoreceptor survival. RPE atrophy is therefore an important early pathological feature of AMD and some IRDs, which is typically followed by irreversible photoreceptor loss and visual impairment. RPE cells internalise and degrade shed outer segments from overlying photoreceptors as part of the daily photoreceptor renewal cycle [7,8]. Each RPE cell is therefore subject to a considerable proteolytic burden, making the RPE monolayer one of the most proteolytically challenged tissues in the human body. Following the receptor-mediated binding of photoreceptor outer segments (POS) on the apical RPE surface, POS cargos are internalised and trafficked via the phagosome and autophagy–lysosomal pathway for degradation. With advanced age, the inefficient breakdown of lipid-rich POS cargos results in the accumulation of partially degraded and modified POS membranes, which together with various bisretinoid molecules, accumulate within RPE cells as lipofuscin and its derivatives [8,9]. These intracellular aggregates were demonstrated to mediate a range of cytotoxic effects [7,9] and possess inherent autofluorescent properties that can be non-invasively visualised in patients as fundus autofluorescence [10,11]. Loss of RPE cells therefore appears as a region devoid of autofluorescence and is a clinical end-point for assessing retinopathies and patterns of further retinal atrophy. 

Given the relevance of these pathogenic molecules in a well-established pathway leading to RPE atrophy, the trafficking and proteolytic breakdown of POS and related cargos are of great interest, as insights into these mechanisms could provide clinically relevant data that may be beneficial in devising effective new treatments. In our previous studies, we delineated the timeline of POS trafficking and processing in distinct vesicular compartments of the phagosome and autophagy–lysosomal pathway [12]. Furthermore, we showed how disease-linked mechanisms such as oxidative stress, impaired autophagy, exposure to amyloid beta (Ab) and oxidative modification of POS cargos, which occur in the ageing retina, impair POS processing, contributing to RPE atrophy [12,13,14]. The dynamic intracellular trafficking events associated with these processes, which we reported via confocal and ultrastructural imaging as well as cellular responses to cargos within RPE lysosomes that were quantified biochemically, led to an *a priori* hypothesis that POS processing may also elicit a cascade of events at the mRNA level. However, to our knowledge, this has yet to be systematically studied. Herein, we set out to assess transcriptional responses to POS trafficking and degradation using an *in vitro* cell model of the RPE. 

## 2. Materials and Methods

### 2.1. Culture of RPE Cells

The human RPE cell line (ARPE-19) [15] was obtained from the American Tissue Culture Collection (ATCC, Manassas, VA, USA) and cultured in a 37 °C humidified incubator with 5% CO_2_ and 95% air. Low-passage (p 2–5) cells were maintained in T25 cm^2^ flasks in 5 mL of media composed of Dulbecco’s modified Eagle’s Medium (DMEM) (Life Technologies, Warrington, UK) supplemented with 4.5 g/L L-D glucose, L-glutamine and pyruvate. Media was supplemented with 1% heat-inactivated foetal bovine serum (Sigma Aldrich, Pool, UK) and 1% penicillin–streptomycin stock solution (10,000 units/mL penicillin, 10 mg/mL streptomycin in 0.85% saline) (Sigma Aldrich, Pool, UK). Transwell membranes of 12 mm diameter inserts with 0.4 mm pore PET polyester membranes (Sigma Aldrich, Pool, UK) were pre-coated with 50 mg/mL fibronectin (Sigma Aldrich, Pool, UK). Cells in low passages (*p* < 5) were seeded at a density of 1.25 × 10^4^/well and cultured for up to 4 months prior to experiments [16]. RPE monolayers in these transwell inserts were maintained with media volumes of 0.5 mL and 2.0 mL in the apical and basal compartments, respectively. Every 3–4 days, a complete media change was performed in the apical compartment, whilst a 20% media change was carried out in the basal chamber. 

### 2.2. Isolation and Quantification of Photoreceptor Outer Segments (POS)

Fresh porcine eyes obtained from a local butcher were isolated immediately upon receipt as described before [17] and pooled in KCl buffer with sucrose (buffer containing 0.3 M KCl, 10 mM HEPES, 0.5 mM CaCl_2_, 1 mM MgCl_2_ and 48% sucrose). Retinas were homogenised by gently shaking for 2 min, then divided into 1.5 mL tubes. The solutions were centrifuged for 5 min at 5000× *g*. The pellet was discarded, and the supernatant (750 mL) passed through sterile gauze into fresh 1.5 mL tube containing 750 mL KCl buffer (buffer containing 0.3 M KCl, 10 mM HEPES, 0.5 mM CaCl_2_ and 1 mM MgCl_2_). This was centrifuged for 7 min at 4000× *g*, after which the supernatant was removed and the pellet washed with 1× PBS before being centrifuged for a further 7 min at 4000× *g*. This step was repeated twice. The concentration of the prepared POS aliquots was quantified using the Pierce BCA Protein Assay (ThermoFisher Scientific, Loughborough, UK). The POS protein content was compared to BSA albumin protein standards between 20 and 2000 mg/mL by recording the absorbance at 562 nm using a plate reader (BMG LabTech, Ortenberg, Germany). Isolated POS molecules were tagged with the fluorescent dye fluorescein isothiocyanate (FITC). A stock solution was prepared using 10 mg FITC (Life Technologies, Warrington, UK) in 4.5 mL Na_2_CO_3_ buffer (pH 9.5). A 5 mL washing solution was prepared with 0.2 M Sorenson’s phosphate buffer containing 10% sucrose, 20 mM phosphate buffer and 5 mM Taurine. Isolated POS pellets were subsequently resuspended in 5 mL of the wash buffer. A total of 1.5 mL of the FITC stock solution was added and incubated for one hour, protected from light, on a rotating plate. Following this, the POS-FITC solution was transferred to 1.5 mL Eppendorf tubes and centrifuged at 3000× *g* for 5 min. The resulting pellets were resuspended in PBS in small aliquots and stored at −80 °C.

### 2.3. Synchronised POS Feeding Assay

RPE monolayers on transwell membranes were incubated at 17 °C for 30 min, after which 4 mg/cm^2^ of POS-FITC were added to each well at the recommended POS feeding concentration described previously [18]. In order to ensure the synchronised internalisation of isolated POS molecules, the cultures were returned to the incubator for a further 30 min at 17 °C, which ensured maximal binding prior to internalisation [19]. The media was removed to eliminate the presence of any unbound POS-FITC molecules and replaced with fresh pre-warmed media. The cultures were promptly returned to a 37 °C humidified incubator to commence the synchronised internalisation of POS cargos by RPE cells. Following this procedure, transwell membranes were fixed in 4% paraformaldehyde (PFA) solution at 4, 6, 24 or 48 h.

### 2.4. RNA Extraction and Quantification

Total RNA was extracted from RPE monolayers at each aforementioned timepoint. Cells were scraped off transwell membranes and pelleted using the RNA*later*™ Stabilisation Solution (ThermoFisher Scientific, Loughborough, UK) for storage at −20 °C. RNA was extracted using the RNAeasy^®^ Mini Kit (Qiagen, Hilden, Germany) following the manufacturers’ instructions. Briefly, the samples stored in RNA*later* were allowed to thaw and subsequently pelleted. The RNA*later* solution was carefully removed and 350 mL of RLT buffer was added to homogenise the cells. An equal amount of 70% ethanol was then added to the tube prior to mixing, after which 700 mL of the resulting solution, including any precipitate, was transferred to an RNeasy Mini spin column and centrifuged for 15 s at ≥8000× *g*. Following this, 700 µL of the RW1 buffer was added to the RNeasy spin column and centrifuged for 15 s at 8200× *g*. This procedure was repeated using 500 mL of RPE buffer before a final centrifugation step at 16,000× *g* for 1 min to dry the membrane. The RNeasy spin column was centrifuged for 1 min after the subsequent addition of 30 mL RNase-free water to elute the RNA. The concentration of RNA was quantified using a NanoDrop spectrophotometer (ThermoFisher Scientific Loughborough, UK) as well as a Qubit™ RNA HS Assay Kit (Invitrogen, Paisley, UK).

### 2.5. RNA-Seq

RNA samples were commercially sequenced (Novogene, Cambridge, UK) using the Illumina non-stranded mRNA (poly A enrichment) library preparation method (Novaseq 6000 Illumina PE150, Illumina, San Diego, CA, USA) to produce paired reads with a read depth of 40 million. This produced 64 raw fastq files for subsequent analysis. 

### 2.6. Post Sequencing Analysis of Whole mRNA

A total of 64 raw fastq files (32 RNA samples, paired-end, forward and reverse reads) from eight sample groups with four biological repeats (sample groups were from each RNA extraction timepoint—4 h, 6 h, 24 h and 48 h—in either feed group: POS naïve/control or POS-exposed) were used for downstream identification of differentially expressed genes (DEGs). A quality assessment step was initially undertaken using fastp and MultiQC [20,21]. Fastp was used for sample quality control, including adapter trimming, low-quality read filtering (Q < 20), and removal of short reads (length < 30 bp) [21]. MultiQC was then used to aggregate the results from all 64 fastp reports into a single comprehensive summary [20]. Trimmed reads were quantified against the human reference transcriptome GRCh38.p14 from RefSeq using the Salmon tool [22]. Transcript-level quantifications were then imported into 3D RNA-seq [23] using the Tximport R package [24], applying the lengthScaledTPM method to generate gene-level read counts for downstream analysis. Low-expressed genes were removed using the R function RNAdeNoise which defines a specific, unique cutoff threshold to each sample [25]. 

The count matrices of expressed genes were analysed for DEGs using the R programme DESeq2 [26]. DESeq2 was used to perform differential expression analysis on the eight sample groups, each consisting of four biological repeats, to assess differences in expression between the treatment and control groups across different timepoints. In this analysis, there were ten contrast groups formed from four feed comparisons (control vs. POS) at each of the four RNA extraction timepoints (4 h, 6 h, 24 h and 48 h), and six total time comparisons (4 h vs. 6 h, 4 h vs. 24 h and 4 h vs. 48 h) within each of the two feed groups (POS naïve/control or POS-exposed). Genes with an adjusted *p*-value < 0.01 were considered significantly differentially expressed. DESeq2 uses the median of ratios method to normalise the count matrix prior to performing pairwise comparisons between each contrast group to produce a log fold change, *p* value and adjusted *p* value between the expression level for each gene. The median of ratios method accounts for sequencing depth and RNA composition by calculating size factors for each sample and then dividing the raw counts by these size factors (https://hbctraining.github.io/DGE_workshop/lessons/02_DGE_count_normalization.html, accessed on 1 April 2025). 

### 2.7. Pathway Formation Using Cytoscape and Analysis Using DAVID

Each of the three degradative pathways in the cellular proteostasis system was identified using open-access resources of the Proteostasis Consortium (https://www.proteostasisconsortium.com/pn-annotation/, accession on 21 February 2024): (1) the autophagy–lysosomal pathway, (2) the ubiquitin proteosome system, and (3) the chaperone-mediated pathway, which were visualised using the Cytoscape programme [27]. Gene interaction diagrams were developed in-house based on knowledge of well-characterised gene networks and online resources which describe the autophagy-lysosomal pathway (Cell Signaling Technology: https://www.cellsignal.com/pathways/autophagy-signaling-pathway, accessed on 10 August 2024; CUSABIO: https://www.cusabio.cn/pathway/Autophagy.html, accessed on 3 August 2024). Gene networks of the ubiquitin proteosome system and the chaperone-mediated systems were grouped, mapping to specific parts of each pathway. The ubiquitin proteosome system was split into six groups—E1, E2, E3 HECT, E3 RING and DUBs—as well as the proteosome [28,29], and was also developed using an online resource (Ubiquigent: https://www.ubiquigent.com/science, accessed on 14 September 2024). The chaperone-mediated pathway was grouped into four regions: the hsp70 cycle, the hsp90 cycle, the direct manner and ribosomal chaperones [30,31,32]. DEGs identified for each timepoint in POS exposed RPE were input into the UniProt ID mapping tool (https://www.uniprot.org/id-mapping, accessed on 1 April 2025), followed by the identification of gene ontology networks mapping to their respective molecular functions using the Database for Annotation, Visualization, and Integrated Discovery (DAVID) bioinformatics tool (https://davidbioinformatics.nih.gov/, accessed on 1 April 2025) [33]. 

### 2.8. Statistical Analyses

RNA-seq experiments were performed with four biological replicates per condition. Statistical comparisons of genes involved in lysosome and autophagy regulation were carried out using a Wald test following a negative binomial generalized linear model, where significant differences are indicated as *p* ≤ 0.05 (*), *p* ≤ 0.001 (***) and *p* ≤ 0.0001 (****). 

## 3. Results

### 3.1. Whole RNA-Seq Analyses of Highly Differentiated Monolayers of the Human RPE Cell Line (ARPE-19) Showing the Expression of RPE-Specific Genes and Genes Linked with Retinopathies

**Figure 1:**
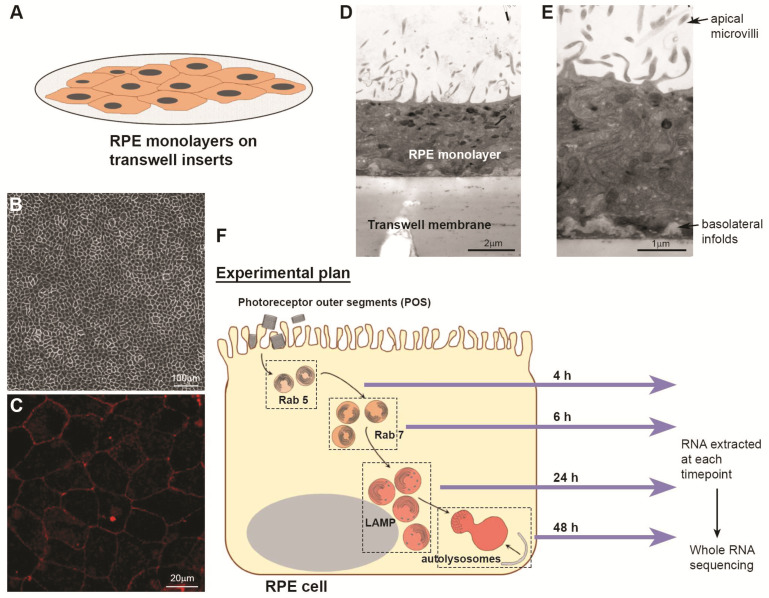
Overview of an experimental plan to identify gene networks involved in photoreceptor outer segment (POS) trafficking by RPE cells. (**A**) Schematic diagram illustrating the formation of a confluent retinal pigment epithelial (RPE) monolayer on transwell membranes. (**B**) Following maturation that includes rapid pigmentation over a 2–4 month period, RPE monolayers display a characteristic ‘cobblestone-like’ morphology. (**C**) Representative image from confocal immunofluorescence studies showing the formation of zonula occludens-1 (ZO-1) tight-junction proteins between RPE cells. (**D**,**E**) Representative electron micrographs showing cross-sections of mature RPE monolayers cultured on transwell membranes for long periods (≥4 months) that structurally recapitulate features of *in-situ*/native RPE, including the presence of apical microvilli, convoluted basolateral infolds, and apically distributed electron-dense pigments along an apical–basal axis. Scale bars in (**B**,**C**) correspond to 100 μm and 20 μm whilst those in (**D**,**E**) correspond to 2 μm and 1 μm, respectively. (**F**) RPE monolayers cultured on transwell membranes for ≥4 months were synchronously pulsed with isolated POS. Whole RNA was extracted at timepoints where previous studies identified POS cargos to be maximally co-localised with Rab5- (4 h) and Rab7- (6 h) positive compartments, LAMP-labelled lysosomes (24 h) and LC3b autophagy membranes (48 h). Whole RNA sequencing was carried out to obtain an unbiased map of gene transcripts at each timepoint for downstream scrutiny using bioinformatics tools.

We and others have previously shown that the human RPE cell line ARPE-19, when seeded at high densities on transwell membranes, develops a confluent monolayer which recapitulates the physiology and ultrastructure of the native/*in situ* RPE monolayer in the retina [16,34]. Such highly differentiated cultures, which typically take approximately 4 months to develop, can then be used as a versatile platform to obtain fundamental insights into retinopathies involving the RPE, and to screen novel compounds for drug discovery studies. Accordingly, ARPE-19 cells seeded at a density of 1.25 × 10^4^/well on 0.4 mM pore size, clear transwell PET membranes (Figure 1A) and cultured for 4 months in high-glucose DMEM with pyruvate and minimal foetal calf serum [35], rapidly become confluent via contact inhibition, which minimises the likelihood of de-differentiation and/or transition to an epithelial–mesenchymal phenotype. 

Such cultures develop a characteristic ‘cobblestone-like’ RPE morphology with well-defined cell borders, producing *de novo*-synthesised melanin granules that appear as dark pigmentation to the naked eye (Figure 1B). Studies using confocal immunofluorescence microscopy show the presence of zonula occludens-1 (ZO-1) tight junctions between RPE cells which are part of the junctional complex (Figure 1C). Further scrutiny by electron microscopy in cross-sectional micrographs reveals a highly specialised monolayer with numerous apical microvilli and invaginations of the basolateral membrane (Figure 1D,E), where we and others previously reported the appearance of sub-RPE deposits [34,36,37,38]. Electron-dense pigments, including the aforementioned melanin granules are also observed preferentially distributed to the apical surface, similarly to native RPE. These highly differentiated monolayers of ARPE-19 cells can be stably maintained for long periods, even exceeding 12 months [16,34,39], further adding to their utility and value. 

To determine whether this *in-vitro* RPE monolayer also reproduced a faithful mRNA profile of its *in-situ*/native counterpart, we carried out an unbiased whole transcriptomic (RNA-seq) analysis to a read depth of 40 million reads. Data were obtained from four biologically independent replicates that were aligned with the experiments described below, where monolayers were synchronously pulsed with isolated POS and fixed at key timepoints for RNA extraction. Parallel cultures which remained POS-naïve (RPE without any exposure to POS) acted as controls. Data were subsequently quantified against the human reference genome (GRCh38.p14) using the Salmon tool which produced a total of 44,909 genes (83.35% alignment of reads with the transcriptome). Before proceeding further, we assessed whether genes that are specifically associated with the identity of human RPE cells were expressed in our cultures. Read counts were obtained for all tested genes which included *BEST1*, *OCA2*, *CLDN3*, *LRAT*, *OTX2*, *PAX6*, *RDH5*, *RDH10*, *RLBP1*, *RPE65*, *SERPINF1*, *SOX9*, *TJP1*, *TRPM1* and *TYR* (Supplementary Table S1). We also tested for genes associated with AMD from an open-access genome-wide association study catalogue (https://www.ebi.ac.uk/gwas/, accessed on 31 July 2024), which identified 174 genes from a total of 213 reported genes (81.7% of the listed AMD genes) in our dataset (Supplementary Table S2). As genetic mutations/variants expressed in RPE cells are known to play an important role in several IRDs, we also assessed these in our study. Cultured ARPE-19 cells expressed 18 of the 25 reported genes (72% of the listed RPE-specific genes involved in inherited retinopathies) (Supplementary Table S3). Finally, as mature/healthy RPE cells form a highly specialised tissue, we also tested for any evidence of significant changes to cell cycle genes, where the fewest changes can be anticipated in stable monolayers. Transcripts for all 124 cell cycle-related genes from an open-access database (https://www.genome.jp/kegg/pathway.html, accessed on 31 July 2024) were expressed in cultured RPE (Supplementary Table S4). However, no DEGs were reported when these were assessed for any significant changes (adjusted *p* ≤ 0.01) between control vs. RPE cultures fed POS. Collectively, our findings show that confluent ARPE-19 monolayers on transwell membranes are close transcriptomic approximates of its native/*in-situ* counterpart, complementing its physiological and structural recapitulation under *in-vitro* conditions. 

### 3.2. Whole RNA-Seq Analyses of Genes in the RPE Proteostasis Network

**Figure 2:**
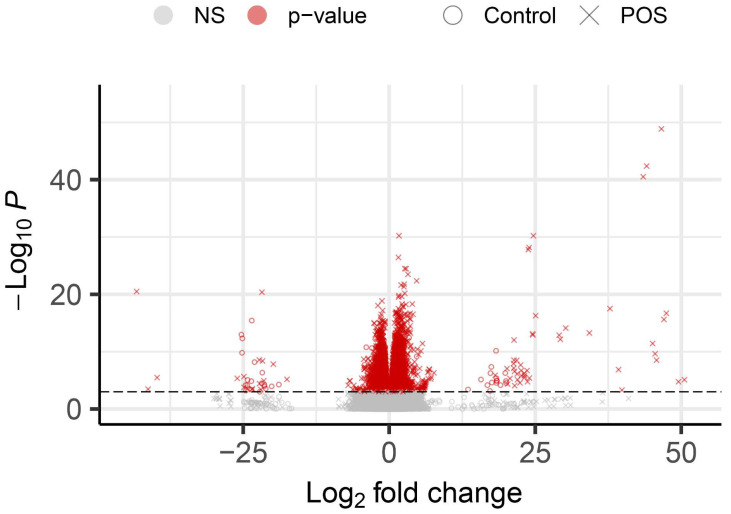
Volcano plot showing changes for all genes as a result of exposure to photoreceptor outer segments (POS). Graph displays all 44,909 genes of the dataset in six separate timepoint comparisons (Control 4 h vs. Control 6 h, Control 4 h vs. Control 24 h, Control 4 h vs. Control 48 h, POS 4 h vs. POS 6 h, POS 4 h vs. POS 24 h, and POS 4 h vs. POS 48 h) for a total of 269,454 variables. These were plotted by their log2-fold change against −log10 *p* value. Differentially expressed genes (DEGs) are indicated in red and have an adjusted *p* value < 0.01. Variables marked by X show timepoint comparisons within POS-fed conditions, whilst those denoted by O are from POS-naïve/control conditions. In total, 13,881 significant variables were reported with 6256 unique DEGs. The control condition produced 4811 significant variables with 3475 unique DEGs, whereas POS exposure resulted in 9070 significant variables with 5554 unique DEGs.

The RPE monolayer internalises shed outer segments from overlying photoreceptors as part of the daily photoreceptor renewal cycle. Following receptor-mediated internalisation, POS cargos are trafficked in the phagosome/endosome and autophagy–lysosomal pathway for subsequent degradation. Our previous work delineated the timescales at which intracellular compartments of this trafficking network maximally co-localised with POS cargos. Based on those findings we hypothesised that the trafficking and proteolytic processing of POS cargos may also be correlated with mRNA-level changes in the cells’ proteostasis network. A key objective of this study, therefore, was to unravel this relationship and gain further insights into this important homeostatic function of RPE cells. To achieve this aim, cultured ARPE-19 monolayers were fixed at 4, 6, 24 and 48 h timepoints following initiation of the POS pulse-chase assay (Figure 1F). Initially, in order to assess the global variability between the read counts of samples, a principal component analysis (PCA) was carried out. This showed a broad pattern of segregation between 4 and 6 h vs. 24 and 48 h for all genes in the dataset (Supplementary Figure S1). Fewer significant differences between genes were observed when comparing control vs. POS exposure at a single timepoint. By contrast, a greater number of DEGs were observed when comparing read counts across the experimental time course, where more DEGs were observed as a result of POS exposure (Figure 2). This suggested that substantially more genes were either significantly up- or downregulated as a consequence of POS exposure compared to any baseline (homeostatic) mRNA-level changes occurring in POS-naïve RPE cells over time.

Elements contributing to the cellular proteostasis system involves several hundred genes which we identified using open access resources of the Proteostasis Consortium (https://www.proteostasisconsortium.com/pn-annotation/, accessed on 21 February 2024). This consisted of a total of 2905 genes covering several pathways. Recent studies also provide evidence of cross-talk between these well-defined degradative processes. We identified three pathways that covered a broad spectrum of the known proteostasis network: (1) the autophagy–lysosomal pathway, (2) the ubiquitin proteosome system and (3) the chaperone-mediated pathway. A breakdown of genes expressed by RPE cells in our study showed 848 genes in the autophagy–lysosomal pathway (Supplementary Table S5), 1185 genes in the ubiquitin proteosome system (Supplementary Table S6) and 1145 genes in the chaperone-mediated pathway (Supplementary Table S7). As expected, some genes were shared between these three pathways. A total of 92 genes were shared between the autophagy–lysosomal and chaperone-mediated pathways, while 110 genes overlapped between the autophagy–lysosomal and the ubiquitin proteosome system. A further 95 genes were shared between the chaperone-mediated pathway and the ubiquitin proteosome system. All three pathways were found to share 24 genes. Furthermore, we explored if genes in our samples that were identified in the proteostasis network were also shared with genes reported to be linked with AMD. Of the 213 AMD-associated genes in our samples, 13 were involved in the proteostasis network (*MARK4*, *ATP6V0D1*, *DAPK3*, *SNX7*, *BAG6*, *HERPUD1*, *NPLOC4*, *TOMM40*, *RPL3*, *HERC2*, *BARD1*, *BRAP* and *DPP9*). Of the 25 RPE-specific IRD-associated genes, 18 were mapped in our alignment, of which 3 were shared with those in the proteostasis network, specifically in the chaperone-mediated pathway (*AIPL1*, *BBS10*, *BBS12*). 

### 3.3. Whole RNA Sequencing Reveals Transcriptomic Changes in the Proteostasis Network in Response to Photoreceptor Outer Segment Trafficking by RPE Cells

**Figure 3:**
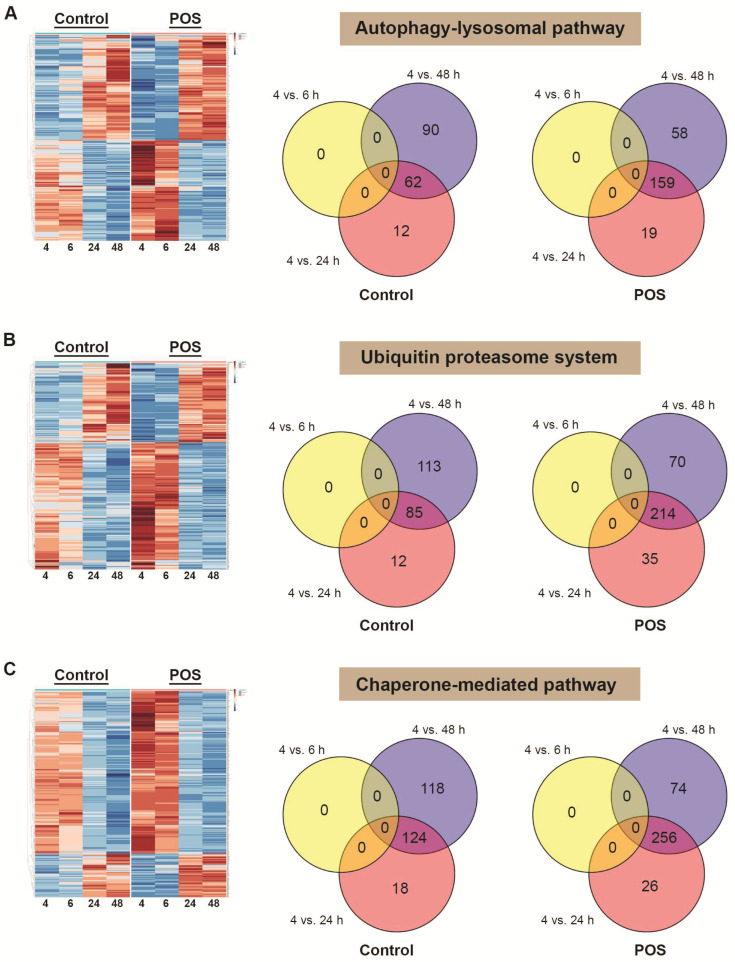
Analyses of genes in the proteostasis network as a function of photoreceptor outer segment (POS) trafficking by RPE cells. (**A**) Differentially expressed genes (DEGs) present in the autophagy–lysosomal pathway correlated with POS processing at 4, 6, 24 and 48 h following pulse-chase. Heatmaps show normalised count data from DEGs from control RPE cultures and those synchronously fed with POS, which were combined to enable a direct comparison of z scores between the two conditions. Corresponding Venn diagrams of DEGs in the autophagy–lysosomal pathway where count data was normalised to produce *p* values, adjusted *p* values and log2 fold changes between each timepoint (6, 24 and 48 h) compared to the initial 4 h timepoint. These show the number of DEGs that have an adjusted *p* < 0.01 at each timepoint. Genes were retained if the threshold was met in at least one timepoint comparison in either the control or POS-fed RPE, which reduced the 845 genes in the autophagy–lysosomal pathway to 272 DEGs. (**B**) A similar scrutiny of genes in the ubiquitin proteasome system was carried out, which revealed 363 DEGs distributed in the Venn diagram from an initial list of 1179 genes. (**C**) Parallel analyses of genes in the chaperone-mediated pathway identified 396 DEGs from an initial list of 1141 genes. Note: some genes overlapped with several pathways. This approach enabled a detailed scrutiny of gene networks in the proteostasis network in response to POS trafficking and processing by RPE at timepoints where these cargos were maximally co-localised with early and mature phagosomes/endosomes, lysosomes, and autophagy bodies.

Next, we sought to test our hypothesis and determine whether POS trafficking and degradation were correlated with significant mRNA level changes in the cells’ proteolytic system. We primarily focused on the autophagy–lysosomal pathway which degrades internalised POS molecules [7]. Here, RPE monolayers were synchronously pulsed with POS and fixed at 4, 6, 24 and 48 h timepoints at which our previous work showed these cargos to be maximally co-localised with early phagosomes/endosomes (Rab 5), mature phagosomes/endosomes (Rab 7), lysosomes (LAMP1 and LAMP2) and autolysosomes (LC3b), respectively. In parallel, sister cultures naïve for POS (controls) were fixed for whole RNA extraction at identical timepoints. DEGs were identified using DESeq2 software. An initial screening of constituent genes that were differentially expressed at an adjusted *p* < 0.01 value between control vs. POS-fed RPE within each timepoint failed to identify any targets. Lowering this threshold to an adjusted value of *p* < 0.05 identified only five genes (*PLD1*, *RILP*, *IGF1*, *MAP2K1* and *STBD1*). Reducing the adjusted threshold further to *p* < 0.1 identified several additional genes (*ABHD5*, *IGF1*, *MAP2K1*, *PLD1*, *PPARGC1A*, *RILP*, *STBD1*) alongside those identified by a non-adjusted threshold of *p* < 0.1 in a step-wise manner in the autophagy–lysosomal pathway that were altered due to POS exposure (Supplementary Table S8). However, targets identified through diminishing statistical thresholds in this manner are likely to produce genes of increasingly limited biological relevance. Relaxation of this threshold was also useful in this exploratory research to cast a wider net and to identify potential genes of interest that were not discovered using a stricter threshold. The requirement to lower the threshold to discover genes of interest in the feed comparison also made it abundantly clear from the outset that more changes were occurring in timepoint comparisons which allowed for more focused studies in this area. Hence, in order to identify DEGs at the higher significance threshold of adjusted *p* < 0.01, we compared read counts between different timepoints. Normalised count data from DEGs in control RPE cultures and those fed with POS were combined to enable a direct comparison of z scores between the two conditions. Our findings are shown as heatmaps for genes in the autophagy–lysosomal pathway (Figure 3A), the ubiquitin proteosome system (Figure 3B) and the chaperone-mediated pathway (Figure 3C). For each pathway, corresponding Venn diagrams show the number of DEGs that have an adjusted *p* < 0.01 at each timepoint, where count data were normalised to produce *p* values, adjusted *p* values and log2 fold changes between each timepoint (6, 24 and 48 h), compared to the initial 4 h timepoint. These reveal the temporal distribution of DEGs for control vs. POS-fed conditions across the three proteolytic pathways as well as the extent of their overlap. 

**Figure 4:**
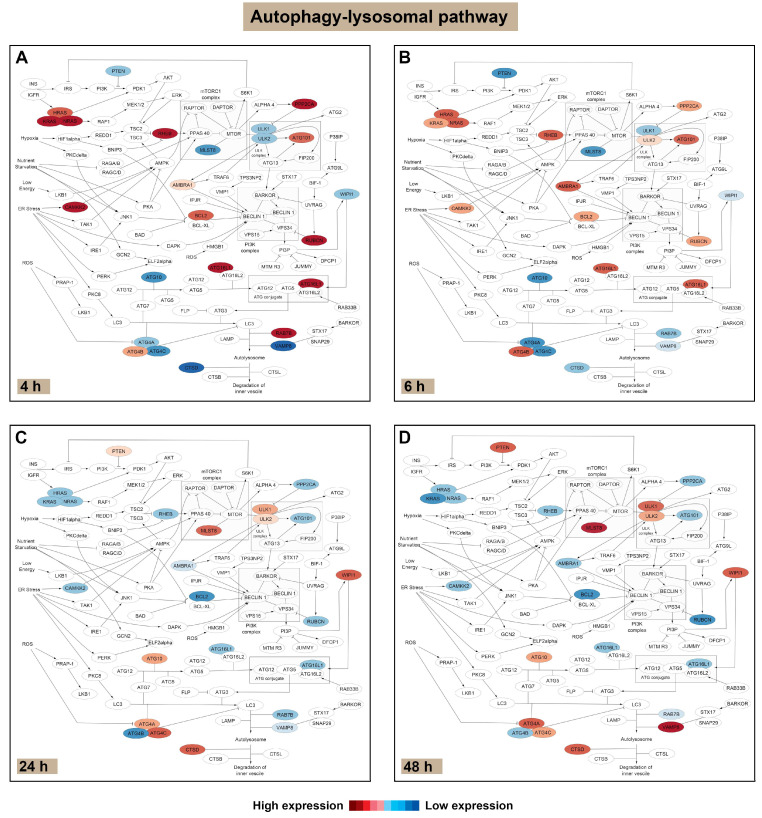
Visualisation of genes in the autophagy–lysosomal pathway of RPE cells following exposure to photoreceptor outer segments (POS). Cytoscape was used to map the autophagy–lysosomal network, where differentially expressed genes (DEGs) are colour-coded to highlight their relative extent of upregulation (red) or downregulation (blue) in sliding levels of intensity (see colour scale bar). Genes within the network but without any changes are indicated as white circles. (**A**) DEGs are shown at 4 h (**B**), 6 h (**C**), 24 h and (**D**) 48 h following synchronised internalisation of POS cargos.

Next, we used Cytoscape (https://cytoscape.org/, accessed on 31 August 2024) to visualise specific genes that were significantly altered as a consequence of POS exposure across the three pathways. Scrutiny of constituent genes in the autophagy–lysosomal pathway revealed a pattern of mRNA changes associated with POS trafficking and degradation. These were broadly aligned with the earlier (4 and 6 h) vs. the later (24 and 48 h) timepoints following initiation of the pulse-chase assay. Direct comparisons were made between POS-fed RPE cultures vs. controls to identify transcriptomic changes specifically associated with POS trafficking and processing from baseline homeostatic activity at each timepoint. Accordingly, visualisation of colour-coded genes in POS-fed RPE vs. control cultures across each experimental timepoint (Figure 4 and Supplementary Figure S2) revealed the identity of genes that were either upregulated (red) or downregulated (blue). 

Four hours following synchronised POS internalisation, *HRAS*, *KRAS*, *NRAS*, *RHEB*, *PPP2CA*, *ULK2*, *ATG101*, *CAMKK2*, *WIPI1*, *RUBCN*, *ATG16L1* and *RAB7B* were upregulated compared to control RPE. Upregulated genes could be visually identified, either transitioning from lighter to darker red or switching from blue to red. By contrast, *PTEN*, *ATG4C*, *VAMP8*, *CSTD* and *MLST8* were downregulated. These are visible either transitioning from lighter to darker blue or switching from red to blue. The remaining colour-coded genes showed no changes when exposed to POS (Figure 4A and Supplementary Figure S2). At 6 h post POS exposure, *HRAS*, *KRAS*, *NRAS*, *RHEB*, *ULK1*, *ULK2*, *PPP2CA*, *ATG101*, *WIPI1*, *CAMKK2*, *BCL2*, *RUBCN*, *ATG16L1* and *ATG4B*, were upregulated compared to controls. However, *MLST8*, *ATG4A*, *ATG4C*, *RAB7B* and *CTSD* were downregulated compared to POS-naïve controls (Figure 4B and Supplementary Figure S2). Genes that were upregulated at 6 h from the 4 h timepoint in POS-exposed RPE included *ULK2* and *ATG4B*. Downregulated genes included *NRAS*, *PPP2CA RHEB*, *ATG16L1* and *ATG4A*. Genes that showed a similar pattern of transcriptional expression in control RPE, being also either up- or downregulated, were excluded. In this way, only genes that demonstrated a clearly distinct transcriptional profile from baseline homeostatic conditions were considered for further scrutiny in POS-exposed RPE cells. Furthermore, DEGs with ≤50 read counts between timepoints of POS-exposed RPE were also excluded (Supplementary Table S5), as these were unlikely to be associated with any significant changes, particularly at the protein level. Twenty-four hours following initiation of the pulse-chase assay, *NRAS*, *RHEB*, *PPP2CA*, *MLST8*, *WIPI1*, *ATG4A*, *ATG4C* and *CTSD* were upregulated compared to POS-naïve RPE, whilst *ULK1*, *ULK2*, *BCL2*, *RUBCN*, *ATG4B* and *VAMP8* were downregulated (Figure 4C and Supplementary Figure S2). At the final timepoint of 48 h following POS exposure, *HRAS*, *ULK1*, *ATG101*, *AMBRA1*, *ATG4B*, *RAB7B*, *VAMP8* and *CSTD* were upregulated compared to controls. By contrast, *PTEN*, *KRAS*, *ULK2*, *BCL2*, *RUBCN* and *ATG4C* were downregulated (Figure 4D and Supplementary Figure S2). Genes that were upregulated at48 h from the previous 24 h timepoint in POS-exposed RPE included *ULK1* and *ATG4B*, whilst downregulated genes included *KRAS* and *ATG4C*. In summary, our studies revealed a pattern of gene orchestration in response to POS trafficking and degradation in RPE cells, the implications of which will be discussed in the following sections. 

In order to assess transcriptomic changes in parallel proteolytic processes of the ubiquitin proteasome system and the chaperone-mediated pathway, we carried out an initial screening of constituent genes that were differentially expressed at an adjusted value of *p* < 0.01 between control vs. POS-fed RPE within each timepoint. This threshold did not identify any targets in either pathway. We therefore compared read counts at an adjusted value of *p* < 0.01 between different experimental timepoints. Genes encoding components in the different steps involved in delivering cargos to the proteasome were grouped and then colour-coded as before to highlight baseline/homeostatic activity levels compared to those in POS-exposed RPE at each timepoint (Supplementary Figure S3). Our data showed the temporal upregulation and downregulation of genes in distinct clusters of the ubiquitin proteasome system. At 4 h, we observed a broad pattern of mRNA upregulation in genes encoding components of the ubiquitin proteasome system in RPE exposed to POS compared to controls. Some evidence of this persisted until 6 h, although these differences were abolished at 24 and 48 h. A similar analysis of genes encoding the chaperone-mediated machinery also revealed a broad pattern of mRNA upregulation at 4 h in RPE exposed to POS compared to controls. This was less evident at 6 h, after which no marked differences were observed between the two groups (Supplementary Figure S4). Collectively, these findings show evidence of cross-talk between the autophagy–lysosomal pathway with the ubiquitin proteasome system and the chaperone-mediated pathway at early timepoints following POS internalisation. 

### 3.4. A Targeted Scrutiny of Genes Related to the Autophagy–Lysosomal Pathway Provides Additional Insights into POS-Induced Transcriptomic Changes

**Figure 5:**
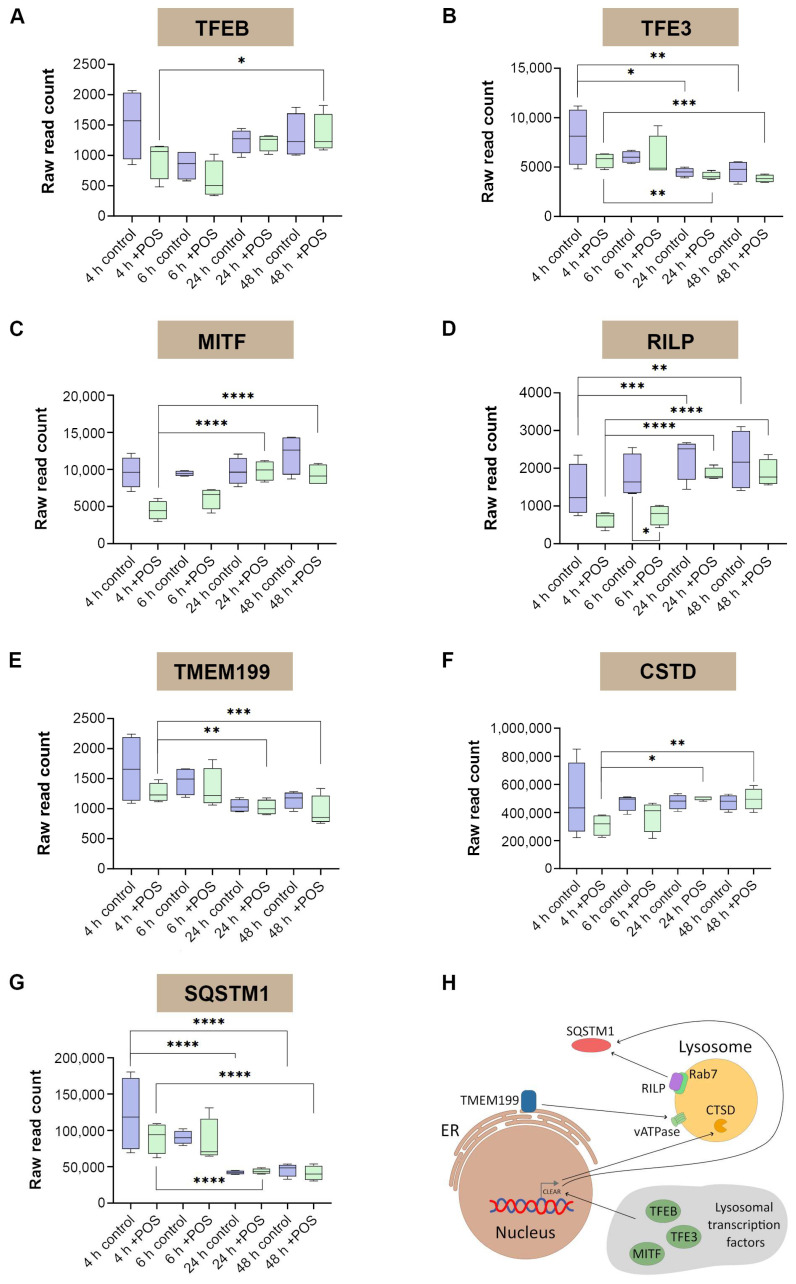
Analyses of a subset of genes known to be regulators of the autophagy–lysosomal pathway showing raw mRNA read counts across four biological replicates. (**A**) Following exposure to photoreceptor outers segments (POS), the transcription factor EB (*TFEB*) showed significant upregulation at 48 h compared to levels at 4 h. (**B**) At 24 h, homeostatic levels of the transcription factor binding to IGHM enhancer 3 (*TFE3*) were diminished compared to those at 4 h. This pattern was also recorded in RPE cells exposed to POS at identical timepoints. (**C**) At 24 and 48 h, levels of the melanocyte inducing transcription factor (*MITF*) were elevated compared to those at 4 h in RPE exposed to POS. (**D**) A similar pattern was also observed in the Rab-interacting lysosomal protein (*RILP*) at identical timepoints following POS exposure. However, upregulation of mRNA levels was also noted in RPE cells under homeostatic conditions between 48 vs. 4 h. *RILP* levels in POS-exposed RPE after 6 h were diminished compared to levels in controls at the same timepoint. (**E**) Levels of the transmembrane protein 199 (*TMEM199*) were downregulated at 24 and 48 h compared to 4 h following POS exposure. (**F**) Levels of lysosomal cathepsin D (*CTSD*) were elevated at 24 and 48 h compared to 4 h following POS exposure. (**G**) Similarly, sequestosome 1 (*SQSTM1*) mRNA levels were also diminished at these timepoints as a function of POS. Statistical comparisons were made using a Wald test following a negative binomial generalised linear model, where significant differences are indicated as *p* ≤ 0.05 (*), *p* ≤ 0.01 (**), *p* ≤ 0.001 (***) and *p* ≤ 0.0001 (****). (**H**) A schematic diagram showing reported interactions between these genes.

Given the extent of mRNA level responses to POS trafficking and processing in the autophagy–lysosomal pathway, we scrutinised a subset of related genes in this system for evidence of further change. Prominent amongst these are genes of the microphthalmia/transcription factor E (MiTF/TFE) family, which is composed of several conserved and closely related members consisting of the transcription factor EB (TFEB), TFE3, TFEC and the microphthalmia-associated transcription factor (MITF). These proteins are notable for their roles as master regulators of lysosomal function and autophagy. Scrutiny of raw mRNA read counts across four biological replicates revealed *TFEB* levels to considerably vary across all experimental conditions and timepoints, with a significant upregulation noted at 48 h following POS exposure compared to levels at 4 h (Figure 5A).

For *TFE3*, we observed that baseline mRNA levels in POS naïve RPE were diminished at 24 and 48 h compared to the initial 4 h timepoint. A similar pattern was observed in RPE exposed to POS at equivalent timepoints compared to 4 h (Figure 5B). However, *MITF* levels were significantly elevated at 24 and 48 h compared to 4 h as a result of POS exposure (Figure 5C), whilst *TFEC* showed no discernible changes in our study (Supplementary Figure S5). We also scrutinised the Rab-interacting lysosomal protein (RILP). *RILP* mRNA levels in POS-fed RPE were significantly elevated at 24 and 48 h compared to the initial 4 h timepoint. Interestingly, some aspects of this pattern were also observed under POS-naïve conditions. Nonetheless, RILP levels were also significantly downregulated at the early timepoint of 6 h when exposed to POS compared to homeostatic conditions (Figure 5D). We also scrutinised mRNA read counts of the transmembrane protein 199 (TMEM199), an assembly factor for the vATPase proton pump which regulates lysosomal acidification. Compared to 4 h, *TMEM199* levels at 24 and 48 h were downregulated in RPE exposed to POS cargos (Figure 5E). Next, we examined the fate of lysosomal cathepsin D (CTSD), which is primarily responsible for the efficient degradation of internalised POS cargos in RPE cells. *CTSD* levels at 24 and 48 h were elevated compared to those at 4 h in RPE exposed to POS molecules (Figure 5F). Sequestosome 1 (SQSTM1/p62) is a multifunctional signalling protein which recruits ubiquitinated cargos to autophagosomes. SQSTM1/p62 levels are therefore widely used as an indicator of autophagic flux [40]. Our findings show that *SQSTM1* levels in control and POS-fed RPE were diminished at 24 and 48 h compared to those at 4 h (Figure 5G). A schematic diagram depicting known interactions between these targets is shown (Figure 5H) which provides further insights into these mechanisms. 

## 4. Discussion

A defining feature of the RPE monolayer is its high proteolytic burden, which over time leads to the accumulation of pathogenic lipofuscin molecules and its derivatives that contribute to RPE dysfunction and atrophy linked with retinopathy [7]. The internalisation of POS by RPE cells as part of the daily photoreceptor renewal cycle necessitates the timely degradation of these cargos, the efficiency of which gradually declines with age. This was evidenced in early histopathological studies, which showed RPE from healthy donors containing high levels of lipofuscin and related macromolecules within the cell cytoplasm that appear as well-defined inclusions [41,42]. We and others have since described how POS cargos are internalised via receptor-mediated mechanisms and trafficked for processing in the phagosome and autophagy–lysosomal pathway [7,8,43]. By exploiting a human *in-vitro* RPE cell model, our previous studies delineated the timepoints at which POS cargos maximally co-localised with Rab5 and Rab7 vesicles (early and mature phagosomes/endosomes, respectively), LAMP1 (immature) and LAMP2 (mature) lysosomes as well as LC3b (autophagic) compartments [12]. The effects of oxidative stress, impaired membrane fusion/autophagy and exposure to oxidatively modified POS (OxPOS) or Ab, were found to substantially affect these processes [12,13,14]. These findings also revealed the extent of plasticity within the cargo-trafficking pathways of healthy RPE, indicating their resilience to age-related changes and the onset of retinopathy. This led us to an *a priori* hypothesis that POS processing may also elicit a cascade of events at the transcriptomic level, which we sought to investigate in this study. 

To enable such an analysis, we scrutinised whole mRNA-level changes at timepoints at which POS cargos were known to maximally co-localise with Rab5, Rab7, LAMP-positive lysosomes and LC3b vesicles [12,13]. Studies of this kind can benefit from exploiting *in-vitro* approaches, where RPE cells can be sourced directly from primary tissues (typically of mouse, porcine or human origin), from donor stem cell-derived or from RPE cell-lines, which have been used in the past as effective surrogate models of the native/*in-situ* RPE monolayer. For instance, previous work by others has shown how highly differentiated monolayers of low-passage ARPE-19 cells that were cultured long-term on transwell membranes recapitulated the global mRNA characteristics of native RPE [39]. This included the expression of genes and proteins involved in the receptor-mediated phagocytosis of POS molecules. Our studies, which were carried out using similar ARPE-19 culture conditions and in four biological replicates, yielded 44,909 genes with 83.35% alignment with the human reference genome. An assessment of genes specifically associated with the identity of RPE cells revealed the expressed transcripts of all the tested targets under *in-vitro* culture conditions. Furthermore, included amongst the list of expressed transcripts were a majority of the listed AMD-linked genes (81.7%). The remainder may be attributed to mRNA originating from other cell-types or tissues, for instance from the neuroretina or the choroid, which are also important for the development of AMD [44,45]. Some of these transcripts may indeed be expressed in RPE cells but only under pathogenic conditions. As many IRDs involve genetic changes in the RPE [2,46], we also ascertained the transcriptional profile of IRD-associated RPE-specific genes in our study. We found that a majority of these RPE-specific genes (72%) were also expressed in highly differentiated ARPE-19 cultures. Collectively, the expression of AMD and IRD-associated RPE transcripts further support the idea that *in-vitro* RPE cells faithfully recapitulate the genetic profile of its native counterpart to a high degree. Another feature of post-mitotic RPE is their capacity to undergo cell division, which has been reported in peripheral RPE of the rodent eye as cell cycle active [47]. The proliferative potential of RPE cells can also be observed during retinal detachment associated with complications such as proliferative vitreoretinopathy or hyperplastic RPE in AMD eyes [48,49]. However, highly differentiated RPE monolayers in long-term culture conditions that are used in our studies are not expected to be cell cycle-active. We wanted to confirm this at the mRNA level and therefore looked for evidence of cell cycle-associated DEGs in controls vs. RPE cells exposed to POS. We found no evidence of any cell cycle-related activity at the transcriptional level, indicating that (1) such monolayers, once established, are highly stable, in line with their high level of structural specialisation, and (2) the synchronised internalisation and processing of POS does not elicit any notable cell cycle-related activity under *in-vitro* conditions. 

Assessment of the global transcriptional profile by PCA revealed insights into mRNA regulation during homeostatic activities under long-term *in-vitro* conditions in healthy POS-naïve (control) RPE. Irrespective of POS exposure, all genes in our dataset segregated into two broad groups consisting of 4 and 6 h vs. 24 and 48 h. These findings appear to largely reflect underpinning changes to the RPE transcriptome as a function of time. However, of note, significantly more genes were globally up- or downregulated due to POS exposure. Although elucidating the identity of these genes via an unbiased approach is of great interest, herein we focused solely on investigating potential changes to genes that were specifically associated with proteolytic activities of the RPE, which was our objective at the outset. A significant body of work has identified genes that encode the machinery which comprise cellular proteostasis. Collectively referred to as the proteostasis network, ageing as well as various diseases including retinopathies are associated with failed proteostasis [50,51,52,53,54]. Perhaps unsurprisingly, several genes in the proteostasis network also mapped to those linked with AMD [55,56,57,58,59] and IRDs [60,61] in our dataset. Comparing our dataset with this comprehensive list of all known proteostasis genes enabled us to specifically relate our findings to its broadest but most relevant components (2905 of almost 3000 non-redundant genes). Genes listed in this repository included those that may regulate proteostasis at either the transcriptional or translational level. A further breakdown showed genes in (1) the autophagy–lysosomal pathway, (2) the ubiquitin proteosome system and (3) the chaperone-mediated pathway, inclusive of genes shared between these pathways. As recent studies showed evidence of cross-talk between these pathways [50,62], we were keen to undertake a more holistic assessment of any POS effects in the wider proteostasis network, rather than focusing solely on the autophagy–lysosomal pathway. Cross-talk may occur between distinct proteolytic mechanisms under conditions of stress such as proteinopathy. For instance, activities in a specific pathway may be enhanced by recruiting resources from shared proteolytic components, resulting in the concomitant downregulation of parallel degradative mechanisms which are less critical to this task. 

**Figure 6:**
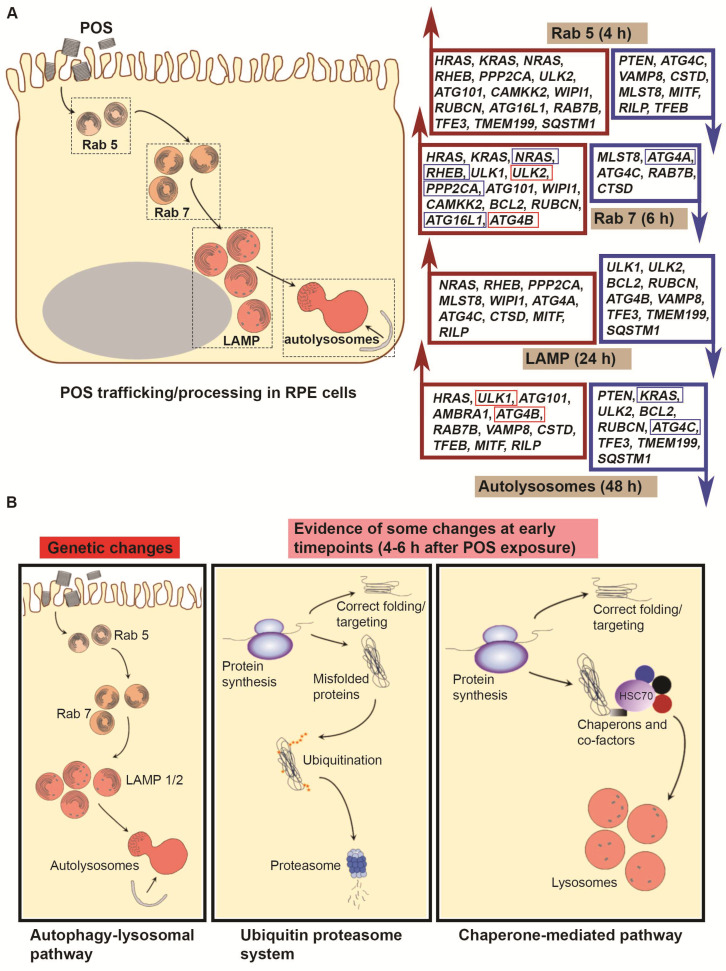
Schematic overview of proteolytic pathways and summary findings. (**A**) Summary of differentially expressed genes (DEGs) identified in the phagosome/endosome and autophagy–lysosomal pathways that are upregulated (red) or downregulated (blue) in response to POS trafficking and processing compared to control RPE. Differentially expressed transcriptional patterns from the previous timepoint are indicated by red (upregulation) or blue (downregulation) boxes. These broadly follow a pattern consistent with the temporal dynamics of POS trafficking and processing, revealing new links between cargo handling and transcriptome-level responses in RPE cells. (**B**) Proteolytic pathways that function in parallel with potential for cross-talk. The autophagy–lysosomal pathway internalises shed photoceptor outer segments (POS) daily from overlying photoreceptors which are subsequently trafficked for degradation in autolysosomes. By contrast, misfolded proteins are degraded via the ubiquitin–proteosome system. Here, polyubiquitinated proteins are targeted to the proteosome, where the muti-subunit 26S complex recognises, unfolds and degrades these substrates into smaller peptides. In the chaperone-mediated pathway, target proteins are recognised via the KFERQ or similar motifs by the heat shock cognate protein 70 (Hsc70) and guided to lysosomes alongside other chaperones for degradation. Even though POS cargos are trafficked and degraded in the autophagy lysosomal pathway, our findings reveal an initial upregulation of genes in the ubiquitin–proteosome system and the chaperone-mediated pathway at 4–6 h following POS entry into RPE cells.

The use of Cytoscape to visually map genes in the autophagy–lysosomal pathway, which was of primary interest, identified DEGs showing their relative levels of up- or downregulation in response to POS trafficking and degradation. An analysis of genes at 4 h following synchronised POS internalisation revealed the upregulation of Ras family members *HRAS*, *KRAS* and *NRAS*, which function as molecular switches, cycling between active/inactive (GTP/GDP), and regulating highly conserved signalling pathways such as PI3K-AKT-mTOR that influence endocytosis and membrane trafficking [63] (Figure 6A). 

*RHEB*, which was also upregulated, acts as a potent activator of mTORC1 kinase activity [64]. *PPP2CA*, another upregulated gene, mediates the removal of phosphate groups from serine and threonine residues and is involved in signal transduction, whilst *ULK2* is involved in autophagy initiation. Both *ATG101* and *ATG16L1* are involved in autophagy and in regulating elongation of the nascent autophagosomal membrane [65]. *ATG101* is involved in autophagosome assembly and stabilises ATG13 against proteasomal degradation [66]. Additionally upregulated genes such as *CAMKK2* mediate the second messenger effects of Ca^2+^ and serve as an upstream kinase of AMPK, an evolutionarily conserved cellular energy sensor. Previous studies have shown beneficial effects of plant extracts in RPE cells via the activation of CAMKK2/AMPK [67,68]. We also observed the upregulation of *WIPI1* which encodes a WD40 repeat protein that co-localises to the plasma membrane, the ER and the nuclear envelope that are thought to act as sources of membrane for autophagosome formation [69]. The upregulation of *RUBCN* which encodes Rubicon was noteworthy, as it acts as a Rab7 effector to ensure the correct progression of the endocytic pathway and is a negative regulator of autophagosome maturation [70,71]. Interestingly, Rubicon expression was reportedly highest in the morning following POS internalisation. Here, RPE cells promote the non-canonical autophagy process termed LC3-associated phagocytosis (LAP) through supressing autophagy via activation of the epidermal growth factor receptor [72]. Perhaps it was therefore unsurprising that we also recorded the upregulation of *RAB7B* which encodes a protein that regulates endosome to Golgi transport, but also co-localises to late endosomes and lysosome-associated compartments [73]. Our previous studies showed POS cargos to be maximally co-localised within Rab7 compartments in healthy RPE cells after 6 h post internalisation. We also reported the presence of hybrid vesicles that were positive for both Rab7 and lysosomal markers, showing evidence of Rab7 maturation [12]. However, most antibodies against Rab7 label Rab7A rather than Rab7b. Although Rab7b is perhaps comparatively less well-characterised, vesicles labelled with Rab7b can translocate cargos such as the Toll-like receptor 4 for degradation in lysosomes [73]. Interestingly, we found higher mRNA counts for *RAB7A* compared to *RAB7B* across all four timepoints in POS-exposed RPE. However, significant differences (DEGs) were only noted for the latter. A series of genes regulating lysosomal and autophagic activity consisting of *TFE3*, *TMEM199* and *SQSTM1* were also upregulated at 4 h. Although their mRNA read counts were indistinguishable from control cultures, perhaps indicating homeostatic autophagic tick over, these showed a significant decline in response to POS exposure at subsequent timepoints. 

We also recorded the downregulation of genes *PTEN*, *ATG4C*, *VAMP8*, *MLST8* and *CSTD* at the 4 h timepoint (Figure 6A). Of these, *PTEN* encodes a protein that is a part of the PI3K/AKT/mTOR pathway, where its regulation has been shown to affect RPE survival [74]. *ATG4C* encodes a member of the autophagin protein family required for autophagic responses under cellular stress [75]. The vesicle-associated membrane protein 8 (VAMP8) is a SNARE involved in the formation of autophagosomes via fusion with lysosomes [76]. *MLST8* encodes a subunit that is part of the mTORC1 and mTORC2 complexes which are integral in autophagy [77]. Of note, we recorded the downregulation of *CSTD* that encodes a pre-proprotein which is processed to generate multiple products, including the cathepsin D light and heavy chains that heterodimerise to form the mature enzyme [78,79]. Lysosomal cathepsin D exhibits pepsin-like activity and is the primary enzyme responsible for POS degradation in RPE cells [80,81,82,83]. Initially, at the 4 h timepoint, *CSTD* levels in control and POS-fed RPE cultures were at low levels but increased significantly at subsequent timepoints as a function of POS. Interestingly, we also recorded the downregulation of *MITF*, *RILP* and *TFEB* at 4 h, which play important roles in lysosomal function and autophagy that occur at later timepoints (24 h onwards after POS exposure). In summary, we report a pattern where following the receptor-mediated internalisation of POS cargos, genes involved in upstream signalling events, promoting endosome maturation and early stages of cargo trafficking were broadly upregulated, concomitant with the downregulation of specific elements which play a role in terminal stages of the autophagy–lysosomal pathway.

The next timepoint of 6 h was also selected based on our previous work, which showed POS cargos in healthy RPE to be maximally co-localised to Rab7 vesicles, corresponding to mature phagosomes/endosomes [12,13]. As this was only two hours from the first assessment point, we did not anticipate any wholesale changes to the transcriptomic landscape. Indeed, we observed the upregulation of the same genes recoded at the earlier 4 h timepoint with the exception of *ULK2, BCL2* and *ATG4B* which had since increased (Figure 6A). *ULK1*, similar to *ULK2* that was upregulated at 4 h, encodes serine/threonine–protein kinases that regulate the formation of autophagophores, which are precursors of autophagosomes. Both proteins also act as negative regulators of mTORC1 and connect with proteins encoded by *ATG*, *RUBCN* and *WIPI1* [84]. *BCL2* encodes an outer mitochondrial membrane protein which interacts with Becn1 and Ambra1 to inhibit their autophagic functions [85]. Compared to 4 h, *ATG4B* which encodes the autophagy-related 4B cysteine peptidase, also showed higher levels of upregulation at 6 h. This protein is required for canonical and non-canonical forms of autophagy, including mitophagy [75]. Interestingly, at the 6 h timepoint, *TFE3*, *TEMEM199* and *SQSTM1* were no longer upregulated as before. Genes that were still downregulated at the 6 h period included *MLST8*, *ATG4C*, *RAB7B* and *CTSD* (Figure 6A). However, we found additionally downregulated genes including *ATG4A*, whilst *PTEN*, *VAMP8* and the lysosomal/autophagy activators *MITF*, *RILP* and *TFEB* were no longer diminished compared to the 4 h timepoint. *ATG4A* encodes a cysteine protease that plays a key role in processing Atg8 for conjugation to phosphatidylethanolamine in autophagosome biogenesis [86]. *NRAS*, *PPP2CA RHEB*, *ATG16L1* and *ATG4A* were downregulated at 6 h compared to their levels at the 4 h timepoint. Although this dataset was obtained only 2 h after the first 4 h assessment point, it revealed a gradually changing transcriptional landscape, with increased signs of transitioning to mechanisms favouring autophagy.

As the global PCA and subsequent studies showed a clear distinction between 4 and 6 h vs. 24 and 48 h readouts, we anticipated marked differences in the transcriptional profile to emerge at the 24 h timepoint. Here, *NRAS*, *RHEB*, *PPP2CA* and *WIPI1* were still upregulated in POS-exposed RPE as before. However, *ATG4A*, *ATG4C* and *CTSD* that were diminished at 6 h were now upregulated (Figure 6A). Lysosomal- and autophagy-promoting genes *MITF* and *RILP* were also upregulated, showing significant increases in read counts compared to the 4 h timepoint. By contrast, *ULK1*, *ULK2*, *BCL2*, *RUBCN*, *ATG4B* and *VAMP8* were downregulated at 24 h in POS-fed RPE. *ATG101* which had higher read counts at 4 and 6 h switched to being downregulated from 24 h onwards in POS-exposed RPE. Compared to the 4 h timepoint, genes such as *TFE3*, *TMEM199* and *SQSTM1* were also downregulated. These findings appear consistent with cargos transitioning from upstream stages of the endocytic-trafficking pathway to degradative stages focused on lysosomes, where our previous studies showed POS to be maximally co-localised to immature and mature lysosomes by confocal microscopy [12,13]. Scrutiny of genes at the 48 h timepoint in POS-exposed RPE showed *HRAS*, *VAMP8* and *CSTD* to be still upregulated as they were at the previous 24 h timepoint (Figure 6A). Interestingly, genes that were diminished at 4 h (*ATG4C*, *VAMP8*, *MITF*, *TFEB* and *RILP*) and at 6 h (*RAB7B*) had switched to being upregulated at the 48 h timepoint. Some genes that were diminished at 24 h (*ULK1* and *ATG4B*) were also upregulated. Furthermore, we recorded the addition of *AMBRA1* to the list of upregulated genes at the 48 h timepoint in POS-exposed RPE. *AMBRA1* encodes the autophagy and beclin-1 regulator-1, where following the induction of autophagy, the protein is released from dynein and translocated to the ER for autophagosome formation [87]. Both *MITF* and *RILP* remained upregulated at 48 h as they were at the 24 h timepoint. However, *TFEB* showed a marked upregulation at 48 h compared to 4 h after POS exposure. The list of downregulated genes included *PTEN*, *KRAS*, *ULK2*, *BCL2*, *RUBCN* and *ATG4C* (Figure 6A). Interestingly, these include genes that switched from being upregulated at 4 h (*KRAS* and *RUBCN*), 6 h (*ULK2*) or 24 h (*ATG4C*), which were involved in earlier stages of cargo-trafficking. *TFE3*, *TMEM199* and *SQSTM1* were also diminished at the 48 h timepoint compared to their read counts at 4 h, suggesting that the increased synthesis of autophagy-related components was no longer necessary. By contrast, *ULK2* and *ATG4B* were further upregulated, whilst *KRAS* and *ATG4C* were comparatively downregulated at 48 h relative to their levels at the 24 h timepoint. Collectively, these findings showed evidence of silencing specific genes that were upregulated during earlier stages of POS trafficking in favour of promoting cargo degradation processes in autolysosomes of RPE cells.

Although the trafficking and processing of POS cargos in RPE cells are mediated via the phagosome and autophagy–lysosomal pathway [7], our findings revealed that POS entry also initiated a cascade of mRNA level responses in parallel proteolytic systems (Figure 6B), at least within the initial 4–6 h period. To our knowledge, this is the first detailed report of such a finding, which was facilitated by the highly manipulable nature of our *in-vitro* approach. By exploiting this model, we were not only able to identify broad patterns but also individual gene expression and gene-clusters involved in cross-talk between distinct proteolytic networks. Indeed, the interplay between the ubiquitin proteasome system and autophagy was reported in the retina, with E3 ligases in the ubiquitination pathway being targeted for degradation by autophagy [88]. Interestingly, genes encoding the E3 ring were also amongst those upregulated by the entry of POS in our study. These findings shed further light into reports of cross-talk between different proteolytic systems within cells, allowing for compensatory mechanisms and interconnected regulatory responses in maintaining cellular homeostasis [89,90]. Such evidence of mRNA level upregulation in other proteolytic systems in response to POS entry suggests an initial uptick of global degradative capacity upon cargo internalisation in otherwise POS-naïve cells. However, this scenario is unlikely to occur in the living retina, where RPE cells degrade POS on a daily basis [7]. Nonetheless, the identification of individual genes and points of interaction between different proteolytic networks provide useful insights into how advanced age and onset of retinopathy can alter the transcriptional profile of RPE cells.

A caveat to this study is that our findings are limited to transcriptomic responses, defined as coding mRNAs, rather than any direct genetic changes that may occur such as alterations to methylation patterns. Nonetheless, our findings invite further scrutiny into mechanisms where impaired proteostasis contributes to a clinically well-defined pathway of RPE atrophy leading to blindness. Whether such responses also result in an uptick of proteolytic responses at the protein level remains to be determined. Such studies could unravel new links between mRNA-level responses vs. protein synthesis under homeostatic and disease conditions, as these are not necessarily correlated. In fact, evidence suggests partial buffering between these steps, enabling rapid protein synthesis in response to stimuli [91]. Interestingly, differentially expressed mRNAs correlate significantly better with their protein product compared to non-differentially expressed mRNAs [92], supporting the idea that at least some responses at the protein level can coincide with transcriptomic changes as a function of POS-trafficking/processing in the RPE. Finally, we used the DAVID bioinformatics tool to independently map identified gene ontology networks to their respective molecular functions (Supplementary Table S9). These revealed the upregulation of GTPase activity and GTP/GDP binding alongside ubiquitin-like protein transferase activity, concomitant with the downregulation of cysteine peptidase and endopeptidase activities at early timepoints (4–6 h) following POS exposure. By contrast, the molecular functions of genes identified at later timepoints (24–48 h) showed an upregulation of cysteine peptidase and endopeptidase activities, coinciding with the downregulation of DNA regulatory mechanisms, with the latter likely indicating cessation of autophagy-related activity once POS breakdown has commenced. This unbiased scrutiny of the transcriptional landscape based on identified DEGs further supports our findings showing a direct correlation between cellular mechanisms involved in POS trafficking and degradation with corresponding gene expression changes in RPE cells.

## 5. Conclusions

In summary, we show that long-term cultures of highly differentiated ARPE-19 monolayers recapitulate the broad mRNA profile of *in-situ*/native human RPE, matching their semblance that was demonstrated at a structural level. However, ARPE-19 cells are not without limitations, and in this respect are no different to the challenges faced when using donor stem cell-derived RPE, where different cell lines can show clonal variability. Our studies demonstrated that ARPE-19 monolayers expressed AMD- and IRD-related transcripts but were quiescent for cell cycle-related genes. In line with our *a priori* hypothesis, the synchronised internalisation of POS elicited a cascade of transcriptome-level responses primarily in the phagosome and autophagy–lysosomal pathway which traffics and proteolytically degrades these molecules. This consisted of an uptick in mRNA-level responses of genes involved in the early stages of POS processing at 4 h, concomitant with the broad downregulation of genes in terminal stages of the pathway. A gradual change in the transcriptional landscape beginning from 6 h was followed by a broad reversal of fates favouring POS degradation, particularly by 48 h. This transcriptional map was remarkably consistent with the known co-localisation of POS cargos in different compartments of the phagosome and autophagy–lysosomal pathway. Only DEGs that showed a clearly distinct transcriptional pattern from homeostatic conditions were considered in our study. This high threshold enabled us to identify salient gene responses across time from what was otherwise a large dataset which showed smaller and likely insignificant variations in expressed transcripts. Furthermore, the potential of healthy RPE cells to increase their degradative capacity was demonstrated more widely, at least at the mRNA level, in parallel proteolytic systems in response to POS, providing specific evidence of cross-talk. Collectively, these findings not only reveal the identity of individual genes and gene networks involved in regulating an important homeostatic RPE function, but also provide wider knowledge of transcriptomic responses to cargo-trafficking and processing in cells.

## Data Availability

All data are included in this manuscript and in additional supporting material as Supplementary Figures and Tables. Reasonable requests for raw data will be considered by the authors before being made available to third parties.

## Supplementary Materials

The following supporting information can be downloaded at: https://www.mdpi.com/article/10.3390/cells14151166/s1, Figure S1: A principal component analysis (PCA) was carried out which revealed a broad pattern of segregation between 4 and 6 h vs. 24 and 48 h for all genes in the combined dataset (POS naive/control RPE + cells exposed to POS for all timepoints).; Figure S2: Visualisation of components in the autophagy-lysosomal pathway under homeostatic conditions in RPE cells, Cytoscape was used to map the autophagy-lysosomal network where differentially expressed genes (DEGs) are colour-coded to indicate their relative extent of upregulation (red) or downregulation (blue) in sliding levels of intensity, Genes in the pathway without any changes are shown in white, DEGs at 4, 6, 24 and 48 h are shown in control RPE cells naïve for photoreceptor outer segments (POS); Figure S3: Genes encoding components of the ubiquitin proteasome system were grouped and colour-coded to indicate their relative RNA levels. These were compared between control RPE (POS naive) vs. RPE cells exposed to POS. Timepoints (4, 6, 24 and 48 h) denote periods at which whole RNA was extracted from cultures; Figure S4: Genes encoding components of the chaperone-mediated pathway were grouped and colour-coded to indicate their relative RNA levels. These were compared between control RPE (POS naive) vs. RPE cells exposed to POS. Timepoints(4, 6, 24 and 48 h) denote periods at which whole RNA was extracted from cultures; Figure S5: Raw mRNA read counts of TFEC which encodes a protein, which alongside others, regulate lysosomal function and autophagy. Values for control (purple)vs. POS exposed (green) RPE cells are shown for 4, 6, 24 and 48 h timepoints. Table S1: RPE-specific genes; Table S2: AMD-related genes; Table S3: Inherited retinopathy genes in RPE cells; Table S4: Cell cycle-related genes; Table S5: Autophagy-lysosomal pathway genes; Table S6: Ubiquitin proteosome pathway genes; Table S7: Chaperone-mediated pathway genes; Table S8: Autophagy-lysosomal pathway genes showing significance within timepoint and between control vs. Pos exposure; Table S9: All upregulated and downregulated differentially expressed genes (DEGs) for each timepoint from Figure 6 were inputted into the UniProt ID mapping tool (https://www.uniprot.orgid-mapping). Gene ontology (GO) networks were subsequently identified mapping to their respective molecular functions using the Database for Annotation, Visualization, and integrated Discovery (DAVID) bioinformatics tool (https://davidbioinfommatics.nih.gov/). Genes are listed in the order following the most coverage to the least.
