# Supplementary material for: Whole RNA-Seq Analysis Reveals Longitudinal Proteostasis Network Responses to Photoreceptor Outer Segment Trafficking and Degradation in RPE Cells
Source: Cells. 2025 Jul 29;14(15):1166. doi: 10.3390/cells14151166 (PMC12346425; doi:10.3390/cells14151166)
Supplement: Supplementary file 1 [file cells-14-01166-s001.zip › Supplementary Material/Supplementary Figures.pdf]

Supplementary material

Supplementary figures:

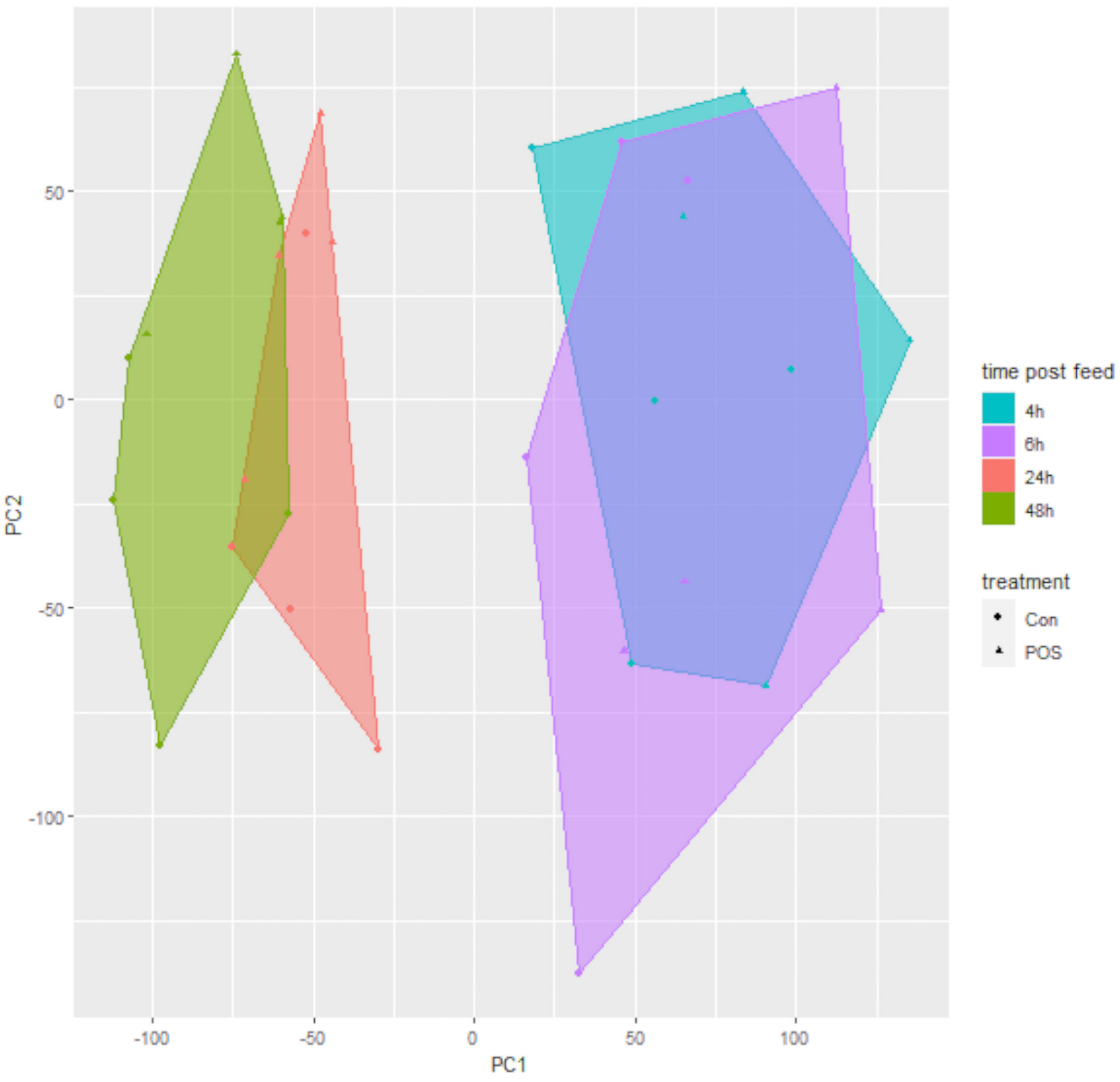

Supplementary Figure S1: A principal component analysis (PCA) was carried out which revealed a broad pattern of segregation between 4 and 6 hours vs. 24 and 48 hours for all genes in the combined dataset (POS naïve/control RPE + cells exposed to POS for all timepoints).

Autophagy-lysosomal pathway

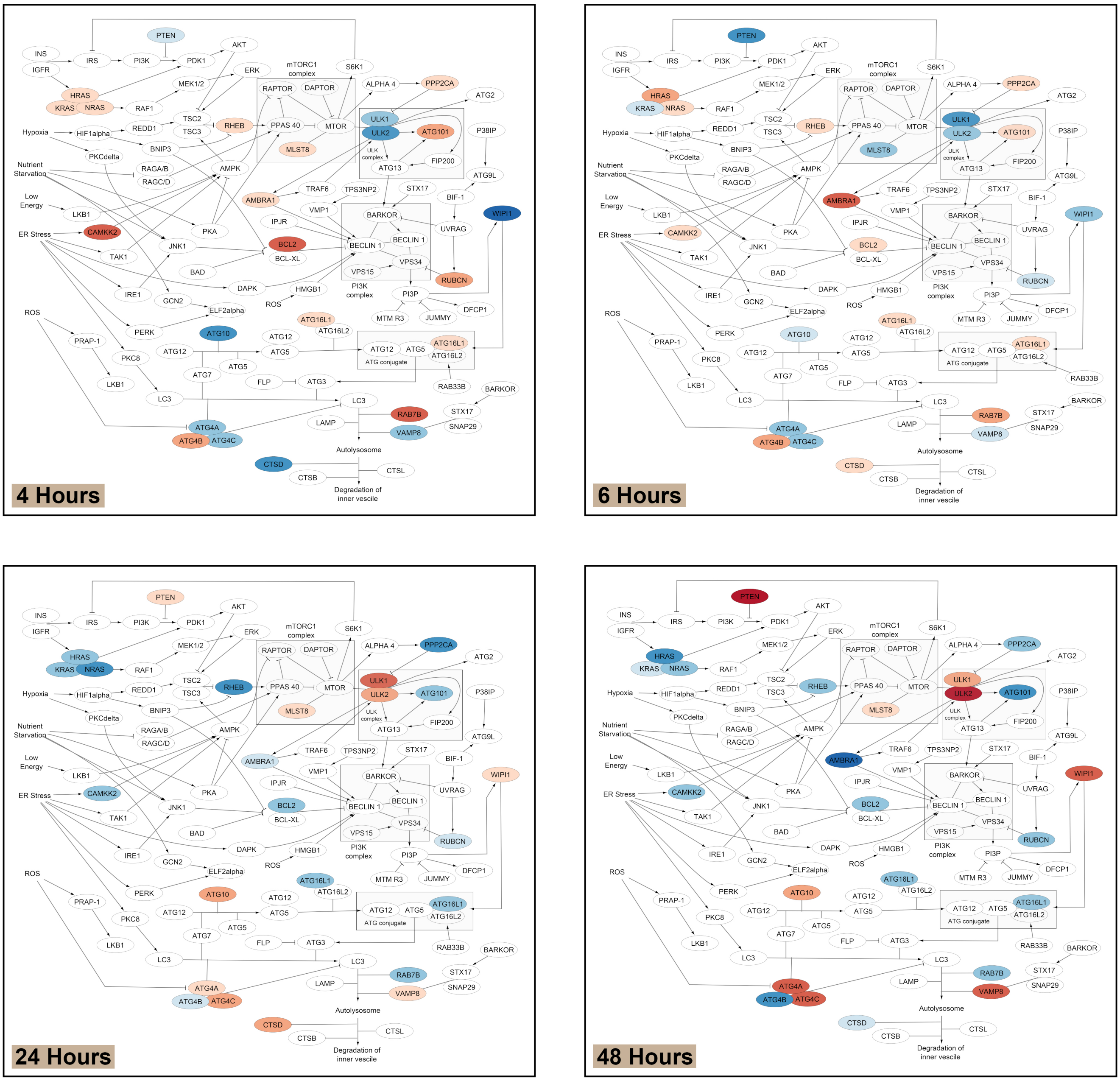

Supplementary Figure S2: Visualisation of components in the autophagy-lysosomal pathway under homeostatic conditions in RPE cells. Cytoscape was used to map the autophagy-lysosomal network where differentially expressed genes (DEGs) are colour-coded to indicate their relative extent of upregulation (red) or downregulation (blue) in sliding levels of intensity. Genes in the pathway without any changes are shown in white. DEGs at 4, 6, 24 and 48 hours are shown in control RPE cells naïve for photoreceptor outer segments (POS).

High expression ▬ **Low expression**

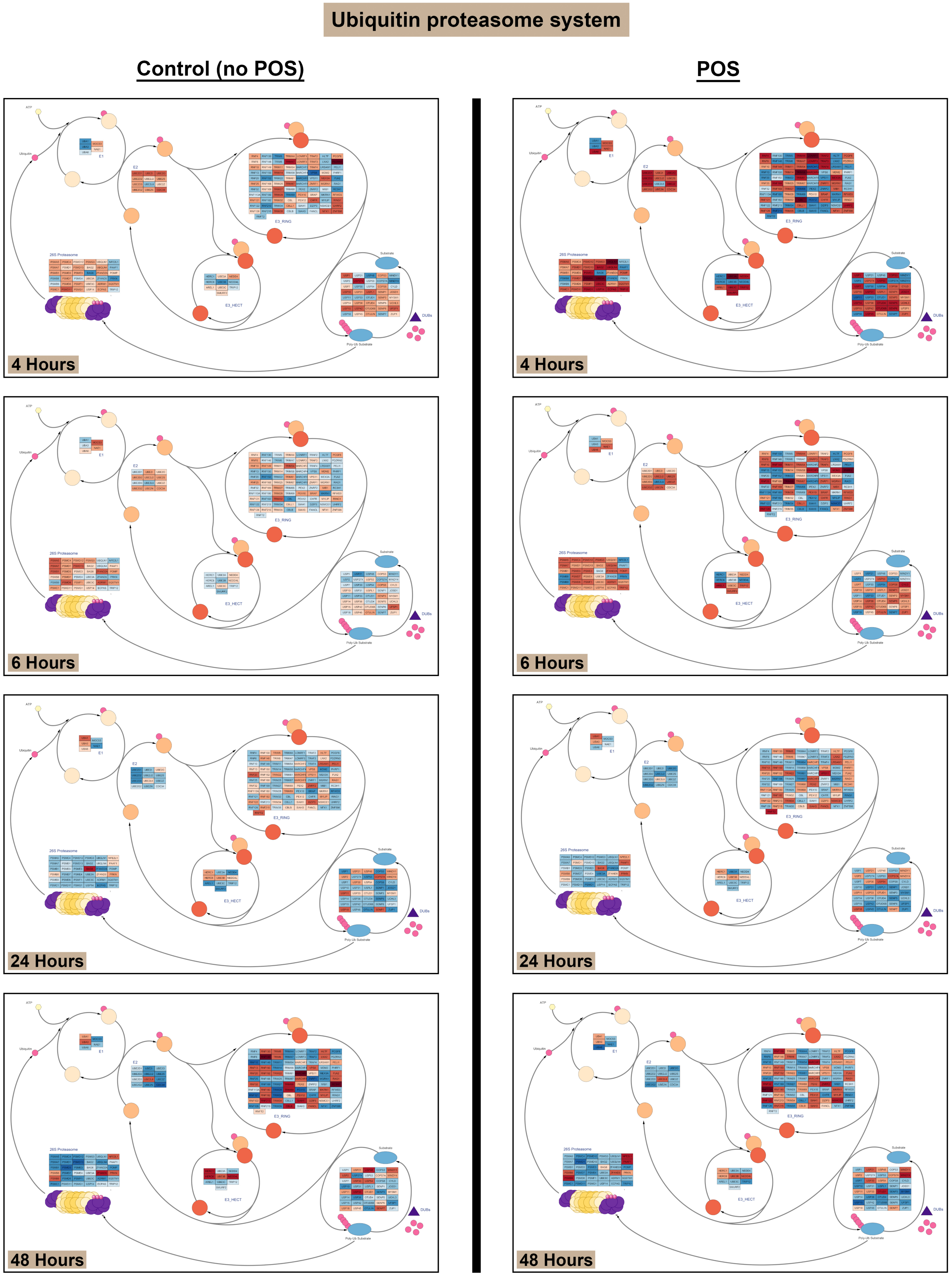

Supplementary Figure S3: Genes encoding components of the ubiquitin proteasome system were grouped and colour-coded to indicate their relative RNA levels. These were compared between control RPE (POS naïve) vs. RPE cells exposed to POS. Timepoints (4, 6, 24 and 48 hours) denote periods at which whole RNA was extracted from cultures.

High expression **Low expression**

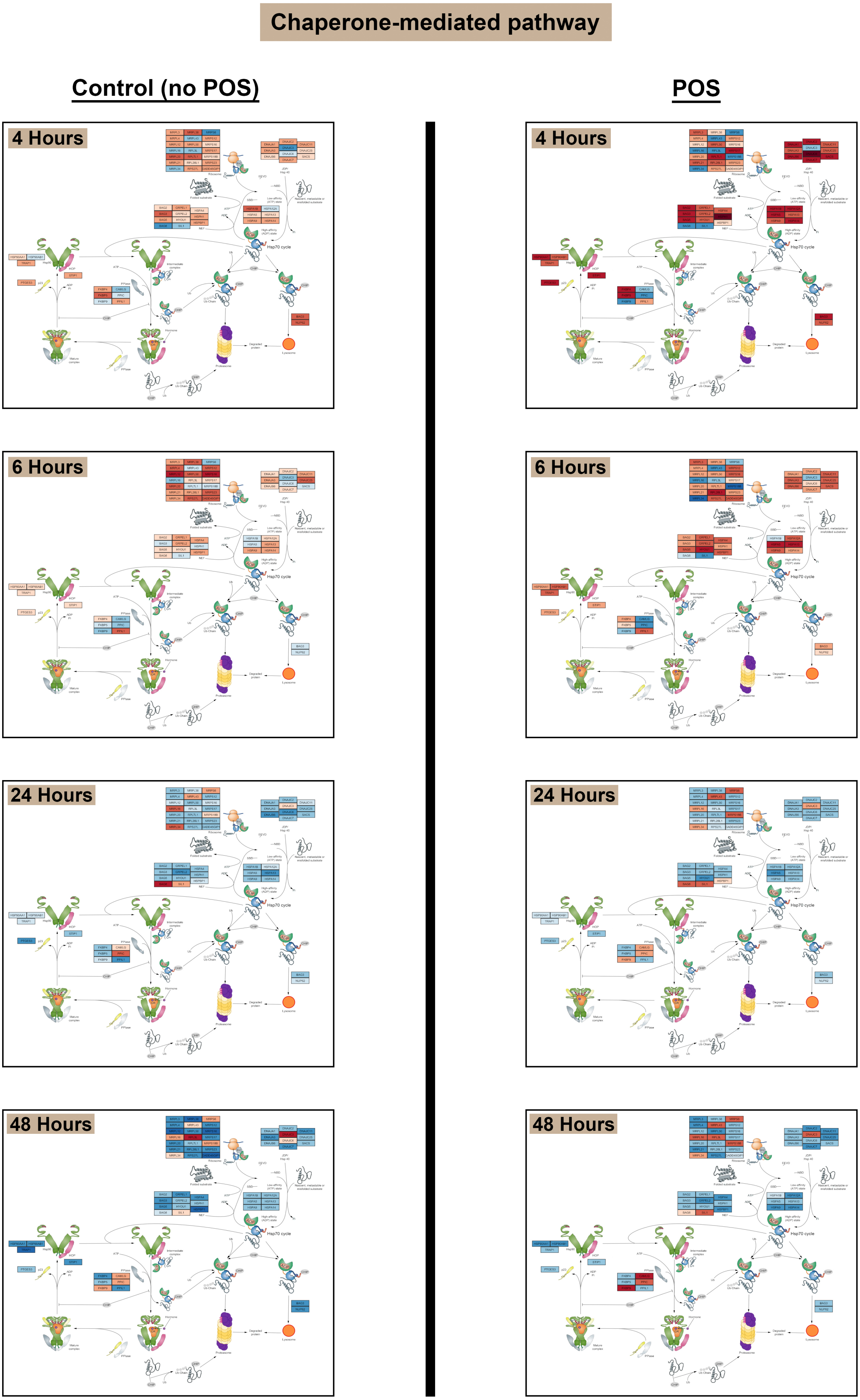

Supplementary Figure S4: Genes encoding components of the chaperone-mediated pathway were grouped and colour-coded to indicate their relative RNA levels. These were compared between control RPE (POS naïve) vs. RPE cells exposed to POS. Timepoints (4, 6, 24 and 48 hours) denote periods at which whole RNA was extracted from cultures.

High expression **Low expression**

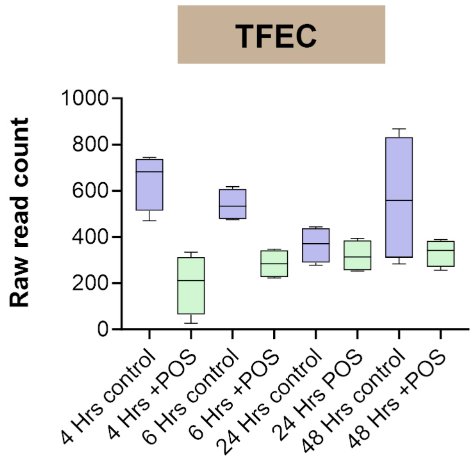

Supplementary Figure S5: Raw mRNA read counts of TFEC which encodes a protein, which alongside others, regulate lysosomal function and autophagy. Values for control (purple) vs. POS exposed (green) RPE cells are shown for 4, 6, 24 and 48 hour timepoints.
