# Supplementary material for: Whole RNA-Seq Analysis Reveals Longitudinal Proteostasis Network Responses to Photoreceptor Outer Segment Trafficking and Degradation in RPE Cells
Source: Cells. 2025 Jul 29;14(15):1166. doi: 10.3390/cells14151166 (PMC12346425; doi:10.3390/cells14151166)
Supplement: Supplementary file 1 [file cells-14-01166-s001.zip › Supplementary Material/Supplementary Tables.pdf]

Supplementary information

Mean count readings for each gene shown across 4 independent biological replicates where *in-vitro* RPE monolayers were exposed to a synchronised pulse-chase assay with isolated photoreceptor outer segments (POS).

Supplementary Table S1: RPE-specific genes

Gene	POS (4Hrs)	POS (6Hrs)	POS (24Hrs)	POS (48Hrs)
BEST1	879.0493	873.5358	893.9675	1204.543
CLDN3	388.2502	404.994	823	1148.566
LRAT	9.735207	10.74171	30.47774	64.08077
OCA2	344.9042	343.0306	1022.451	1264.791
OTX2	9760.037	11425.74	15397.9	13566.91
PAX6	4238.885	4534.985	5676.668	5663.953
RDH10	12254.24	16236.07	13442.79	13957.67
RDH5	2179.721	3529.103	4543.91	4348.016
RLBP1	18844.51	26422.1	18128.59	13225.5
RPE65	824.1694	662.6874	617.7234	611.5039
SERPINF1	104170.1	143150.8	160580.6	166325.6
SOX9	3614.678	4575.614	9578.108	10179.09
TJP1	1877.223	1761.045	1681.118	1766.934
TRPM1	2296.719	2572.621	4933.935	4740.985
TYR	6750.241	8084.94	11446.05	9078.06

Supplementary Table S2: AMD-related genes

Gene	POS (4Hrs)	POS (6Hrs)	POS (24Hrs)	POS (48Hrs)
ABCA1	520.9536	598.682	214.9797	205.6193
ABHD2	23000.79	25510.92	14166.49	9727.145
ABO	2.488484	0.700983	0	0
ACAD10	1750.777	1715.159	2203.592	1883.738
ACKR3	208.8793	233.3556	336.4548	376.0866
ADAMTS9-AS2	2.742215	6.226256	6.732832	6.737151
ALDH1A2	0	0.715855	0.250782	1.254055
APOC1	160.1098	93.48848	148.3653	140.8245
APOC1P1	1.381518	2.189718	0	0
APOE	14783.61	21386.17	23131.5	24949.28
ARHGAP21	5108.257	4074.705	5007.93	5024.229
ARMS2	2.771751	1.258049	2.76021	1.004365
ATF7IP2	185.1043	180.5804	207.543	211.2886
ATP6V0D1	7081.277	7263.002	7106.662	6625.999
ATPSCKMT	428.2938	519.2471	539.6546	505.865
B3GLCT	2011.469	1972.92	1977.758	1852.163
BAG6	6408.107	7162.851	8472.846	7431.858
BARD1	176.8287	169.6977	252.6365	273.1724
BBS9	309.2004	239.4255	684.5191	758.088
BRAP	1647.842	1640.588	1182.741	1186.133
C12orf50	7.697941	15.09802	12.56628	7.441769
C1orf53	87.84273	115.9446	147.0481	149.1195
C2	15.33036	19.70204	22.99888	20.82372
C3	3262.541	5980.02	4303.364	5069.685
C9	1.00066	0.49963	0	0
CCHCR1	164.7231	154.6732	273.7242	341.5829
CDH13	52.4567	16.52354	51.41398	43.27744
CDH22	0	0	0	0
CEP97	498.1458	496.3364	550.5074	582.716
CETP	0	0.479754	0	0.721686
CFB	104.5269	126.6613	161.2515	149.2517
CFH	1921.287	2393.847	2279.138	2699.554
CFHR1	35.08045	25.83191	116.7612	108.7602
CFHR4	2.441307	0.289795	1.984548	0.849149
CFHR5	0	0.25073	0.749705	1.745165
CFI	1198.064	1887.314	1907.342	2570.408
CLIC5	6.577379	2.445531	4.255255	3.407994
CLMP	840.974	824.8237	138.1058	160.5935
CLUL1	199.3005	262.6206	295.9154	344.7159
CNN2	11812.59	10040.46	11387.65	9943.448
COL10A1	0.499536	4.464196	2.249446	3.755534
COL4A3	1015.227	1135.714	1338.31	1219.638
COL8A1	29063.6	32347.53	48139.08	58927.85

COPS8-DT	27.61597	47.55562	89.71869	99.12005
CPN1	0	0	0	0
CTRB2	0	1.287029	0	0
CYP4B1	0	0	0	0
DAPK3	4571.809	4935.057	3554.71	3043.687
DENND1B	1037.021	813.345	1022.697	849.903
DPF3	36.48018	23.48587	31.26354	37.26285
DPP9	5174.344	5478.302	4086.224	3164.26
EFCAB14	7062.161	7623.49	7281.68	6460.004
EFNA4-EFNA3	109.1458	37.20643	72.65369	97.24946
ERICH1	1618.788	1531.384	874.5458	810.7614
EXOC3L2	3.741439	1.245371	0.745822	2.743183
FAM124B	7.502648	9.921081	4.797894	3.974241
FAM135B	0	0	0	0
FGD6	81.32671	123.7822	157.5272	181.6921
FRK	79.54934	115.2482	287.6103	341.2396
FUT6	0	0	0.972045	0.516971
GALR1	0	6.782336	0.793706	0.398119
GLI3	165.7634	156.2691	383.4447	291.5532
GRM5	4.969506	1.490691	8.713673	15.67648
HERC2	1145.052	1118.974	1115.887	868.4448
HERPUD1	4292.851	4269.254	6324.654	6597.141
HOMEZ	1148.616	924.4087	933.4305	960.3497
HS3ST1	201.8134	190.9264	271.4782	303.6326
HS3ST4	2.738848	1.745406	3.232219	7.729267
HTRA1	3982.994	4450.553	6349.79	6969.514
IFNAR2	426.6425	345.8729	426.1943	428.341
KAT7	1877.375	2082.03	2219.215	2101.848
KCNN3	9.19344	2.036743	3.009011	6.033212
KCNT2	272.7036	245.6778	223.3315	368.7907
KCTD14	142.3334	107.9534	147.7873	170.6239
KIAA0513	1092.396	1109.165	1083.827	972.9697
LINC00243	0	0.250249	0	1.248262
LINC00547	3.735799	0.250279	0.511185	0.24995
LINC00861	0	0	0	0
LINC01048	0	0	0	0
LINC01101	0	0	0	0
LINC01512	0	1.250668	0	0.500338
LINC01579	0	0	0	0
LINC01581	0	0	0	0
LINC01645	0	0	0	0
LINC01741	0	0	0	0
LINC01742	0	0	0	0
LINC01776	0	0	0	0.249191
LINC01823	0	0	0	0
LINC01826	0	0	0	0
LINC01916	0	0	0	0
LINC01924	0	0	0	0

LINC02112	0	0	0	0
LINC02360	0.745562	0	0	0
LINC02537	0	0	0	0
LINC02652	0	0	0	0
LINC02703	0	0	0	0
LINC02836	0	0	0	0
LINC03040	944.3824	968.5102	2381.964	1548.038
LIPC	12.20801	16.21291	17.71903	42.9311
LYAR	799.77	619.3927	303.3515	270.2846
MARK4	2950.026	2697.266	2170.726	2326.749
MBL2	0	0	0	0
MBP	8208.157	8945.817	9478.397	8710.925
MCHR2	2.268741	0.754568	4.030117	2.015333
MCUB	608.9369	552.9251	768.9481	679.1487
ME3	4618.037	5654.583	4391.404	3366.235
MFF-DT	49.89828	25.45099	39.4	25.45665
MIR29B2CHG	155.277	154.7506	175.2016	165.3783
MMP9	0	0	0	0.249162
NDUFC2-KCTD14	597.3354	612.972	898.1221	715.6965
NECTIN2	7981.707	8632.734	6329.566	5816.008
NELFE	3079.052	3203.599	2474.779	2289.196
NLRP5	0	0	0	0
NMRK2	21.09896	19.33238	28.09991	40.4318
NOTCH4	66.47518	75.43658	77.80312	65.55067
NPLOC4	9135.516	9543.676	9703.509	9523.56
NT5DC1	1681.645	1747.299	3759.439	3447.967
OAS1	9.218325	9.743538	10.27848	14.67403
OR5K3	0	0	0	0
PBX2	1088.797	1252.79	1395.692	1307.949
PCDH9	62.41014	35.52357	109.0005	151.5407
PDCL3P4	12.02237	7.471628	16.51637	19.51799
PDGFB	576.6286	549.361	156.4284	122.8992
PILRA	31.35639	19.15453	15.95652	11.95192
PKNOX2	19.82683	16.90808	96.52695	74.72624
PLA2G12A	2241.071	2061.284	2437.194	2652.316
PLCL1	37.83128	15.82116	117.5003	251.0867
PLEKHA1	2254.312	2152.555	1971.97	1876.007
PSORS1C1	5.272015	3.998488	5.50809	8.025627
RAB30-DT	91.67707	93.0002	83.70821	68.84663
RAD51B	148.8338	69.75295	262.4341	263.6332
RDH5	2179.721	3529.103	4543.91	4348.016
REST	1932.668	1762.43	1956.683	1826.012
RLBP1	18844.51	26422.1	18128.59	13225.5
RNF126P1	0	0	0	0
RORB-AS1	0	0	0	0
RPL3	112041.9	89278.78	110252.1	112268.4
RREB1	905.8751	939.9997	909.782	934.4425
SCIRT	102.044	113.8019	177.0724	188.1678

SCPEP1	2076.21	2362.723	2680.882	3421.841
SDK1	1513.285	1491.767	1598.484	2188.506
SKIC2	1662.97	1911.994	2158.46	1941.451
SLC16A8	41.94593	62.72779	138.0018	145.424
SLC44A4	0	0	0	1.066055
SLC6A20	4348.865	4827.39	5677.376	3183.641
SLC8A1-AS1	0	3.229712	0.248975	2.753518
SNHG14	1278.015	1147.032	2258.596	1721.88
SNX7	767.4973	727.1062	1178.689	1238.6
SOD2	32605.64	45719.16	22082.38	15892.61
SPEF2	305.9364	303.369	447.1441	500.3203
SRPK2	1639.893	1812.814	2408.203	2292.826
STK19	646.6912	807.234	644.3094	697.0924
STOX2	224.928	164.0499	356.2169	338.9666
SYN3	53.63119	65.49762	55.35329	35.77265
TACC2	1419.072	1280.222	1395.491	1418.289
TDRP	1713.612	1357.826	1139.02	1245.699
TEX15	0	0	0	0
TGFBR1	2492.872	1963.119	1229.884	1121.031
THUMPD2	360.6625	375.8708	298.4917	267.4583
TMEM97	1980.71	1060.642	1434.228	1200.866
TNFRSF10A-DT	70.82287	53.71669	60.8847	62.95818
TNR	0	0	0	0
TNXB	100.5035	137.1717	79.32272	31.23421
TOMM40	6677.843	7177.458	4005.955	3278.94
TRAPPC9	1593.503	1419.645	2385.338	2326.687
TRPM1	2296.719	2572.621	4933.935	4740.985
TRPM3	5992.836	7259.084	10109.1	13846.98
TSPAN11	21.2024	11.13883	16.7004	5.488876
TYK2	3061.188	3197.685	3856.943	3561.957
TYR	6750.241	8084.94	11446.05	9078.06
VSTM2A-OT1	0	0	0	0
VSTM2B-DT	0	0	0	0
WAC-AS1	637.5952	628.6844	895.1331	840.8
ZFP1	409.7816	324.0552	419.3835	453.3685

Supplementary Table S3: Inherited retinopathy genes in RPE cells

Gene	POS (4Hrs)	POS (6Hrs)	POS (24Hrs)	POS (48Hrs)
ABCA4	12.99136	16.20317	17.78827	20.8956
AIPL1	1.250871	1.559054	1.754181	0.498798
BBS1	2922.705	3289.825	4058.57	4062.326
BBS10	992.6096	1095.142	934.1575	895.8881
BBS12	366.1755	405.0791	500.773	547.9376
BBS2	1318.175	1239.212	2836.253	2634.891
BBS4	948.5795	1012.614	1419.158	1274.403
BBS5	1151.127	1366.665	1005.408	1057.357
BBS7	925.4635	838.7941	923.6365	872.5874
BBS9	309.2004	239.4255	684.5191	758.088
BEST1	879.0493	873.5358	893.9675	1204.543
CHM	1909.373	1821.966	1847.133	1572.435
CRB1	0	4.616117	9.317808	7.439869
ELOVL4	336.3528	308.692	346.0344	352.7579
LRAT	9.735207	10.74171	30.47774	64.08077
OAT	4283.779	3955.019	5479.029	4996.873
PRPH2	0.646585	1.579981	4.093143	0.720087
RPE65	824.1694	662.6874	617.7234	611.5039

Supplementary Table S4: Cell cycle-related genes

Gene	POS (4Hrs)	POS (6Hrs)	POS (24Hrs)	POS (48Hrs)
ABL1	6573.126	7048.464	8744.679	8299.779
ANAPC1	3152.732	3055.282	2610.869	2327.015
ANAPC10	270.3554	236.7486	223.3005	229.6377
ANAPC11	1825.673	1910.49	2179.069	2398.589
ANAPC13	1886.8	1745.623	2131.449	2011.133
ANAPC2	2498.51	2823.101	2969.462	2821.465
ANAPC4	618.5516	588.4099	690.6889	567.9803
ANAPC5	4321.123	4391.664	4931.941	4607.508
ANAPC7	2986.684	2964.947	2515.692	2192.328
ATM	1340.59	1445.451	2572.816	1960.282
ATR	1493.468	1723.179	1349.014	1091.131
BUB1	143.5264	72.78741	116.4165	172.6632
BUB1B	133.365	79.43495	129.685	147.8022
BUB3	3511.011	3667.005	3661.508	3023.306
CCNA1	13.01791	15.79798	0.265498	1.926882
CCNA2	537.5712	477.4212	1235.117	1267.942
CCNB1	473.6266	339.6049	347.9498	422.6276
CCNB2	115.302	73.60294	76.453	95.3735
CCNB3	12.39637	14.70748	13.60547	26.05977
CCND1	28733.24	35760	22017.07	18426.02
CCND2	0	0.248406	0	2.245493
CCND3	868.2748	844.8799	1158.558	1146.916
CCNE1	169.8489	94.98325	119.8424	161.3347
CCNE2	16.33223	12.26028	27.45576	13.87391
CCNH	2402.697	2367.357	1610.942	1594.023
CDC14A	154.1333	191.2115	192.8659	175.1005
CDC14B	1392.324	1436.906	1836.204	2137.811
CDC16	3547.379	3508.378	2903.547	2813.141
CDC20	142.7798	77.6424	88.68437	151.483
CDC23	886.2802	895.6298	939.735	880.5855
CDC25A	170.9173	99.35461	88.73347	93.17172
CDC25B	843.0789	747.8639	648.6982	676.4392
CDC25C	29.99246	14.25754	13.56292	20.70015
CDC26	596.8629	486.1391	645.7175	614.6495
CDC27	2889.592	2693.025	2549.823	2173.945
CDC45	58.42299	34.03773	67.4508	55.85139
CDC6	260.3973	178.9374	165.7507	158.7888
CDC7	195.1723	239.4778	324.1801	212.4322
CDK1	140.8498	91.19607	107.9076	186.3366
CDK2	443.4659	330.2956	653.9786	648.0276
CDK4	6690.921	6298.412	5553.952	5170.1
CDK6	2772.867	2158.531	3573.833	3494.724
CDK7	1259.598	1087.628	1104.718	959.4102
CDKN1A	26838.42	30528.11	10962.33	11960.96

CDKN1B	2441.909	2700.023	4421.039	5251.024
CDKN1C	250.7525	189.8535	549.7717	775.6195
CDKN2A	265.475	261.6884	355.8136	440.3935
CDKN2B	8112.953	7545.945	6351.323	5983.831
CDKN2C	62.19169	60.56499	151.8649	298.6304
CDKN2D	77.75892	53.2629	174.4729	207.6518
CHEK1	131.9948	107.0917	222.6387	208.5555
CHEK2	131.8795	110.9118	139.8197	123.2773
CREBBP	4869.958	5059.413	4639.244	4640.218
CUL1	3872.703	3548.411	3078.604	3124.702
DBF4	286.2498	247.7576	244.3468	210.8277
E2F1	207.7252	122.3619	226.1923	226.1273
E2F2	16.31644	5.37482	19.41467	37.31182
E2F3	1869.555	1778.173	816.7298	675.5296
E2F4	3452.05	3556.66	2134.747	1816.652
E2F5	843.0604	653.1945	571.4421	489.1202
EP300	4578.077	4819.96	4508.099	4050.065
ESPL1	160.4408	149.4999	246.6462	328.8593
FZR1	1835.469	1822.389	2435.586	2239.23
GADD45A	8214.085	12127.7	3449.767	2611.611
GADD45B	2135.546	2806.767	2225.548	2236.241
GADD45G	33.8792	33.10739	57.09722	59.52497
GSK3B	3226.904	3178.075	3238.616	3070.824
HDAC1	1631.752	1583.693	2058.202	2187.8
HDAC2	4847.259	4091.426	3526.361	3390.806
MAD1L1	794.0006	1070.795	1134.489	971.0178
MAD2L1	142.7042	128.511	195.9694	227.5428
MAD2L2	980.6709	990.1463	1018.184	1077.53
MCM2	1373.702	1178.127	1830.737	1431.512
MCM3	2167.984	2231.274	2551.585	2106.313
MCM4	1776.818	1538.088	1942.902	1571.674
MCM5	656.0771	579.979	1234.697	900.4049
MCM6	1204.285	1107.566	1615.404	1235.801
MCM7	4632.956	4216.248	3160.501	3146.325
MDM2	6131.15	7929.096	4418.336	3887.083
MYC	13991.13	13707.25	6173.96	4611.368
ORC1	30.39305	20.4739	44.2671	37.20601
ORC2	1206.269	1137.881	948.865	754.7785
ORC3	985.0237	1041.019	1279.736	1175.394
ORC4	1293.423	1210.453	1384.17	1392.492
ORC5	707.4978	740.5498	587.215	566.4849
ORC6	89.38055	68.92909	55.84073	57.1144
PCNA	1740.336	1695.709	2307.3	2158.952
PKMYT1	116.1897	85.59084	73.51335	102.6015
PLK1	194.1921	130.8808	70.93438	175.0515
PRKDC	6634.41	6706.105	5699.538	4779.038
PTTG1	144.0083	124.3991	98.58242	117.8564
PTTG2	0.510613	0.25113	0	0

RAD21	5253.38	5321.149	6207.93	6098.123
RB1	2597.953	2579.599	2958.522	2833.481
RBL1	291.8175	274.1075	390.554	374.5151
RBL2	2798.067	3152.65	3978.54	3911.178
RBX1	1376.338	1334.821	1518.405	1367.336
SFN	22.73651	12.48787	8.511292	3.000598
SKP1	11591.06	11998.02	14323.09	12581.26
SKP2	368.917	381.6624	634.7466	567.4123
SMAD2	83551.66	66550.47	91285.61	93410.94
SMAD3	5945.306	8087.332	8642.847	9078.063
SMAD4	4161.393	3625.87	4002.29	3403.033
SMC1A	3821.338	3567.824	3433.417	3051.845
SMC1B	1.475822	4.93238	0.736098	1.516272
SMC3	2356.905	2262.847	2534.931	2566.048
STAG1	2588.948	2687.867	2928.933	2612.175
STAG2	3150.569	3053.681	4575.582	4388.753
TFDP1	4281.717	4204.177	3530.106	3570.246
TFDP2	2008.183	2031.539	3233.948	2903.912
TGFB1	9145.858	10600.18	7276.556	7402.3
TGFB2	6338.543	3459.681	4359.153	3538.832
TGFB3	100.9495	111.5809	679.0063	772.964
TP53	4860.46	4926.903	2792.235	2416.413
TTK	62.89742	30.89935	43.51995	53.37612
WEE1	3739.656	2886.023	2604.688	1912.487
WEE2	0.248946	1.000898	0	1.249046
YWHAB	13237.14	13824.62	13708.4	11205.11
YWHAE	17365.3	16680.79	17222.53	15612.1
YWHAG	13623.81	11837.81	9517.425	9156.69
YWHAH	3632.332	2983.98	3203.851	2839.299
YWHAQ	15126.82	15297.8	14279.55	13060.67
YWHAZ	27710.35	21345.68	23355.14	22510.98
ZBTB17	892.1642	983.7904	1141.419	1171.272

Supplementary Table S5: Autophagy-lysosomal pathway genes

Gene	POS (4Hrs)	POS (6Hrs)	POS (24Hrs)	POS (48Hrs)
ABHD5	1330.871	1188.779	1057.994	1054.925
ABHD6	745.2683	733.6164	1022.392	934.8137
ACBD5	1451.014	1656.499	2914.72	3086.089
ACIN1	8274.9	8858.288	7396.339	6947.13
ACP2	2304.568	2719.698	3500.509	3380.149
ACP3	35.95216	36.93172	103.5905	97.85004
ACP4	0.751286	5.745226	0	0.748349
ACP5	209.2407	294.016	325.7138	315.6572
ADRB2	256.7054	306.8468	152.246	164.2561
AGA	1918.176	2011.25	2723.653	3028.035
AKT1	8092.115	8345.696	5818.1	5950.722
AKT1S1	4109.939	4693.276	4846.351	4420.364
AMBRA1	2947.726	3318.377	2976.631	2683.903
ARFIP2	3217.205	3325.849	2738.333	2627.882
ARIH1	3307.762	2949.171	3390.246	3189.492
ARL8A	3247.974	3070.005	2526.772	2609.257
ARL8B	5166.035	4636.475	4294.577	4112.529
ARSA	2968.208	3706.342	4838.536	4415.809
ARSB	2492.212	2626.337	3069.51	2874.705
ARSD	3036.301	3258.891	3593.873	3531.017
ARSG	910.2806	949.8894	1883.666	1391.645
ASAH1	19229.36	22201.25	29300.72	31109.68
ATAD3A	3672.691	3501.279	1798.022	1614.389
ATF4	26102.14	25247.02	8625.732	10732.82
ATF5	1494.521	1426.742	803.3063	737.812
ATF6	4640.39	4967.294	4709.253	3787.912
ATG10	227.0153	250.9988	518.4092	461.9802
ATG101	2530.567	2360.175	1363.703	1377.721
ATG12	1137.742	1435.15	1284.665	1157.634
ATG13	6188.594	6755.63	6674.322	6587.592
ATG14	2873.331	3119.068	2803.001	2731.015
ATG16L1	1829.968	1618.044	1153.988	1160.103
ATG16L2	440.7218	482.0256	652.8565	760.9038
ATG2A	6491.076	8354.905	4902.5	4036.779
ATG2B	1358.906	1470.282	1726.648	1465.714
ATG3	2064.671	2130.567	2041.382	1908.842
ATG4A	439.902	423.6063	778.4778	764.2047
ATG4B	3280.095	3467.825	2510.346	2431.17
ATG4C	233.7284	247.4237	519.7897	493.8
ATG4D	1086.234	1267.043	1397.63	1514.332
ATG5	1497.102	1465.722	1315.684	1178.678
ATG7	1673.771	1747.065	1724.343	1502.176
ATG9A	3483.256	3628.111	3972.2	3828.665
ATG9B	13.94276	3.979485	6.885199	3.022232

ATL2	1266.957	1106.409	1374.099	1296.816
ATL3	4934.182	4727.126	4482.131	4214.766
ATM	1340.59	1445.451	2572.816	1960.282
ATP13A2	4534.679	4971.409	6289.277	5983.886
ATP5IF1	2057.668	1742.822	2661.52	2364.263
ATP6AP1	10068.5	10258.36	13051.86	13806.61
ATP6AP2	5585.792	5644.952	7099.768	6956.607
ATP6V0A1	5152.771	5273.937	5253.92	4286.82
ATP6V0A2	2971.491	2811.477	1245.371	1117.922
ATP6V0A4	26.08962	22.55889	28.63157	14.5552
ATP6V0B	5971.724	5367.497	5262.565	4873.721
ATP6V0C	14827.03	16777.37	15318.53	14414.45
ATP6V0D1	7081.277	7263.002	7106.662	6625.999
ATP6V0D2	0.500804	0.726097	0	0
ATP6V0E1	7003.017	7491.792	9011.375	8193.891
ATP6V0E2	3783.79	3913.953	6982.697	7554.592
ATP6V1A	5965.147	5673.92	9035.005	8068.679
ATP6V1B1	56.62972	55.76157	26.76606	26.50844
ATP6V1B2	8348.52	8566.87	10148.72	8073.047
ATP6V1C1	4696.983	4644.275	4691.128	4075.919
ATP6V1C2	3988.101	5675.41	3604.29	1814.375
ATP6V1D	2904.994	2855.991	3757.673	3661.089
ATP6V1E1	6522.341	6830.64	7421.911	7129.502
ATP6V1E2	167.2124	146.8915	281.4759	276.8058
ATP6V1F	4570.742	4564.191	4932.293	4708.794
ATP6V1G1	6646.196	5802.725	5202.289	4598.005
ATP6V1G2	30.48693	15.14287	48.07498	50.11811
ATP6V1G3	1.817437	2.45264	1.846714	1.803498
ATP6V1H	4840.626	5864.807	4187.417	3551.464
ATXN3	711.8619	610.4339	814.2292	764.466
AUP1	5333.068	5295.691	4455.035	4011.471
AURKA	189.5502	129.1964	186.3775	191.5414
AZI2	1674.285	1466.572	1764.123	1986.849
BAD	1917.647	1842.329	2466.771	2587.661
BAG3	3370.14	3061.08	2380.384	2252.977
BCAS3	549.3158	513.4773	1144.019	1098.457
BCL2	1498.119	1488.869	900.5922	844.2846
BCL2L11	905.7711	828.3723	1207.973	1660.449
BCL2L13	5897.755	6172.907	8808.336	8774.702
BCLAF1	6739.749	6862.598	6029.364	5011.128
BECN1	3015.148	3207.435	3117.171	3051.221
BECN2	0	0	0	0
BIRC2	1112.487	1132.351	1024.426	1060.562
BIRC5	191.7321	88.28243	101.8992	194.9326
BIRC6	2809.227	2702.616	2489.723	2148.235
BLOC1S1	1013.585	1078.168	1123.78	1207.025
BLOC1S2	2057.214	2112.426	1415.486	1385.093
BMAL1	1373.894	1749.193	514.4172	640.3485

BMF	2916.733	2743.362	5498.905	8815.436
BMT2	532.7038	481.258	736.6015	798.8805
BNIP1	248.7233	282.4692	289.8553	293.8692
BNIP3	2454.874	2406.995	3290.676	3736.558
BNIP3L	5888.824	5548.701	9265.132	8876.245
BORCS5	702.0026	775.3969	1071.957	1100.75
BORCS6	346.2017	287.7445	661.4984	637.2324
BORCS7	1379.426	1365.22	1438.32	1329.3
BORCS8	713.3056	848.4672	722.2866	721.2264
BRSK1	807.8814	809.9183	803.4866	908.2753
BRSK2	649.5338	684.4962	609.872	688.6028
BSG	72793.95	74304.81	91566.86	91475.51
C1QBP	6725.466	6996.72	6769.811	5914.059
C9orf72	312.7985	329.335	310.3105	331.1867
CAB39	4492.152	4108.391	3649.308	3538.207
CALCOCO1	2986.329	3742.077	5503.566	5171.887
CALCOCO2	9272.792	10500.44	10263.26	10024.94
CALM1	8808.724	9012.268	11944.05	11278.71
CAMK1	1467.653	1401.652	1383.531	1292.69
CAMKK2	3664.454	3131.966	2044.183	1909.11
CAPZB	6190.425	6473.425	6475.457	5629.519
CASTOR1	319.2739	353.8862	376.0696	405.4272
CASTOR2	691.0212	804.4491	1067.946	1066.069
CCDC88A	801.4321	759.3866	990.1715	885.8483
CCL2	917.2417	2067.774	2164.175	2380.895
CCNB1	473.6266	339.6049	347.9498	422.6276
CCNE1	169.8489	94.98325	119.8424	161.3347
CCPG1	8657.792	7690.847	7298.218	7451.525
CCZ1	1462.721	1392.473	1437.981	1432.554
CCZ1B	1369.17	1455.961	1420.081	1305.936
CD84	0.247927	1.876224	3.488958	0
CDK1	140.8498	91.19607	107.9076	186.3366
CDK5	666.0435	620.652	1161.082	1155.418
CDK5R1	198.1127	153.3895	277.4916	265.8047
CDK5RAP3	5648.75	5944.391	6039.642	5735.219
CDKN1B	2441.909	2700.023	4421.039	5251.024
CEBPB	10625.04	12458.8	6462.669	7727.064
CFLAR	2952.914	2588.482	4740.736	4802.33
CHIT1	0	0	0	0.248966
CHMP1A	5358.037	5528.705	4998.136	4425.484
CHMP1B	4171.933	4241.177	3480.632	3509.307
CHMP2A	2965.76	3166.956	3934.402	3336.929
CHMP2B	2406.915	2491.68	2523.448	2238.245
CHMP3	4591.403	4267.196	4268.984	4031.401
CHMP4A	1441.68	1472.837	1333.771	1238.22
CHMP4B	5713.669	6787.453	4614.154	4198.23
CHMP4C	350.7576	373.7262	532.0698	517.7925
CHMP5	2248.134	2339.59	2509.432	2414.818

CHMP6	1849.63	1968.836	1485.449	1356.105
CHMP7	2492.489	2362.389	2251.708	2206.025
CHUK	2245.27	2024.638	1788.941	1707.264
CIP2A	165.0206	160.108	163.4493	121.5818
CISD2	1534.997	1478.765	2196.462	2049.525
CLCN7	14484.05	16234.31	14345.99	12329.63
CLEC16A	2286.698	3110.507	2681.049	2186.459
CLN3	3158.171	3253.185	4299.266	3807.371
CLU	90034.06	137947.3	152940.3	162298.5
CPTP	1903.682	2029.878	1902.274	1964.661
CSNK1A1	9370.277	9258.213	7576.351	7331.79
CSNK1D	7973.874	8058.601	6825.333	6530.148
CSNK2A1	5899.25	5577.901	5313.363	4748.119
CSNK2A2	3810.244	3181.823	2784.362	3351.853
CSNK2B	4530.498	4616.499	4367.391	4354.806
CTBS	1582.868	1531.908	1913.534	2138.104
CTNNB1	9924.341	9257.407	11332.94	9557.461
CTSA	15574.51	17661.13	25258.34	25103.54
CTSB	34899.67	40199.36	47265.63	46370.73
CTSC	1258.455	1237.189	1832.934	1579.951
CTSD	310790.8	376281.7	503255.6	494853
CTSF	17998.97	19787.85	21087.82	22719.74
CTSG	0	0	0	0
CTSH	17769.91	17397.08	21540.43	26594.42
CTSK	13724.38	18449.99	25249.84	28044.12
CTSL	5807.888	5551.315	7787.864	7248.196
CTSO	1233.224	1527.81	1856.174	1706.888
CTSS	23.47447	28.91407	29.56352	27.94709
CTSV	2749.523	3080.25	3325.378	4571.069
CTSW	2.760292	1.754866	1.253609	0.249916
CTSZ	6175.717	6831.293	7868.4	8013.761
CTTN	18936.18	20337.86	14213.7	11920.5
CUL1	3872.703	3548.411	3078.604	3124.702
CUL3	4163.738	3436.829	3228.977	3126.392
CUL4A	7556.294	7281.068	4787.916	4383.797
CUL4B	3098.363	3128.581	4227.236	4026.963
DAGLB	1161.977	1269.918	1065.953	943.9413
DAP	11009.68	11539.6	13007.32	11703.12
DAPK1	1239.063	1507.692	1224.304	1198.497
DAPK2	155.6288	212.0496	198.9786	234.8181
DAPK3	4571.809	4935.057	3554.71	3043.687
DAXX	2613.193	2188.076	2153.887	1985.464
DCAF12	3286.589	2843.38	2409.088	2353.132
DCN	8.878564	7.061816	3.110286	9.675658
DDB1	14602.67	15160.07	17256.04	14644.51
DDIT3	4519.358	4794.042	1442.876	1870.893
DDIT4	7942.245	7409.441	6321.616	7383.828
DDRGK1	2463.25	2626.62	2556.776	2454.062

DDX5	22188.66	24263.95	20041.78	17621.68
DELE1	3900.422	4607.213	5089.818	4703.993
DEPDC5	595.0132	722.2957	841.9463	817.4565
DEPTOR	29.52224	20.83482	60.68309	58.00403
DNAJB1	7247.679	5636.149	5681.728	5513.025
DNASE2	4541.679	4438.208	6559.965	6532.461
DNASE2B	0	0	0	0
DNM1L	3294.603	3428.072	2703.412	2481.235
DNM2	5749.522	5737.735	7814.163	7215.494
DPP4	68.37647	64.89651	61.96399	62.96017
DPP7	16551.93	19649.94	21778.24	20840.62
DRAM1	15686.26	19589.99	8553.938	5768.427
DRAM2	1157.669	925.2549	1475.351	1597.71
DRD2	8.071311	8.341116	7.073894	2.270998
DRD3	0	0	0	0
E2F1	207.7252	122.3619	226.1923	226.1273
EEF1A1	696529.5	611117.2	703618.4	710491.6
EEF1A2	1.24834	0.249935	14.00779	0.999675
EGFR	12485.26	10801.41	13816.98	12022.33
EHMT2	2192.697	2002.628	3503.111	3282.57
EI24	6300.02	6379.791	5733.304	5148.185
EIF2AK1	5716.091	5537.435	7413.607	7131.326
EIF2AK2	2232.493	2012.856	2432.145	2229.629
EIF2AK3	2016.237	1594.862	1663.332	1541.413
EIF2AK4	2039.111	2037.207	2380.711	2416.402
EIF2S1	4093.54	3486.955	2129.793	1925.031
EIF4G1	27911.75	28419.49	19971.55	17322.65
ELAPOR1	72.04302	71.00196	60.49718	32.93424
ELAVL1	4059.701	4124.334	3986.592	3786.819
EMC6	1486.178	1488.899	1235.115	1227.99
EP300	4578.077	4819.96	4508.099	4050.065
EPG5	2724.13	2855.438	2505.4	2361.09
ERBB2	4386.464	4847.747	5501.248	4650.929
ERN1	706.5068	796.7532	485.9544	481.9902
EVA1A	2579.063	3247.358	972.8516	756.792
EXOC1	2000.448	1991.526	2027.761	1909.889
EXOC1L	0	0	0	0
EXOC2	1789.534	1854.497	1968.163	1758.402
EXOC3	3573.02	3931.375	4152.044	3927.172
EXOC4	2705.106	2175.348	4921.577	4569.948
EXOC5	3020.63	2653.385	3009.881	2564.222
EXOC6	1165.158	898.5514	824.6354	680.09
EXOC6B	2878.189	3353.19	4008.298	3514.691
EXOC7	7891.47	8973.01	8460.002	7923.716
EXOC8	844.8316	946.2231	738.7807	701.6614
EZH2	392.9142	298.9946	256.6218	271.2174
FBXO7	5359.582	5257.219	5678.891	5694.657
FBXW5	7115.174	7605.483	7053.043	7014.44

FBXW7	798.7306	695.3246	486.6463	467.7161
FEZ1	61.67966	58.20756	195.5746	277.2473
FEZ2	2440.487	2398.729	3376.781	3749.375
FIG4	1227.55	1250.232	1535.629	1390.415
FKBP5	461.7213	296.7039	217.9577	171.3747
FKBP8	12567.93	12324.68	15046.16	15655.5
FLCN	2183.342	2544.675	2527.734	2463.07
FNBP1L	2726.921	1859.244	3332.261	3263.461
FNIP1	1710.126	2030.901	2267.076	1921.83
FOS	98.76366	171.4143	297.4805	138.535
FOSB	80.83639	163.0838	30.13554	15.82401
FOSL1	1658.808	3038.603	220.9562	165.301
FOSL2	3563.267	4037.085	3561.551	3468.584
FOXK1	4002.189	3472.834	3365.393	3177.477
FOXK2	5013.516	3987.916	3309.296	3528.437
FOXO1	787.4704	840.5347	944.2097	870.8151
FOXO3	5018.918	4226.6	4412.423	4163.163
FUCA1	1882.536	2004.997	3055.225	3081.953
FUCA2	6661.841	6847.133	8549.868	7541.232
FUNDC1	395.6067	412.9929	590.3375	565.4483
FUS	10042.86	10081.57	5852.592	5131.731
FYCO1	4106.135	3964.72	4030.958	3489.941
GAA	7632.101	7889.143	9943.207	11129.22
GABARAP	11479.41	10151.91	13570	14700.66
GABARAPL1	4389.501	4978.636	4081.087	4432.88
GABARAPL2	2878.99	2755.145	4021.922	4073.433
GALC	1784.192	1733.581	2676.506	2589.295
GALNS	1855.277	1885.248	2198.637	2254.221
GATA1	1.248672	0.999936	0	1.994608
GATA4	0	0	0	0
GCN1	7920.841	8185.716	6791.785	5813.292
GFAP	9.7807	2.329494	12.40065	5.605066
GGH	375.2485	333.211	480.0867	488.1744
GJA1	9917.898	13082.99	39635.49	47664.82
GLA	361.5297	317.2461	473.748	405.3468
GLB1	8878.102	8562.504	10496.79	10825.2
GLI1	8.102994	3.296773	12.2753	13.15517
GLUD1	6438.052	6280.133	6333.389	5646.521
GM2A	1948.795	2088.487	4317.451	3988.53
GNAI3	2970.42	2767.071	2635.807	2084.861
GNS	39831.21	42140.43	54191.77	60637.68
GOLGA2	3024.789	2971.751	3667.336	3549.816
GORASP2	5536.969	5519.986	5467.136	5257.464
GPLD1	115.9029	150.3196	210.8461	230.7856
GPR137B	2408.608	2956.342	2502.505	2300.349
GPSM1	3862.643	3546.201	1755.618	1896.017
GRAMD1A	5674.169	4674.427	4552.224	5149.468
GRN	22880.05	23227.34	31264.52	32324.17

GSK3A	4745.653	4625.172	3925.357	3849.44
GSK3B	3226.904	3178.075	3238.616	3070.824
GUSB	3478.099	3836.646	4580.071	4633.32
HAP1	3.422188	0.486549	0	0
HAX1	2746.596	2548.321	2031.955	2079.48
HDAC6	2339.083	2246.722	2858.143	2845.312
HERC1	1259.734	1257.644	2208.086	2006.666
HEXA	12761.65	13958.84	17503.85	18006.89
HEXB	9203.224	9805.574	12508.31	11659.6
HGSNAT	3932.48	3239.466	3396.59	3538.355
HIF1A	10405.99	11396.25	8536.65	10314.76
HK2	2516.1	2225.751	1267.288	1300.496
HMGB1	10176.22	10340.14	10573.85	9127.803
HMOX1	11292.46	5666.009	2183.565	2906.547
HOTAIR	0	0	0	0
HPSE	158.1176	97.0779	164.9443	124.878
HRAS	1821.645	1847.188	1235.435	1133.937
HS1BP3	1543.614	1751.163	2753.089	2493.026
HSP90AA1	56446.76	51539.81	47440.52	37673.46
HSP90AB1	59794.97	59324.43	54864.52	47704.46
HSPA1A	10768.61	9448.769	6583.007	7042.784
HSPA1B	11300.89	6277.058	5356.385	6157.636
HSPA8	71575.72	71334.57	57925.72	50703.72
HSPB8	13506.8	9977.2	14500.05	14852.44
HTR2B	64.92211	53.28002	124.1689	118.8788
HTRA2	1437.641	1510.206	1241.294	1254.779
HTT	4167.196	4192.358	4147.896	3556.881
HULC	0	0	0	0
HUNK	1.247735	5.006786	0.748659	4.737186
HUWE1	9002.564	8086.092	9109.357	8000.746
HYAL1	672.126	655.9769	1574.897	1571.761
HYAL2	3005.47	2663.316	2802.466	2892.838
HYAL3	345.9092	301.4448	471.1	475.3337
IDS	7790.165	8093.56	7074.755	6367.713
IDUA	3499.666	3946.885	3924.703	4015.01
IFI16	132.9009	163.5068	171.6163	167.7838
IFI30	1275.617	1375.771	1923.468	2063.42
IFNG	0	0	0	0
IFT20	606.2617	550.4946	684.1507	700.0741
IFT88	643.782	674.2519	837.9725	755.4354
IGBP1	2486.412	2126.196	2787.873	3569.917
IGF1	25.31633	48.62361	62.28818	139.4638
IGF1R	5465.785	5134.355	6360.826	5583.689
IGF2BP1	0.502831	3.494745	0.249062	1.752037
IGFBP3	3686.275	4892.222	4008.126	4441.777
IKBKB	2247.051	2511.274	1871.535	1798.213
IKBKG	1244.166	1490.643	970.6917	875.0615
IL10	3.293822	0.759645	0.759164	0.252235

IL10RA	1.008665	0.251919	1.007844	0
IL13	0	0.638858	1.011732	0
IL17A	0	0	0	0
IL17RB	228.3743	215.7486	339.1864	338.527
IL4	0	0	2.293292	0
IL6	23.42516	29.55049	25.28013	14.40149
IMMT	4617.466	4394.001	5118.652	4711.862
INPP5E	1724.954	1838.543	1410.707	1367.965
INS	0	0	0	0
IRF8	0	0	0	0
IRGM	0.970184	2.435434	2.090573	0.96842
IRS1	4056.244	5599.157	5655.114	4475.245
IRS2	8674.676	9980.816	6737.879	6565.114
IRS4	0.478054	0.5967	1.426745	3.652685
ITFG2	1002.122	1029.464	1010.782	918.1414
ITPR1	860.47	555.6074	1208.562	1151.199
JMY	1568.822	1374.145	1232.393	1284.72
JUN	9611.012	13803.39	6627.745	4658.249
KAT5	2116.134	2168.934	2189.317	1922.437
KAT8	1274.653	1407.91	1472.807	1562.183
KDM4A	2796.477	2704.229	2891.825	2841.373
KDR	40.36635	15.23329	17.46463	22.68125
KEAP1	5136.063	4650.983	5046.455	5396.198
KICS2	384.4693	444.2325	316.85	329.0837
KIF1B	4318.692	3159.003	4938.914	4275.022
KIF5B	10271.22	9023.429	6631.466	6438.929
KLHL20	586.5084	596.4139	651.5995	659.4544
KLHL22	1509.253	1599.135	2167.296	2126.837
KPTN	485.1759	498.8055	586.946	582.2853
KRAS	2355.089	1964.933	1564.995	1290.459
KXD1	2761.948	2498.656	2479.325	2249.812
LACRT	0	0	0	0
LAMP1	15678.63	14850.18	16287.46	16369.55
LAMP2	20254.95	21675.04	27123.39	26809.71
LAMTOR1	6099.388	6882.704	6108.026	5327.424
LAMTOR2	907.5611	827.7521	1202.151	1181.182
LAMTOR3	3073.318	2914.208	3065.719	2753.49
LAMTOR4	1956.031	1858.175	2434.079	2383.549
LAMTOR5	2631.993	2718.195	3059.675	2776.677
LAPTM4B	12261.69	10994.67	14989.44	11931.92
LARS1	7997.771	7701.192	6770.737	5889.147
LEP	0	0	0	0
LEPR	430.6507	428.7921	708.578	673.8045
LGALS8	2073.415	2247.03	2655.852	2563.695
LGMN	9014.084	8360.906	10733.63	11985.74
LIPA	3150.463	3219.453	4754.155	3460.56
LITAF	22844.2	20569.73	8986.357	8409.728
LMNB1	260.6869	123.5716	237.0412	299.9785

LPIN1	5811.841	4551.879	5688.978	5637.668
LRPPRC	9616.606	8924.252	10875.83	9450.908
LRRK1	226.5244	249.0991	177.3592	147.7285
LRRK2	149.0162	186.7288	411.6495	398.262
LRSAM1	1214.818	1483.39	2084.965	1841.023
MAGEA3	0	0	0	0
MAGEA6	0	0	0	0
MAN2B1	8748.087	8623.633	9828.804	10749.39
MAN2B2	5223.645	7069.574	8228.363	8273.985
MANBA	2689.588	2244.248	4435.471	4891.304
MAP1LC3A	787.5166	998.8059	595.054	757.3715
MAP1LC3B	15958.43	18632.29	5024.078	5409.32
MAP1LC3B2	7.874098	12.34256	6.933204	6.651027
MAP1LC3C	1.004427	2.932128	3.997837	1.749932
MAP1S	2974.983	2848.839	2482.614	2397.294
MAP2K1	3500.688	3299.747	2827.821	2711.051
MAP2K2	9952.044	9374.687	10841.37	10542.25
MAP3K7	2441.522	2193.339	1815.792	1744.998
MAPK1	7195.456	6769.523	7017.34	6308.224
MAPK14	2509.75	2406.559	3395.162	3056.188
MAPK15	16.40534	16.49615	9.238593	17.65419
MAPK3	2277.969	2164.405	3264.892	3674.773
MAPK8	1117.852	1154.344	949.7542	894.239
MAPK8IP1	2692.576	3103.817	2711.427	2740.562
MAPKAP1	5194.733	5328.014	5787.464	5580.298
MAPKAPK2	7237.148	7113.364	5146.837	4941.509
MAPKAPK3	6331.762	6760.074	5934.476	4779.053
MARCHF5	2643.716	2468.517	2637.706	2506.459
MARK2	2069.718	2102.954	1903.775	1971.146
MARK4	2950.026	2697.266	2170.726	2326.749
MCL1	7890.901	10285.45	7917.072	6753.238
MCOLN1	1503.349	1603.638	2791.903	2621.797
MCOLN3	1395.304	1581.708	1027.969	966.4265
MEFV	1.817785	1.562973	1.77412	1.222255
MFN1	1888.954	1747.839	1852.867	1631.93
MFN2	6779.787	6441.48	7105.588	6654.76
MID2	1049.284	875.8163	967.135	974.2978
MIOS	1125.106	1073.539	888.0902	804.1484
MIR101-1	0	0	0	0
MIR124-1	0	0	0	0
MIR155	0	0	0	0
MIR21	0	0	0	0
MIR30A	0	0	0	0
MIR33A	0	0	0	0
MIR34A	0	0	0	0
MLST8	4024.913	3957.583	5386.414	5451.742
MON1A	888.3482	816.3415	753.9848	698.5851
MON1B	3786.541	4333.268	3012.899	2639.257

MTDH	8646.508	8189.761	8570.882	7643.235
MTMR14	3198.051	3510.188	3996.885	3573.816
MTMR3	3326.001	2852.764	3575.491	3369.747
MTMR4	2392.977	2601.734	2720.995	2711.715
MTMR8	66.09241	59.69043	94.27125	117.252
MTMR9	1373.51	1270.181	875.088	938.1037
MTOR	4798.405	4990.974	4825.547	4335.018
MUL1	3038.565	3739.299	3213.422	3011.442
MVB12A	1770.805	1826.926	2554.217	2246.791
MVB12B	1174.641	1327.223	1069.668	1116.842
MYCBP2	1540.306	1358.132	1788.789	1509.378
MYO6	2300.365	2550.379	2393.293	2479.361
NAA10	1468.659	1514.052	1247.856	1126.901
NAAA	440.846	460.8991	560.8652	584.1218
NAGA	1624.525	1710.717	2801.883	2656.556
NAGLU	9440.89	11756.96	15023.64	13812.98
NAP1L1	26453.81	23283.75	21012.78	23683.33
NBR1	5433.718	5886.784	7703.061	6597.519
NCOA4	7904.714	7441.112	10590.23	9837.665
NCOR1	4448.99	4389.606	4436.145	4124.106
NEDD4	1309.468	1043.999	639.6153	613.0177
NEDD4L	5271.578	5634.344	8804.221	9743.839
NEU1	4800.85	4577.722	7845.6	7395.776
NEU4	0	0	0.509656	0
NFATC1	912.0824	899.5444	1063.413	1044.158
NFE2L2	7336.62	8271.734	6841.443	7166.456
NFKB1	1965.088	1705.816	1679.382	1769.792
NFKB2	2522.499	2521.727	1204.376	1040.12
NFKBIA	1104.336	1460.311	1435.162	1491.789
NFKBIB	1456.004	1892.634	1134.965	1071.308
NIPSNAP1	3466.421	2878.934	5622.658	5088.415
NIPSNAP2	4432.314	4185.953	6757.562	6426.659
NLRX1	1517.879	1784.173	2258.838	2324.602
NOD1	518.0159	481.9408	493.6799	434.3794
NOD2	1.243909	0	0.250299	0
NOX4	1397.577	1811.008	1937.812	1467.533
NPC1	9576.383	9183.181	14509.8	13187.64
NPRL2	422.22	426.2283	768.7419	706.6881
NPRL3	2260.898	2569.41	3088.146	2860.015
NR1H4	0	0	0	0
NRAS	3752.799	3335.326	2194.194	2089.084
NRBF2	965.3173	886.9499	802.8002	861.4604
NUFIP1	705.6078	619.0646	433.4513	374.9682
NUPR1	10035.11	11475.32	7651.708	9958.607
OGT	7063.571	6844.758	5542.551	5136.2
OPA1	3403.238	3461.938	3465.973	2808.852
OPTN	5723.327	5590.045	6606.518	6593.412
OSBPL1A	6451.469	6740.634	10870.97	9753.411

PACS2	4569.164	4791.219	4702.656	4596.246
PAQR3	1226.413	1025.036	1070.176	996.0131
PARK7	5740.107	5428.121	6990.051	5926.409
PARL	1536.687	1720.776	1180.937	1101.837
PCYOX1	4662.32	4107.023	7228.225	7438.664
PDCD6IP	7051.898	7769.408	8093.42	6456.5
PDPK1	4083.604	3828.36	3774.192	3753.238
PEA15	19506.65	20916.57	12826.28	10709.9
PEG3	1.266463	0.959834	1.784466	0.239474
PEX5	3778.347	3934.599	4707.08	4902.204
PGAM5	3326.28	2924.003	1760.677	1551.307
PGK1	16598.6	17641.74	24619.26	22044.47
PHAF1	969.018	821.7663	827.6246	822.9563
PHB2	8973.175	8377.966	8259.544	8106.611
PHF23	3206.174	2297.6	2020.819	2025.947
PHLPP1	2072.672	1800.374	1750.619	1692.886
PI4K2A	18942.44	18243.33	12942.99	12671.17
PI4KB	3315.258	3452.764	4248.656	4113.003
PICALM	8126.834	6871.926	5855.992	5453.418
PIK3C3	1862.886	2170.098	1958.554	1762.306
PIK3CA	1100.278	946.353	1035.482	989.5554
PIK3CB	1077.417	1136.359	1610.178	1622.597
PIK3CD	1066.122	942.1536	896.1871	874.884
PIK3R1	1327.968	1293.46	1452.539	1307.295
PIK3R2	12374.48	12423.5	18324.6	17288.73
PIK3R4	1373.544	1329.142	1439.51	1321.158
PIKFYVE	2343.11	2351.503	2224.062	2131.152
PINK1	5427.149	6232.288	7878.448	8283.357
PIP5K1B	3265.746	4140.797	6869.722	7504.94
PJVK	29.6687	52.76084	50.16843	51.86858
PLA2G10	5.192732	2.394558	0.91012	0.164719
PLA2G15	2426.922	2210.94	2566.038	2470.674
PLA2G4E	0	0.488546	0.485221	1.456394
PLA2G5	78.67937	143.9263	282.8171	346.13
PLAA	1487.21	1509.543	1485.215	1469.642
PLBD1	321.88	396.9102	552.0118	570.6138
PLBD2	6072.504	5907.055	5767.227	5673.639
PLD1	488.4974	495.4291	1993.716	1928.669
PLD3	10693.42	11678.43	14271.44	15731.34
PLEKHM1	4510.827	5296.028	3539.194	2899.404
PLEKHM2	12566.79	14456.73	10577.47	10351.94
PMAIP1	394.9996	409.2441	194.5941	218.0228
PML	1695.543	1660.481	2135.913	2159.403
PON2	1479.123	1467.473	2272.921	2198.672
PPARA	2503.156	2363.685	3795.136	3648.781
PPARGC1A	2906.683	3896.696	5050.279	4862.465
PPP1R13L	5034.835	4969.654	4621.301	3682.484
PPP2CA	7734.681	6944.777	5827.073	5350.288

PPP2R2A	2363.015	1742.536	1493.734	1416.203
PPT1	6805.239	6286.383	8981.464	7314.984
PPT2	494.6186	447.5015	685.8454	679.2342
PRDX6	7689.052	7318.671	8935.814	7934.848
PRKAA1	2618.583	2650.598	2429.23	2081.727
PRKAA2	1085.057	1048.711	1162.646	1167.227
PRKAB1	1713.46	1861.605	1234.304	1150.591
PRKAB2	917.2325	782.0199	976.3132	1020.996
PRKACA	3251.018	3062.097	4152.561	4023.876
PRKACB	2617.332	2561.151	2752.467	2773.439
PRKACG	1.750072	0	0	1.500154
PRKAG1	2593.03	2554.579	2754.009	2734.145
PRKAG2	1905.437	1698.09	809.0812	688.8189
PRKAG3	0.499389	1.496318	0.749252	0
PRKAR1A	12770.04	12907.71	14106.93	12937.7
PRKAR1B	1767.639	2161.828	1279.877	1201.742
PRKAR2A	3680.001	3400.667	3882.384	3406.895
PRKAR2B	800.6911	743.8872	1443.536	1174.994
PRKCD	1419.603	998.9467	1154.812	1764.859
PRKCI	2895.376	2407.879	3731.761	3892.944
PRKCQ	31.10725	35.77154	99.53064	124.9221
PRKD1	309.5604	309.3802	371.7214	346.4412
PRKN	140.7548	127.4877	288.5342	254.9943
PROM1	29.4731	32.96587	17.43479	21.70681
PRTN3	0.50074	0	2.009217	9.01645
PSME1	3473.701	3204.619	4353.935	4015.752
PTEN	2841.15	2688.609	3950.935	4268.295
PTK2	5390.395	5484.454	5189.808	4747.341
PTPN2	1096.814	983.7319	765.1037	676.7784
PTPN22	0	0	0	0.220179
PYGB	6279.415	6778.248	4822.955	4609.011
QSOX1	17381.17	18501.6	20604.12	20467.93
RAB11A	4748.748	4799.674	5701.042	4935.503
RAB12	1691.241	1646.563	1538.246	1531.991
RAB17	487.1335	501.6458	1456.003	1298.407
RAB1A	7157.181	7223.839	8291.989	7569.71
RAB1B	7344.767	8249.901	9270.952	8409.698
RAB21	4064.111	4174.849	2833.206	3083.234
RAB23	3910.556	2829.585	1611.353	1617.145
RAB24	1584.216	1541.395	1793.335	1767.723
RAB26	377.0304	338.5633	751.6606	1310.01
RAB29	2066.947	2034.884	3515.939	3286.932
RAB2A	5017.86	5070.726	5272.622	4829.929
RAB32	3778.417	3300.317	2934.892	2763.105
RAB33B	555.9138	513.467	650.195	704.7132
RAB39A	1.750774	0.249026	1.74793	0
RAB39B	1048.461	1107.337	521.8144	534.6395
RAB4A	2044.317	2053.027	2950.5	3056.053

RAB4B	408.3124	465.4154	577.99	573.9063
RAB5A	2395.776	2297.337	2360.531	2255.717
RAB5B	7374.129	7091.401	9611.695	9258.809
RAB7A	15886.18	16547.46	16434.09	15200.46
RAB7B	16.30159	3.569655	2.441792	3.556065
RAB8B	2088.714	1816.817	1567.216	1227.405
RAB9A	597.783	563.1355	659.5437	618.6818
RAB9B	83.90563	87.30463	191.8222	202.1672
RABGEF1	3999.342	4373.524	3253.303	3080.129
RAC1	14221.2	15221.64	13095.63	11182.7
RACK1	59552.76	48445.83	53401.12	57401.6
RAF1	6077.109	6216.19	6119.822	5874.261
RALB	1559.199	1617.471	1695.582	1505.006
RARA	3069.901	3230.636	2782.114	2789.411
RB1CC1	3401.716	3520.599	3195.991	3055.157
RBX1	1376.338	1334.821	1518.405	1367.336
REL	545.5924	482.9164	491.5327	487.731
RELA	5122.565	5263.214	3969.881	3612.959
RELB	1400.848	1453.801	303.0654	249.3726
RETREG1	1640.192	1937.495	1635.789	1601.869
RETREG2	8568.024	8793.984	9960.75	9427.782
RETREG3	2302.975	2248.245	2703.722	2730.617
RGS19	307.4958	246.7318	237.6529	201.4279
RHEB	6222.204	5490.368	3542.903	3694.244
RICTOR	2450.68	2152.715	1909.197	1400.011
RIGI	377.8599	419.9673	469.3176	472.7441
RILP	662.955	763.0482	1845.63	1864.326
RIPK1	1540.973	1580.182	1352.673	1341.884
RIPK3	0	0	0.748192	0
RMC1	962.6297	1008.283	802.2061	744.1818
RNF152	121.7508	85.32104	175.6705	189.2865
RNF166	902.111	776.8372	716.7688	678.8843
RNF185	3970.443	4199.949	4208.052	3667.402
RNF2	791.3065	644.2535	778.9313	725.5349
RNF216	3196.966	3281.073	2883.355	2947.358
RNF41	3383.841	3425.714	2960.297	3087.244
RNF5	2782.619	2502.175	3547.226	3635.63
RPTOR	2628.126	3148.646	2517.923	2294.301
RRAGA	3495.423	3677.178	4097.319	4117.146
RRAGB	781.4353	798.6279	867.1811	820.6594
RRAGC	2361.167	2110.055	2554.045	2484.085
RRAGD	6436.959	6159.679	8692.438	9133.495
RTN3	7547.776	6372.991	8919.958	10058.99
RUBCN	2052.102	1800.043	1430.451	1246.707
RUBCNL	159.3924	325.3123	76.87056	55.68624
RUFY3	2016.899	2204.846	2261.207	2251.305
RUFY4	0	0	0	0
SAMM50	2195.907	2044.109	3519.175	3427.208

SAR1A	5702.178	5451.896	5059.705	4929.302
SAR1B	2618.575	2818.561	2753.185	2569.26
SBF2	1672.765	1756.256	2770.757	2730.083
SCOC	3235.51	3317.947	3707.908	3555.019
SEC13	3530.527	3657.911	3410.739	3131.055
SEC23A	4267.388	4246.445	4838.44	4258.869
SEC23B	3434.022	3493.969	3280.151	2981.241
SEC23IP	3278.032	3484.344	2684.649	2247.82
SEC24A	1653.344	1729.356	1745.696	1756.549
SEC24B	2281.08	2209.347	2499.52	2231.996
SEC24C	5220.429	5180.832	5880.275	5336.902
SEC24D	781.0165	703.615	901.5717	1042.665
SEC31A	7932.125	8092.998	8913.293	8217.328
SEC31B	782.1884	901.5862	1012.917	976.2542
SEC62	6953.353	6875.742	6523.134	5962.834
SEH1L	3696.751	2961.451	1607.628	1228.809
SESN1	1317.961	1463.553	1295.013	1362.748
SESN2	7307.721	8846.762	1769.64	2177.118
SESN3	235.4727	277.0771	1338.288	1556.813
SGSH	2565.784	3369.439	3305.348	3201.088
SH3BP4	4394.988	4781.237	2443.5	2007.713
SH3GLB1	3750.946	3332.492	4025.498	3520.947
SHOC2	1435.751	1286.293	1538.727	1548.212
SIAE	4600.766	5132.933	5723.96	6287.633
SIAH1	1309.336	1360.243	1770.103	2103.787
SIK2	4202.102	4663.81	5016.477	4618.026
SIRT1	1284.708	1208.422	1104.046	1161.938
SIRT2	2997.493	2806.188	3519.245	3700.708
SIRT6	1074.828	1146.345	949.2689	866.1842
SKP1	11591.06	11998.02	14323.09	12581.26
SKP2	368.917	381.6624	634.7466	567.4123
SLC25A4	3372.644	3299.355	5053.478	5119.463
SLC25A5	11592.2	10649.72	12632.13	11897.76
SLC38A9	542.2331	458.4113	439.5744	428.6167
SMCR8	3488.886	3570.385	3209.974	3000.044
SMPD1	4331.245	5467.661	7407.041	8117.449
SMURF1	2490.37	2441.945	2141.526	1946.312
SNAP29	2374.609	2504.654	2419.421	2345.772
SNAPIN	1093.427	1025.533	1271.208	1297.866
SNCAIP	4.340397	5.408732	3.50389	1.003922
SNX14	1953.029	1903.646	2839.204	2489.78
SNX18	1644.675	1650.514	1504.743	1509.065
SNX4	1735.936	1601.057	1874.505	1840.391
SNX7	767.4973	727.1062	1178.689	1238.6
SOX2	0	0.751608	0	0
SPATA18	0	0	1.740063	0
SPG11	2187.405	2131.163	3420.173	2962.092
SPHK2	1396.886	1707.293	1741.875	1724.76

SPNS1	4165.717	4691.393	5010.352	4623.372
SPP1	99.48579	133.4141	179.8787	271.832
SQSTM1	89968.71	84251.64	43794.16	41091.32
SRC	8947.843	8154.485	6121.715	4961.662
SREBF1	8998.423	8627.856	9573.597	8984.546
SREBF2	26887.32	24649.47	30780.49	29356.64
ST13	14760.94	13052.19	14582.62	15077.63
STAT1	3454.305	3742.085	4477.753	3997.094
STAT3	6071.664	5147.335	8143.34	7952.667
STAT6	4637.554	5525.447	5088.434	4745.293
STBD1	679.8908	389.2109	506.5204	505.473
STING1	590.0143	803.5794	834.0814	533.4122
STIP1	7978.36	6543.531	4909.562	4423.133
STK11	4630.992	4662.724	4645.285	4577.481
STK3	578.3151	547.7668	872.4148	838.2687
STK38	2679.986	2562.222	3558.434	2982.673
STK4	2297.559	1950.054	1852.328	1772.096
STRADA	829.6323	949.3979	1468.286	1349.598
STS	848.6259	906.8028	977.1014	989.1759
STUB1	4595.141	4521.72	4160.082	4320.859
STX12	1381.452	1087.453	1559.335	1482.344
STX17	1574.178	1371.285	1241.44	1356.013
STX6	2032.304	1981.747	2256.268	2051.219
STX7	2115.346	1911.856	2854.087	2617.095
STX8	464.9374	453.6372	777.2821	739.1774
SUPT20H	1617.35	1527.812	1459.714	1393.024
SYNPO2	2.066436	1.315224	1.268958	0.203632
SYT11	6761.225	6787.397	11430.8	10047.96
SZT2	2280.778	2911.993	2494.179	2257.883
TAB2	3196.278	2938.694	4342.272	4582.461
TAB3	2669.817	2595.128	2390.858	2362.708
TANK	1502.859	1818.08	1450.416	1542.609
TAX1BP1	9783.117	10267.1	11134.87	10538.07
TBC1D14	6111.47	5854.878	9753.868	10340.78
TBC1D15	1888.556	1828.994	1693.991	1610.715
TBC1D17	2718.818	2818.839	4300.001	4161.27
TBC1D2	5862.904	6742.953	5823.225	4396.758
TBC1D25	968.213	839.2451	1106.416	1042.309
TBC1D5	2388.72	1701.913	4128.918	3867.73
TBC1D7	1496.747	1447.01	904.7581	698.8742
TBK1	1409.357	1427.443	1362.635	1238.067
TCF7L2	1451.836	1608.153	1108.758	1217.075
TCIRG1	876.2728	842.7798	1188.06	1091.502
TECPR1	1272.318	1408.275	1807.906	1683.644
TECPR2	2507.922	2851.533	2327.894	2219.339
TELO2	3072.997	3385.416	2191.493	1926.296
TEX264	2457.958	2642.855	3710.369	3777.261
TFE3	5695.757	5913.592	4120.398	3852.621

TFEB	939.4973	590.7391	1216.901	1342.629
TICAM1	712.9945	626.6659	469.4836	513.2068
TIGAR	1854.783	2075.994	1205.584	1124.798
TLR4	228.8335	272.0966	543.6795	554.7259
TMEM106B	3500.124	3600.222	7013.684	6513.038
TMEM150B	5.288458	7.682954	4.389825	12.42396
TMEM175	1148.008	1391.961	1646.136	1866.403
TMEM41B	2377.672	2155.971	1722.498	1619.004
TMEM59	19731.03	23784.76	24888.91	31933.08
TMEM74	4.254971	7.107255	9.920317	5.198257
TNFAIP3	292.1576	590.0045	228.3527	182.9784
TOLLIP	6145.459	6525.645	5588.592	5430.298
TOM1	4007.059	4261.241	5090.742	5106.266
TOMM7	1529.031	1198.153	1438.744	1570.287
TP53	4860.46	4926.903	2792.235	2416.413
TP53BP2	5396.208	5642.823	3847.547	3702.688
TP53INP2	5887.23	4655.371	6533.89	6623.82
TP63	130.1317	123.487	163.5791	118.1098
TP73	17.70915	31.06579	18.50106	24.02741
TPCN1	11811.98	12788.03	11270.98	8060.645
TPCN2	845.923	869.8768	615.1113	535.9771
TPP1	28475.12	31847.13	40607.31	38160.33
TPT1	117891.8	105571.8	103649	107150
TRAF2	723.891	553.0511	517.3714	451.4501
TRAF6	1059.663	1131.753	1330.877	1171.012
TRAPPC1	2109.892	2051.845	3076.075	2769.311
TRAPPC11	1427.331	1485.27	1707.162	1550.435
TRAPPC12	1751.104	2180.215	2409.674	2262.801
TRAPPC13	1098.482	1093.084	1033.367	967.8592
TRAPPC3	2501.073	2613.347	3054.724	2792.934
TRAPPC4	2299.481	2477.089	2029.352	1916.92
TRAPPC5	2251.07	2480.671	2525.359	2637.33
TRAPPC8	2707.205	2698.663	2821.639	2574.149
TREM2	0	0	0	0
TRIB3	22011.53	23076.43	2486.449	3431.769
TRIM13	1395.892	1270.218	1192.198	1191.656
TRIM16	2066.385	1587.59	1478.85	1872.189
TRIM17	47.21667	70.09363	33.556	49.65608
TRIM21	455.3794	393.2222	484.3098	438.8094
TRIM23	1010.138	1045.297	998.3139	1042.536
TRIM28	14028.68	14620.47	12770.92	11975.72
TRIM5	602.4815	656.0031	1424.467	1195.008
TRIM65	1604.613	1160.788	1366.801	1278.886
TRIM8	5557.383	5737.15	6381.323	7201.13
TSC1	2623.93	2847.371	2972.979	2634.938
TSC2	3780.935	4089.115	4700.825	4454.946
TSG101	3540.914	3305.706	2820.603	2683.422
TSPO	5261.72	4838.155	5915.058	5889.741

TTI1	1157.418	1107.872	1174.56	1112.555
TUFM	10587.79	10348.63	12063.68	10646.58
TXNDC5	11567.51	10976.91	11700.14	10461.28
UBA5	1444.671	1636.09	1821.228	1853.467
UBAP1	4015.242	4337.958	3606.073	3362.731
UBE2D2	5230.55	4619.248	3600.096	3638.288
UBE2D3	12463.3	11043.55	9876.109	9465.555
UBE2G2	5146.937	5193.456	3384.876	3136.242
UBE2L3	4239.931	4289.027	4150.814	3946.874
UBE2N	3540.854	3519.906	2388.458	2118.979
UBXN6	7732.869	8300.855	9387.877	9399.121
UCHL1	10300.97	8652.004	9172.936	8441.05
UFC1	2200.071	1989.245	2582.765	2798.335
UFL1	1628.245	1680.216	1739.424	1505.894
UFM1	3455.584	3534.793	2702.219	2694.148
ULK1	3651.951	3668.067	5279.006	5626.764
ULK2	2775.189	3135.22	3344.425	3458.849
ULK3	1918.691	1908.46	1974.204	1910.856
UMAD1	713.5776	761.2615	1051.28	1016.247
USP10	4325.873	3996.346	3016.781	2542.638
USP13	974.2337	908.512	868.6446	771.7189
USP14	3987.967	3172.827	2466.116	2119.023
USP15	1706.809	1539.12	1263.764	995.7805
USP22	18083.73	17327.91	18540.36	17755.24
USP30	680.5545	798.8772	1581.955	1676.528
USP33	3563.309	3687.359	5803.41	5900.363
USP35	723.7194	860.4468	1028.687	954.624
USP36	4534.512	4115.215	2266.446	2055.029
USP8	2695.742	2693.742	2686.228	2432.44
USP9X	5469.863	5064.144	5536.695	5236.996
UVRAG	1017.752	1058.086	1279.189	1258.118
VAC14	4713.34	5182.42	4992.694	4143.274
VAMP2	2264.818	2526.787	3924.229	4320.322
VAMP3	5374.667	5718.796	6002.683	5617.476
VAMP7	1880.54	1751.047	1825.215	1748.106
VAMP8	13.65354	23.19094	22.70732	37.55287
VAPA	6547.035	6029.394	5369.557	5346.15
VAPB	3328.455	2995.608	2873.36	3002.067
VCP	19158.96	20552.96	19925.28	17552.16
VDAC1	10149.98	10359.91	9049.747	8238.887
VMP1	3227.251	3378.273	2876.617	2613.575
VPS11	2716.414	2850.703	3752.004	3367.309
VPS13D	2701.421	2687.202	2808.105	2483.019
VPS16	1212.146	1131.967	1829.528	1521.996
VPS18	3784.81	3815.293	3913.118	3580.591
VPS28	1197.552	1253.836	1497.157	1284.936
VPS33A	1522.886	1490.464	1733.688	1460.292
VPS37A	1973.974	2034.397	1764.122	1576.793

VPS37B	3263.375	3218.575	2428.813	2230.428
VPS37C	1869.874	2061.415	1760.403	1657.819
VPS37D	181.6779	144.1546	192.5866	241.4351
VPS39	4271.752	4379.687	4325.642	4333.249
VPS41	4819.338	4877.459	5601.338	5248.641
VPS4A	6244.583	6256.544	5009.35	4823.951
VPS4B	2440.024	2311.088	1971.232	1879.673
VTI1A	944.1648	1139.145	1015.383	864.0265
VTI1B	5396.794	5257.652	5047.974	5558.72
WAC	5819.576	5904.511	5652.627	5679.175
WASHC1	4386.13	5412.869	3899.164	3827.643
WDFY3	2454.913	2376.262	3736.784	3222.746
WDR24	1369.646	1668.709	1768.591	1837.42
WDR41	978.6999	960.052	1033.222	1075.858
WDR45	3597.443	4215.178	3201.582	3252.095
WDR45B	4210.076	4335.3	3334.66	3355.121
WDR59	1941.667	1980.225	1872.002	1789.616
WDR81	2552.796	3058.536	2886.666	2709.963
WDR91	337.8001	332.0193	620.014	568.3306
WHAMM	883.0794	1162.419	996.8981	978.4816
WIPI1	1068.064	1079.37	1476.025	1416.511
WIPI2	4492.889	4877.144	4665.297	4553.515
WWTR1	5762.738	6237.488	6616.387	6084.718
YAP1	11566.22	10842.12	9423.48	9123.481
YKT6	5105.056	5630.95	3930.469	3525.633
YOD1	1915.357	1990.177	1839.333	1386.573
YWHAE	17365.3	16680.79	17222.53	15612.1
YWHAG	13623.81	11837.81	9517.425	9156.69
YWHAZ	27710.35	21345.68	23355.14	22510.98
YY1	6464.98	5804.842	5627.719	5613.249
ZFYVE1	1376.65	1477.802	2056.654	2136.528
ZFYVE26	2618.587	2599.295	3473.264	2962.206
ZKSCAN3	268.0029	269.0536	404.7453	421.7885

Supplementary Table S6: Ubiquitin proteosome pathway genes

Gene	POS (4Hrs)	POS (6Hrs)	POS (24Hrs)	POS (48Hrs)
ABTB1	802.7841	1122.024	1488.023	1490.936
ABTB2	1100.807	1025.415	490.3364	421.8174
ABTB3	289.1179	246.2432	301.4811	360.312
ADRM1	5585.121	6020.91	3732.047	3423.413
AKIRIN1	4206.531	4173.749	3919.092	4207.957
AKIRIN2	2713.5	3118.938	3252.355	3574.098
ALG13	1120.173	1170.685	1038.908	900.9878
AMBRA1	2947.726	3318.377	2976.631	2683.903
AMFR	7752.562	8256.74	8327.31	8511.277
AMN1	300.0129	265.4912	427.6393	469.7134
ANAPC1	3152.732	3055.282	2610.869	2327.015
ANAPC10	270.3554	236.7486	223.3005	229.6377
ANAPC11	1825.673	1910.49	2179.069	2398.589
ANAPC13	1886.8	1745.623	2131.449	2011.133
ANAPC15	1082.671	1156.844	1038.794	889.6125
ANAPC16	2833.403	2544.538	3543.814	3335.862
ANAPC2	2498.51	2823.101	2969.462	2821.465
ANAPC4	618.5516	588.4099	690.6889	567.9803
ANAPC5	4321.123	4391.664	4931.941	4607.508
ANAPC7	2986.684	2964.947	2515.692	2192.328
ANKFY1	3914.761	3791.464	4617.064	4218.374
ANKIB1	3782.212	3612.716	3693.509	3465.126
ANKRD60	1.24358	0.496846	0.23496	0.248721
ANKRD61	16.76837	20.19345	10.24816	7.503415
ANKRD9	5465.24	5373.761	7197.154	7642.605
ANKUB1	0.246188	0.247281	4.953218	3.089922
ANKZF1	1011.813	1193.538	1342.793	1427.996
APPBP2	1874.996	1888.704	2302.675	2368.539
AREL1	4444.497	4480.433	3813.952	3460.306
ARIH1	3307.762	2949.171	3390.246	3189.492
ARIH2	6281.603	6393.53	4212.45	3896.105
ARMC5	1159.839	1446.414	979.1104	951.3269
ARMC8	2444.063	2133.501	1719.094	1509.246
ARRDC1	1346.718	1536.473	1346.169	1157.394
ARRDC2	806.3344	605.3488	345.9835	344.6274
ARRDC3	1364.038	1630.008	2554.981	2181.471
ARRDC4	585.1697	540.9451	753.7735	811.5335
ARRDC5	3.008988	3.225298	6.540573	4.514395
ASB1	2371.733	2539.49	1539.13	1329.506
ASB10	0	0	0	0
ASB11	0	1.103069	1.315001	0
ASB12	10.25466	13.4699	19.28108	24.50409
ASB13	1767.666	1542.315	1318.429	1140.687
ASB14	13.01656	21.773	15.15948	7.814412

ASB15	0	1.002175	0.750349	0
ASB16	42.92483	57.78316	45.42624	34.09105
ASB17	0	0	0	0
ASB18	1.244755	1.244098	0.748193	0
ASB2	5.959293	3.870728	1.697822	1.269743
ASB3	272.4103	362.2836	485.9279	424.0797
ASB4	0.872503	0.501465	1.10628	2.249418
ASB5	2.79798	3.331265	4.792646	0.662685
ASB6	4927.985	4502.133	3532.854	3320.927
ASB7	881.6875	993.3592	983.8889	952.5328
ASB8	886.9139	947.9732	1382.092	1383.48
ASB9	27.96238	24.75367	135.5065	77.21083
ASCC2	2465.725	2455.959	2335.113	2085.668
ASPSCR1	1478.839	1625.034	1470.599	1448.301
ATF7IP	1764.851	1935.98	3737.639	3568.545
ATXN3	711.8619	610.4339	814.2292	764.466
ATXN3L	0	0	0	0
ATXN7	1119.698	1210.508	1207.101	1111.512
ATXN7L1	249.2043	358.6509	394.3702	327.6632
ATXN7L2	463.8385	408.7262	393.6972	358.7887
ATXN7L3	4168.323	3442.087	2834.736	2783.19
AUP1	5333.068	5295.691	4455.035	4011.471
BABAM2	1111.672	1127.594	1778.179	1540.769
BAG1	7046.34	7321.246	6300.993	6341.863
BAG2	5209.773	3942.933	2306.177	1853.081
BAG6	6408.107	7162.851	8472.846	7431.858
BAP1	6918.787	7086.345	6625.232	6168.718
BARD1	176.8287	169.6977	252.6365	273.1724
BFAR	2691.709	2595.496	2574.366	2356.666
BIRC2	1112.487	1132.351	1024.426	1060.562
BIRC3	121.6081	213.0737	78.57311	56.64194
BIRC6	2809.227	2702.616	2489.723	2148.235
BIRC7	135.9036	216.0434	233.8356	251.4821
BIRC8	0	0	0	0
BMI1	3477.424	2954.036	3987.166	3926.358
BRAP	1647.842	1640.588	1182.741	1186.133
BRCA1	19.28475	15.30903	22.71391	24.60851
BRCC3	1171.948	1149.646	1246.248	1132.333
BRSK1	807.8814	809.9183	803.4866	908.2753
BRSK2	649.5338	684.4962	609.872	688.6028
BRWD1	3391.679	2790.252	1880.771	1873.986
BTBD1	2967.971	2526.422	2327.062	2118.393
BTBD10	954.3522	859.6383	927.2284	937.6881
BTBD19	1571.587	1853.649	1537.851	1529.146
BTBD2	5810.657	6652.522	8382.907	8031.494
BTBD3	1688.98	1525.517	1283.988	1250.6
BTBD6	2256.285	2104.76	2329.731	2482.369
BTBD7	1934.047	1836.954	2038.995	1748.385

BTBD8	117.2837	147.0603	319.4261	329.2274
BTBD9	1311.329	1091.693	1395.163	1135.895
BTRC	1302.444	1208.694	1427.909	1414.65
C10orf90	0.266474	0.267324	0	0.648944
CACUL1	3159.779	2891.702	2523.177	2468.921
CAND1	4853.949	4736.98	5315.736	4988.903
CAND2	347.0169	342.4876	535.5503	619.59
CAPN15	5428.72	5815.376	2720.249	2264.982
CBL	1585.399	1022.192	1055.576	1071.576
CBLB	1115.675	1044.377	1667.02	1829.402
CBLC	0	0	0	0.250928
CBLL1	1391.159	1310.059	938.8897	888.5626
CBLL2	0	0	0	0
CBX4	1288.968	1268.075	1429.59	1583.242
CCIN	0	0.752165	1.001392	0
CCNB1IP1	2377.971	2036.609	1945.968	2198.08
CCNF	270.9394	202.8551	210.0925	239.6361
CDC16	3547.379	3508.378	2903.547	2813.141
CDC20	142.7798	77.6424	88.68437	151.483
CDC20B	0	1.256139	0	0
CDC23	886.2802	895.6298	939.735	880.5855
CDC26	596.8629	486.1391	645.7175	614.6495
CDC27	2889.592	2693.025	2549.823	2173.945
CDC34	2973.581	2596.812	2336.911	2259.928
CDK2	443.4659	330.2956	653.9786	648.0276
CDK5RAP3	5648.75	5944.391	6039.642	5735.219
CENPE	58.8735	29.03399	33.47337	54.41584
CFAP410	1691.363	2087.523	2160.832	2129.337
CGRRF1	517.5285	511.632	574.7075	528.8528
CHFR	1544.79	1465.09	1150.698	1054.457
CHUK	2245.27	2024.638	1788.941	1707.264
CIAO1	5456.909	5925.085	5128.636	4864.996
CISH	15.73375	25.16996	25.96868	21.2199
CMYA5	62.40163	52.51927	104.8327	114.1293
CNOT4	817.8269	845.701	903.8483	908.5761
COBL	2098.748	2265.995	2090.142	2064.933
COBLL1	2605.614	2992.341	3784.916	4125.795
COMMD1	644.1923	555.393	663.4146	646.6616
COP1	1663.73	1664.966	1627.895	1565.207
COPS2	2999.849	2899.509	3065.951	2995.122
COPS3	2060.711	1741.726	1570.033	1553.048
COPS4	1389.762	1388.96	1744.487	1563.448
COPS5	1874.157	1888.853	1688.601	1657.861
COPS6	3447.865	3164.38	4070.479	3480.465
COPS7A	2669.793	2705.682	3958.122	3472.486
COPS7B	1312.423	1254.396	1264.893	1176.826
COPS8	3032.01	3190.919	2405.004	2263.248
CPSF1	3170.465	3318.83	3501.025	3279.417

CRBN	1313.997	1272.498	1451.001	1419.382
CREBBP	4869.958	5059.413	4639.244	4640.218
CUEDC1	2839.437	3312.774	2851.448	2897.927
CUEDC2	3009.465	2954.343	3369.294	3301.319
CUL1	3872.703	3548.411	3078.604	3124.702
CUL2	1662.451	1569.43	1590.415	1582.58
CUL3	4163.738	3436.829	3228.977	3126.392
CUL4A	7556.294	7281.068	4787.916	4383.797
CUL4B	3098.363	3128.581	4227.236	4026.963
CUL5	1887.856	1905.288	1673.251	1637.436
CUL7	1712.189	1742.415	3115.832	2662.311
CUL9	1338.228	1615.844	2225.34	2047.222
CYLD	4277.106	5095.37	2675.994	2535.04
DAXX	2613.193	2188.076	2153.887	1985.464
DCAF1	2127.658	1920.476	1703.332	1543.416
DCAF10	2428.491	2658.761	2315.201	2174.65
DCAF11	3940.683	3942.754	5126.056	4860.527
DCAF12	3286.589	2843.38	2409.088	2353.132
DCAF12L1	0	0	0	0
DCAF12L2	2.247919	8.737538	15.72157	14.9685
DCAF13	2649.803	2540.37	1958.856	1489.475
DCAF15	791.9148	819.753	817.5529	769.2243
DCAF16	1697.84	1491.228	1395.045	1225.25
DCAF17	1180.371	1185.396	1018.446	946.8238
DCAF4	1136.279	835.7118	624.6552	557.7934
DCAF4L1	2.994121	2.746375	3.241231	0.249394
DCAF4L2	0	0.251006	0	0
DCAF5	1935.005	1856.966	2415.644	2384.065
DCAF6	1785.821	1699.388	2866.308	2876.594
DCAF7	7369.606	7687.504	7391.016	6766.618
DCAF8	4972.654	5097.498	6041	5589.544
DCAF8L1	0	0	0	0
DCAF8L2	3.232895	1.243515	0	0.249762
DCLRE1A	595.2488	566.1619	815.5141	874.0221
DCST1	0.500051	0.248506	0	0
DCUN1D1	1122.667	1188.527	1197.169	1121.25
DCUN1D2	1052.566	1006.345	766.9459	798.4393
DCUN1D3	1171.705	1264.188	954.8669	898.8125
DCUN1D4	2254.543	1801.223	1708.174	1833.351
DCUN1D5	1509.008	1242.853	853.5549	745.9351
DDA1	2582.352	2880.255	2145.254	2090.853
DDB1	14602.67	15160.07	17256.04	14644.51
DDB2	837.874	1171.789	1884.379	1778.369
DDI1	0	0	0.994531	0
DDI2	2651.753	2135.692	2483.52	2459.137
DDRGK1	2463.25	2626.62	2556.776	2454.062
DERL1	3013.299	3055.423	2782.962	2697.231
DERL2	2129.676	1939.123	1608.24	1579.206

DESI1	3434.097	2952.175	2264.445	2189.78
DET1	481.4208	401.6066	473.4742	424.0072
DHX57	1236.757	1214.281	1127.807	909.3817
DMRT3	0	0	0	0
DMRTA1	742.6842	857.5972	1181.759	1030.449
DMRTA2	0	0.292243	0	0
DNAJB2	3891.654	3949.412	4557.088	4607.639
DPP9	5174.344	5478.302	4086.224	3164.26
DTL	101.667	37.79198	99.46719	94.16945
DTX1	0	0.248583	0	0.249548
DTX2	603.154	595.0502	626.2432	562.872
DTX3	2686.547	2844.462	3479.578	3523.324
DTX3L	2304.043	2376.188	2523.169	2210.422
DTX4	1448.216	1476.169	2651.251	2283.496
DZIP3	452.539	501.2656	870.3769	826.3923
E4F1	1443.095	1350.343	1123.592	1041.251
ECPAS	8052	7235.42	6527.954	6557.254
ECT2L	2.162547	1.440404	2.639955	0.240558
EED	395.6881	363.4459	448.3084	400.8706
EGR2	91.12015	127.845	73.753	34.6013
EIF2AK4	2039.111	2037.207	2380.711	2416.402
ELOA	2919.775	2790.758	2109.277	1836.362
ELOB	6346.684	6898.377	9199.611	7235.856
ELOC	1981.073	1874.832	1358.224	1286.282
ENC1	2196.587	2431.772	2861.423	2636.368
ENTREP1	396.506	420.3532	534.0559	450.7077
ERCC5	1302.531	1388.877	1828.497	1699.747
ERCC6L	29.26918	21.54065	17.7571	22.11082
ERCC8	539.3868	521.4276	548.4659	430.186
EWSR1	9890.999	9443.547	7334.852	6636.163
FAAP20	1959.921	2315.62	2482.103	2415.21
FAF1	1937.374	1983.387	2361.63	2201.973
FAF2	3457.584	3642.418	3332.849	2926.445
FAN1	1366.929	1126.993	1109.036	1151.131
FANCL	489.7454	506.4769	782.371	639.9398
FAU	8909.983	7992.319	7882.524	7797.947
FBH1	3176.457	3287.394	3306.434	3098.197
FBXL12	961.7907	996.968	797.7762	811.7462
FBXL13	72.91658	89.23816	62.19444	84.77595
FBXL14	780.4889	881.8649	823.2861	927.2162
FBXL15	1647.158	1566.869	1538.167	1507.033
FBXL16	6.727968	10.00293	14.95624	11.71371
FBXL17	668.2814	758.4912	1176.057	1185.261
FBXL18	1820.62	1632.135	1837.57	1531.915
FBXL19	1791.302	1610.5	1469.961	1444.509
FBXL2	1374.849	1518.42	1409.016	1565.672
FBXL20	883.219	835.2996	1109.676	1023.233
FBXL21P	0	0	0	0

FBXL22	24.08238	22.32634	32.20081	24.35566
FBXL3	1718.571	1667.535	1936.813	1670.889
FBXL4	1869.818	1753.78	2069.935	1943.833
FBXL5	2562.16	2389.745	2940.722	2735.855
FBXL6	961.8588	1219.88	614.5603	432.4684
FBXL7	812.608	942.2709	1007.765	908.4328
FBXL8	359.2777	459.3371	654.1549	615.8973
FBXO10	1470.2	1478.864	1630.438	1588.7
FBXO11	2389.129	2328.649	2757.168	2537.918
FBXO15	13.41893	20.27629	66.09656	75.60105
FBXO16	9.334709	19.63712	59.3312	59.94504
FBXO17	1673.709	1695.237	1760.392	1838.082
FBXO2	881.9859	723.879	681.0205	1409.257
FBXO21	3589.77	3463.795	3731.204	3486.358
FBXO22	1847.301	1853.474	1427.928	1267.018
FBXO24	6.008079	3.339974	11.31633	15.41369
FBXO25	1736.139	1681.796	1478.635	1582.129
FBXO27	1131.508	966.3986	1263.052	1522.443
FBXO28	2143.011	2375.82	2971.201	2782.505
FBXO3	1230.654	1244.991	2172.096	1957.823
FBXO30	1262.611	1298.1	1134.778	992.6708
FBXO31	3987.852	3669.687	2324.348	2205.449
FBXO32	22976.16	25293.35	13101.45	11358.17
FBXO33	1051.323	1096.284	1840.296	1731.821
FBXO34	1768.301	1533.333	1798.999	1985.579
FBXO36	285.1236	383.5949	437.3116	380.6727
FBXO38	1814.536	1854.723	1650.311	1504.017
FBXO39	0	0	0	0
FBXO4	264.0366	297.2955	350.7085	336.2358
FBXO40	0	0	0	0
FBXO41	2739.065	3149.293	1848.235	1768.268
FBXO42	990.1389	1060.959	959.8456	913.7994
FBXO43	8.135694	4.854743	3.960573	9.646932
FBXO44	2233.284	2296.627	3236.899	3614.182
FBXO45	722.2472	610.6492	548.2308	503.6605
FBXO46	1098.924	981.2578	1306.606	1207.967
FBXO47	0	0	0	0
FBXO48	241.2742	48.52259	38.12098	61.37913
FBXO5	343.1733	314.6525	228.2808	220.2798
FBXO6	375.9149	408.9065	509.3764	612.1091
FBXO7	5359.582	5257.219	5678.891	5694.657
FBXO8	816.6172	609.8235	949.4369	933.3514
FBXO9	3196.875	3275.159	3318.926	3035.705
FBXW10	15.58042	15.57761	14.34889	29.94177
FBXW10B	29.29125	38.30442	32.23952	17.98606
FBXW11	3982.185	3640.287	3321.896	3405.808
FBXW12	0	0	0	0
FBXW2	4743.073	4847.658	3956.325	3518.08

FBXW4	2729.969	2861.651	2649.216	2815.583
FBXW5	7115.174	7605.483	7053.043	7014.44
FBXW7	798.7306	695.3246	486.6463	467.7161
FBXW8	2015.45	2354.655	1753.555	1606.182
FBXW9	997.7697	1102.615	861.1489	774.2747
FEM1A	3251.18	3433.042	2644.784	2633.737
FEM1B	3838.384	3318.88	3100.197	3159.493
FEM1C	899.583	892.0243	856.6825	849.1868
FUS	10042.86	10081.57	5852.592	5131.731
FZR1	1835.469	1822.389	2435.586	2239.23
G2E3	831.8168	788.2977	733.0066	743.1499
GAN	752.5024	520.4391	419.0113	387.4672
GID4	1180.316	1029.385	1086.343	1186.101
GID8	3342.043	3329.462	2841.057	2759.162
GLMN	291.7991	318.787	348.9906	251.0362
GMCL1	1327.531	1288.351	1492.655	1640.693
GMCL2	0	0	0	0
GNB2	10000.75	10232.05	9163.126	9337.572
GPS1	4205.84	4753.908	5014.434	4581.887
GRWD1	4505.103	4111.688	2495.425	2184.659
HACE1	778.6579	788.5289	902.328	961.5712
HBS1L	2136.944	1986.288	1945.843	1881.562
HDAC6	2339.083	2246.722	2858.143	2845.312
HECTD1	8949.083	9146.535	7718.689	7936.458
HECTD2	462.8272	421.4934	359.0746	347.4848
HECTD3	2890.777	3242.201	2971.092	2611.863
HECTD4	2039.865	2138.802	2295.065	2181.663
HECW1	0.990977	4.503664	0	2.767824
HECW2	65.64597	70.5275	39.18545	62.60372
HEMK1	736.1046	724.4593	1167.609	1146.589
HERC1	1259.734	1257.644	2208.086	2006.666
HERC2	1145.052	1118.974	1115.887	868.4448
HERC3	1306.774	979.9592	1263.909	1069.865
HERC4	1854.082	1642.583	1538.274	1319.108
HERC5	99.13322	137.681	190.4681	191.8436
HERC6	238.604	249.7276	520.294	524.8227
HERPUD1	4292.851	4269.254	6324.654	6597.141
HERPUD2	1419.374	1620.809	1546.552	1427.975
HIPK2	4264.067	4155.431	3315.264	3417.355
HLTF	1383.041	1297.874	2891.393	2786.518
HUWE1	9002.564	8086.092	9109.357	8000.746
HYPK	88.61151	76.53162	28.75072	25.15935
IBTK	4324.643	3984.546	3235.742	2912.321
IKBKB	2247.051	2511.274	1871.535	1798.213
IKBKE	128.3595	125.8868	203.186	168.8494
IKBKG	1244.166	1490.643	970.6917	875.0615
ILRUN	10839.99	9684.369	10213.3	9997.245
IMPACT	2488.536	2399.611	2692.051	2649.758

IPP	280.9518	296.8649	501.3818	444.4721
IQUB	11.1064	8.941386	36.86595	60.8351
IRF2BP1	2641.666	2563.061	2525.776	2658.873
IRF2BP2	6658.957	6053.856	6600.164	6649.551
IRF2BPL	4788.164	5971.206	6646.324	6189.353
ISG15	170.788	176.9248	267.7129	304.8173
ITCH	3453.866	3240.926	3214.663	3000.732
IVNS1ABP	8214.389	7209.425	9348.527	8597.891
JADE2	2555.529	2463.381	4355.809	5009.578
JOSD1	3573.885	3261.494	2730.272	2753.794
JOSD2	1306.541	1389.57	1669.529	1718.141
KATNB1	1528.73	1801.127	1282.873	1172.99
KBTBD11	7710.365	7966.679	3953.761	2681.606
KBTBD12	141.1126	197.07	207.1953	204.1472
KBTBD13	0	0	0	0
KBTBD2	1340.107	1414.751	1249.314	1221.237
KBTBD3	128.4902	121.2666	293.4679	304.239
KBTBD4	593.1935	626.8127	943.6951	905.7063
KBTBD6	1505.847	1360.155	1405.706	1214.158
KBTBD7	679.9173	685.7456	1065.6	1013.316
KBTBD8	104.9944	141.1732	100.4494	61.5596
KCMF1	2838.274	2771.806	2813.469	2858.807
KCTD10	2893.365	2778.845	3122.987	3018.749
KCTD11	1704.577	1636.219	1774.252	1497.47
KCTD13	1011.08	1123.71	547.364	541.1285
KCTD17	850.6498	957.0291	788.683	802.2459
KCTD2	2742.431	2734.281	2844.295	2629.379
KCTD20	2514.442	2332.83	2664.347	2634.904
KCTD21	764.0207	720.4252	728.8099	754.0074
KCTD3	2705.861	2976.103	3204.124	3155.953
KCTD5	1640.732	1684.973	1322.654	1507.388
KCTD6	346.5494	304.7335	338.9736	322.7467
KCTD9	1110.604	887.6703	893.8406	858.6257
KDM2A	3366.164	3139.662	3358.912	3217.032
KDM2B	1685.677	1635.744	1433.242	1367.877
KEAP1	5136.063	4650.983	5046.455	5396.198
KHNYN	3178.533	3203.553	2286.135	2204.1
KLF4	288.4455	374.8782	87.34613	93.38946
KLHDC1	41.71273	75.37285	68.85673	99.17932
KLHDC10	3157.28	3384.925	4165.038	3880.014
KLHDC2	2238.107	2401.136	3983.643	4413.581
KLHDC3	2834.663	3059.276	3188.617	3204.12
KLHL1	0	0	1.494219	0.998271
KLHL10	3.041502	2.662357	3.426106	2.733049
KLHL11	836.8335	847.7701	837.8481	746.0103
KLHL12	1594.06	1539.609	1706.726	1633.979
KLHL13	940.1299	1355.086	1363.295	1296.449
KLHL14	0	0.367669	2.59058	2.568788

KLHL15	656.4443	702.7463	636.8498	610.5899
KLHL17	704.0904	650.5935	620.3979	561.89
KLHL18	1400.903	1223.17	1063.475	1047.074
KLHL2	1323.281	1213.997	1243.687	1267.339
KLHL20	586.5084	596.4139	651.5995	659.4544
KLHL21	9320.183	10996.27	13487.82	14947.67
KLHL22	1509.253	1599.135	2167.296	2126.837
KLHL23	459.6659	289.2747	118.5816	137.36
KLHL24	2295.657	2322.082	4003.587	3997.068
KLHL25	388.3974	371.9219	311.0153	338.4555
KLHL26	666.9537	602.7395	611.4278	583.8796
KLHL28	477.9693	541.7974	516.053	493.2893
KLHL29	4100.194	3740.767	1611.707	1613.773
KLHL3	372.9933	527.425	2242.851	3071.345
KLHL30	127.7556	132.904	71.60937	60.60958
KLHL31	38.3759	37.53588	62.3436	78.55167
KLHL32	5.618005	5.744202	12.659	21.76101
KLHL33	0	0.749066	0.621681	0
KLHL34	27.64799	40.9472	28.94053	26.43198
KLHL35	80.6523	110.7557	110.346	95.58674
KLHL36	3263.131	3122.13	3561.54	3054.403
KLHL38	4.401639	17.5397	4.779539	2.077709
KLHL4	85.65041	38.66707	75.42469	61.79858
KLHL40	0	0	0	0.99783
KLHL41	26.44185	26.47229	27.18679	23.95124
KLHL42	1848.771	1775.091	1452.684	1355.125
KLHL5	5973.729	4917.229	4043.432	4674.456
KLHL6	1.333603	0.499585	0	1.495004
KLHL7	1617.63	1469.259	1841.973	1706.101
KLHL8	594.0219	522.3964	561.7314	588.4919
KLHL9	2954.364	3067.792	2617.53	2348.541
LATS1	1706.522	1630.092	1716.014	1664.896
LATS2	1752.991	2353.258	1949.675	1768.294
LGALS3BP	32866.08	39280.65	46476.03	46252.95
LNX1	257.5067	309.8103	251.0644	281.7443
LNX2	1040.996	1031.45	1907.168	1826.915
LONRF1	563.564	424.3098	320.9824	299.9077
LONRF2	341.7966	345.7089	414.8981	252.8091
LONRF3	777.1361	660.3179	138.3202	152.0061
LRR1	236.5605	180.6085	216.5775	223.1803
LRRC41	6016.725	6641.282	5517.763	5400.114
LRSAM1	1214.818	1483.39	2084.965	1841.023
LTN1	1521.07	1415.003	1246.438	1143.944
LZTR1	3856.297	4372.845	3786.263	3416.18
MAEA	4237.392	4270.979	4727.84	4491.804
MAGEA1	0	0	0	0
MAGEA11	0	0	0	0
MAGEA12	0	0	0	0

MAGEA2	0	0	0	0
MAGEA3	0	0	0	0
MAGEA4	0	0	0	0
MAGEA6	0	0	0	0
MAGEB18	0	0	0	0
MAGEC2	0	0	0	0
MAGED1	7308.587	8042.259	15852.63	17098.64
MAGEF1	2517.739	2558.027	2460.575	2210.075
MAGEL2	0.500695	1.004296	0	0.748205
MAP3K1	670.5124	857.2657	850.3552	685.6987
MARCHF1	301.3211	395.756	190.959	208.1394
MARCHF10	0	0.481181	0	0
MARCHF11	1.423936	1.295979	0	0.936097
MARCHF2	848.4015	1000.598	1355.942	1476.053
MARCHF3	61.29374	32.22026	44.97699	40.32854
MARCHF4	621.8976	570.7008	148.6308	219.1338
MARCHF5	2643.716	2468.517	2637.706	2506.459
MARCHF6	6167.944	5975.232	6998.451	7081.247
MARCHF7	2637.631	2676.016	3631.133	3303.544
MARCHF8	1960.27	1770.427	2986.717	2830.662
MARCHF9	1189.163	1082.148	1458.338	1636.302
MARK1	872.584	837.34	482.4717	425.772
MARK2	2069.718	2102.954	1903.775	1971.146
MARK3	2857.078	2711.26	2538.617	2592.173
MARK4	2950.026	2697.266	2170.726	2326.749
MDM2	6131.15	7929.096	4418.336	3887.083
MDM4	1329.758	1449.631	991.8857	876.7669
MEFV	1.817785	1.562973	1.77412	1.222255
MEX3A	1115.088	646.7916	492.8433	423.926
MEX3B	346.8869	456.3221	536.2099	507.4747
MEX3C	2252.336	1950.363	2067.965	2008.505
MEX3D	3238.725	2303.841	2525.101	2527.711
MGRN1	6230.711	6378.917	4706.092	4649.194
MIB1	3864.096	3965.405	2955.272	2424.577
MIB2	3486.506	4053.366	4283.188	4582.437
MID1	1836.843	1696.34	1861.19	1655.85
MID2	1049.284	875.8163	967.135	974.2978
MIDN	2909.05	3780.285	2813.265	2476.464
MINDY1	357.86	519.5007	1960.494	2155.623
MINDY2	1915.212	1902.599	2030.563	1673.359
MINDY3	1253.586	1336.683	1486.91	1388.456
MINDY4	168.0719	205.3562	258.9429	277.4108
MKLN1	2198.113	1883.551	2611.847	2111.529
MKRN1	3219.614	3381.704	3850.541	3830.964
MKRN2	1910.669	1601.403	1968.379	1703.298
MKRN3	102.8445	91.89754	83.08174	84.81188
MNAT1	589.4998	553.0673	623.1205	656.9097
MOCS3	961.4584	939.1096	726.3346	644.0522

MPND	824.6717	747.1394	1037.951	1043.527
MSL2	1317.645	1243.824	1389.522	1288.083
MTA1	4309.279	4785.552	4586.494	4366.184
MUL1	3038.565	3739.299	3213.422	3011.442
MYB	15.80468	11.76867	4.488525	4.970581
MYCBP2	1540.306	1358.132	1788.789	1509.378
MYLIP	308.7926	431.5117	1046.416	1436.486
MYSM1	1520.779	1673.332	1156.342	996.877
N4BP1	1996.794	1842.763	2361.396	2324.665
N4BP2	333.2643	371.0195	293.5927	275.0248
NACA	23950.67	20609.63	21578.12	22436.68
NACA2	0	0.246447	0	1.260831
NACAD	297.6656	187.8431	360.4044	343.0513
NAE1	1938.523	1858.581	1681.447	1404.461
NDFIP1	10517.98	10501.42	14101.19	12114.9
NDFIP2	2433.397	2199.069	2145.467	1962.571
NEDD4	1309.468	1043.999	639.6153	613.0177
NEDD4L	5271.578	5634.344	8804.221	9743.839
NEDD8	1931.956	1721.71	2307.463	2054.692
NEIL3	22.39161	9.557674	19.26581	38.28042
NEURL1	3.734937	6.244223	2.749297	2.491209
NEURL1B	108.6072	91.14483	68.4327	164.3036
NEURL2	499.4777	613.4763	448.7917	543.6807
NEURL3	19.75532	33.16543	12.04399	7.57005
NEURL4	2219.39	2043.576	1874.904	1815.607
NFATC2IP	2159.114	2545.29	1809.213	1666.439
NFE2L1	28534.5	27733.39	37568.48	38707.77
NFE2L2	7336.62	8271.734	6841.443	7166.456
NFX1	3351.811	3158.332	2738.9	2570.018
NFXL1	511.5027	373.6166	421.4602	402.6918
NGLY1	2286.947	2023.229	2413.534	2219.789
NHLRC1	191.675	270.7753	324.1281	279.9566
NLE1	2009.25	1818.653	1218.453	1010.862
NOSIP	1272.482	1444.006	1241.06	1298.563
NPLOC4	9135.516	9543.676	9703.509	9523.56
NSFL1C	6890.797	7496.494	8693.627	8249.851
NSMCE1	1917.475	1720.245	2722.022	2742.903
NSMCE2	839.9507	861.9166	890.5695	807.995
NSMCE3	1848.584	1870.924	1528.345	1327.856
NUB1	2115.424	2168.296	2231.478	2278.334
NUP153	2878.85	2583.893	2159.775	1952.763
NXF1	3802.583	4536.329	3454.01	3232.871
NXF2	0	0	0	0
OASL	3.31089	0.581341	0.259306	0
OBI1	778.9181	801.6151	761.7332	680.454
OTUB1	4868.033	5213.025	4695.062	4225.486
OTUB2	96.36284	64.93653	153.91	148.9224
OTUD1	918.7131	1007.081	1291.679	1310.872

OTUD3	668.5741	634.4171	461.0976	457.1672
OTUD4	4889.126	4223.486	3249.318	3035.254
OTUD5	2440.305	2316.799	2388.853	2329.294
OTUD6A	0	1.474772	4.023783	1.27918
OTUD6B	1073.466	905.8578	481.9683	425.353
OTUD7A	214.7177	433.7575	155.7706	112.6631
OTUD7B	2235.031	2119.798	1906.411	1862.452
OTULIN	1003.299	1048.713	859.1751	888.5493
PAAF1	778.1872	800.8769	1045.842	1014.749
PAFAH1B1	7344.162	6460.387	7027.294	6778.581
PARP10	684.6288	834.9071	1371.001	1347.245
PCGF1	408.1843	399.2274	354.779	379.9823
PCGF2	535.1197	540.8825	711.6309	731.0502
PCGF3	2509.768	2517.725	2245.245	2187.498
PCGF5	3815.505	3494.941	3483.413	3166.674
PCGF6	698.1993	680.0772	447.4581	372.0893
PCMTD1	1654.856	1698.6	2826.635	3063.428
PCMTD2	2403.631	3361.127	3861.294	4069.72
PDZRN3	3243.236	2998.57	2471.313	2823.571
PDZRN4	1.746903	1.00038	4.746609	0.748083
PELI1	235.5176	275.4973	653.3598	640.2723
PELI2	206.3683	263.4255	254.757	254.112
PELI3	911.2892	953.5552	1109.868	1213.285
PEX10	2483.633	2864.691	2323.764	1932.038
PEX12	551.9391	605.5542	784.3496	818.3487
PEX2	1480.13	1529.265	2565.704	2322.789
PHIP	1232.643	1143.322	1591.152	1346.115
PHRF1	9.38039	169.2531	79.23914	0
PIAS1	1288.556	1282.323	1950.149	1780.311
PIAS2	1589.317	1541.655	1550.676	1614.057
PIAS3	955.4349	973.145	1686.548	1570.624
PIAS4	801.2723	712.4353	713.7541	747.4912
PJA1	1240.872	1368.998	1255.313	1125.118
PJA2	4888.603	4629.001	8064.91	7892.247
PLAA	1487.21	1509.543	1485.215	1469.642
PLK1	194.1921	130.8808	70.93438	175.0515
PML	1695.543	1660.481	2135.913	2159.403
POC1B	531.2889	500.0963	544.9764	554.3535
POLH	1043.727	1232.45	1282.573	1219.406
POLI	986.9966	1122.644	1020.864	1120.283
POLK	1435.75	1313.834	1615.097	1331.684
POMP	2475.202	2399.447	2198.217	1789.56
PPIL2	1801.992	2048.423	1936.044	1691.261
PPP1R11	2055.499	2209.223	2131.742	1969.31
PRAME	0	0	0	0
PRAMEF6	0	0	0	0
PRAMEF9	0	0	0	0
PRKAA1	2618.583	2650.598	2429.23	2081.727

PRKAA2	1085.057	1048.711	1162.646	1167.227
PRKN	140.7548	127.4877	288.5342	254.9943
PROKR1	0.500825	0	0	0
PRPF19	7785.859	8490.169	6115.479	5028.772
PRPF8	11719.94	12093.88	13290.34	11568.71
PSMA1	4504.717	4188.118	4249.81	3986.175
PSMA2	3227.301	2976.331	3196.335	3051.202
PSMA3	1981.351	1925.51	1686.885	1566.768
PSMA4	3383.384	3302.061	3027.621	2721.331
PSMA5	3010.255	3009.841	2470.38	2157.151
PSMA6	3125.884	3172.426	2648.754	2383.336
PSMA7	6724.024	6957.368	5079.99	4997.818
PSMA8	0	0	0	0
PSMB1	4418.673	4408.56	4042.67	3471.694
PSMB10	592.9911	630.5048	719.2813	634.1686
PSMB11	0	0.993997	0	0.997871
PSMB2	2998.393	2749.63	2675.741	2394.329
PSMB3	2537.613	2792.505	2888.38	2572.846
PSMB4	6210.213	6580.466	6345.251	6431.784
PSMB5	3688.924	3524.684	4177.921	4086.069
PSMB6	3047.507	2904.581	3252.238	2912.78
PSMB7	4652.836	4620.611	4681.439	4201.011
PSMB8	1743.059	1774.024	2754.643	3016.156
PSMB9	122.8357	106.674	182.6487	240.2504
PSMC1	4715.73	4342.724	4132.908	3550.854
PSMC2	3823.167	3775.207	4257.487	3763.048
PSMC3	4595.582	5289.546	4880.877	3973.74
PSMC4	4809.24	5131.503	3745.396	3388.409
PSMC5	4180.319	4213.023	3799.97	3561.931
PSMC6	2490.281	2173.578	2488.055	2196.696
PSMD1	5343.973	5017.66	5093.625	4128.132
PSMD10	1784.139	1724.745	2150.484	2151.101
PSMD11	5478.301	5030.511	4093.348	3658.396
PSMD12	2530.167	2677.292	1854.612	1510.539
PSMD13	3240.347	3138.027	3051.951	2717.214
PSMD14	3354.454	3487.668	3203.458	2871.411
PSMD2	11574.37	11405.21	12845.08	11200
PSMD3	6220.88	6493.754	5820.508	5217.367
PSMD4	3433.583	3413.68	4285.705	4125.639
PSMD5	1627.979	1559.494	1542.587	1470.031
PSMD6	2488.288	2428.202	2263.375	1973.773
PSMD7	3811.867	3732.273	3111.531	2882.775
PSMD8	6026.029	6550.452	5362.235	4772.063
PSMD9	1992.335	2081.984	1952.592	1883.152
PSME1	3473.701	3204.619	4353.935	4015.752
PSME2	1454.96	1352.486	1592.178	1400.681
PSME3	7506.53	6899.074	5521.103	4711.123
PSME4	3189.538	2823.444	2656.085	2289.198

PSMF1	5934.097	6042.302	5168.482	4779.758
PSMG1	1381.062	1191.439	953.6651	935.6582
PSMG2	1521.989	1457.748	1509.475	1339.134
PSMG3	1142.629	1059.381	667.3252	616.2782
PSMG4	544.5741	514.4527	454.025	428.2073
PWP1	2585.87	2352.673	1866.855	1686.117
RAB40A	29.52229	47.78269	45.96897	49.75506
RAB40AL	0.232846	1.417692	3.007593	0.766457
RAB40B	245.0995	198.9687	245.7548	224.33
RAB40C	1067.297	716.8925	896.0676	853.7508
RAD18	851.5241	758.2078	771.4408	538.8079
RAD23A	6300.539	6375.49	5317.344	5128.944
RAD23B	7467.817	6941.876	7532.086	7163.358
RAG1	44.44186	30.58217	206.9387	220.9349
RANBP10	1280.051	1180.019	1616.309	1746.855
RANBP2	4403.098	3934.13	3736.987	3263.385
RANBP9	1914.088	1879.632	1938.679	1827.92
RAPSN	8.412958	7.903667	1.855952	0.770521
RBBP4	5775.937	5346.76	6081.408	5802.278
RBBP5	1432.373	1239.978	1217.233	1122.502
RBBP6	1955.808	2482.796	1778.405	1884.901
RBBP7	5491.312	5521.508	4961.054	4843.583
RBCK1	5275.582	5522.454	4931.121	4920.076
RBM10	2271.513	2178.603	2465.953	2549.592
RBM44	12.49065	28.86411	6.892474	10.18396
RBM5	3573.886	3810.091	4096.716	3759.115
RBM6	2418.484	2768.217	2858.432	2752.746
RBX1	1376.338	1334.821	1518.405	1367.336
RC3H1	983.2572	1009.602	1274.349	1086.962
RC3H2	3894.458	3468.327	3001.742	2843.113
RCBTB1	3316.411	3867.933	3170.97	3012.042
RCBTB2	358.7216	408.9357	564.2994	781.8196
RCHY1	1115.478	1070.414	1371.046	1218.66
RCOR1	2579.856	1729.39	1636.386	1534.386
REV1	1255.043	1159.673	1213.084	1113.839
RFC1	2049.567	1987.935	3016.128	2801.091
RFFL	899.5389	1147.354	1066.503	951.352
RFPL1	2.32344	2.419817	1.329884	1.512903
RFPL2	0	0	0	0
RFPL3	0	0	0	1.301433
RFPL4A	0	0	0	0
RFPL4AL1	0	0	0	0
RFPL4B	1.250985	2.746335	2.998297	2.245551
RFWD3	1644.612	1562.558	1057.361	982.927
RGS17	114.2892	109.5052	71.84379	48.40297
RHBDD1	1604.358	1670.63	1039.485	1121.318
RHBDD3	1887.568	2127.391	2102.328	1841.706
RHOBTB1	1633.359	1556.674	988.86	953.6012

RHOBTB2	3884.658	4189.965	4261.482	4400.688
RHOBTB3	1871.418	2263.854	3425.586	3750.359
RING1	2116.122	2251.899	1568.529	1587.12
RIOK3	3624.665	4015.536	3389.534	2900.954
RLIM	3413.817	3292.792	3300.137	3186.476
RMND5A	4891.37	4749.439	4199.436	4578.271
RMND5B	2168.585	2133.414	2391.184	2295.899
RNF10	9512.016	10223.73	8787.101	7828.49
RNF103	3398.4	3889.709	3681.986	3498.385
RNF11	6308.209	6198	6481.836	6669.968
RNF111	1574.339	1324.044	1423.412	1324.103
RNF112	4.636613	0.252092	0.756835	5.573405
RNF113A	339.3062	328.3034	452.8565	522.9066
RNF113B	0	0	0	0
RNF114	3029.323	2807.754	3111.834	2978.021
RNF115	2012.483	2074.567	2344.227	2320.298
RNF121	1412.187	1323.513	1280.883	1164.491
RNF122	132.6235	109.0823	330.8203	406.1785
RNF123	2029.718	2481.183	2609.122	2409.121
RNF125	369.7606	318.0382	263.1641	224.4344
RNF126	2116.441	2217.254	1475.561	1258.154
RNF128	135.0814	103.5893	161.411	156.9262
RNF13	6912.49	6474.728	13411.25	12558.85
RNF130	4109.868	3661.356	5717.079	6393.219
RNF133	0	0	0.500601	0.249491
RNF135	952.1575	1052.523	1310.015	1253.022
RNF138	751.6453	702.4307	606.4725	577.7571
RNF139	2204.241	2268.566	2292.364	2068.058
RNF14	2486.888	2424.463	2959.291	2886.759
RNF141	3085.889	3089.829	3124.004	2841.585
RNF144A	362.6559	347.0292	569.2323	532.0859
RNF144B	529.1868	669.264	405.5545	339.6184
RNF145	4403.049	5018.826	4436.185	3688.716
RNF146	1291.52	1383.964	1530.88	1595.518
RNF148	0	0.25095	1.496991	0
RNF149	2726.358	3047.071	1898.155	1780.309
RNF150	1425.434	1150.717	2955.845	2882.676
RNF151	3.976729	2.292786	1.011687	0.510402
RNF152	121.7508	85.32104	175.6705	189.2865
RNF157	319.3584	391.3284	454.9987	553.7309
RNF166	902.111	776.8372	716.7688	678.8843
RNF167	3825.774	3534.947	5027.063	4633.944
RNF168	902.1539	786.5591	679.8372	685.0824
RNF169	1836.362	1825.723	1397.235	1089.6
RNF17	7.211699	3.000034	2.487589	4.715366
RNF170	615.3865	556.8043	878.659	823.115
RNF175	18.29578	21.24679	21.60588	32.03808
RNF180	450.9084	514.1619	1000.31	1046.676

RNF181	1226.262	1371.297	1551.68	1482.793
RNF182	402.9623	311.777	716.8281	654.9885
RNF183	0.77729	4.874291	8.881577	8.22284
RNF185	3970.443	4199.949	4208.052	3667.402
RNF186	0	0	0	0
RNF187	10509.71	9425.89	8698.314	9313.98
RNF19A	1147.234	1174.774	1316.341	1216.18
RNF19B	1053.927	904.1238	928.3149	996.8151
RNF2	791.3065	644.2535	778.9313	725.5349
RNF20	1893.696	2010.511	2796.555	2561.704
RNF207	826.0647	1287.042	1135.956	971.9109
RNF208	2342.626	2232.04	2251.565	2287.43
RNF212	10.11815	0	10.96599	6.003118
RNF212B	0	0	0	0
RNF213	3880.155	3842.538	5067.442	4660.592
RNF214	820.448	840.4062	1017.583	946.9035
RNF215	755.0754	934.5919	1150.632	1119.974
RNF216	3196.966	3281.073	2883.355	2947.358
RNF217	2043.997	1778.386	1329.389	1250.239
RNF220	3887.111	3969.249	3058.5	2873.098
RNF222	1.75141	2.752412	0.748974	0.996688
RNF223	2.999692	0.748361	1.745598	1.247758
RNF224	227.6486	349.3789	188.3896	176.0325
RNF225	1.503238	0	0	0
RNF227	345.5154	314.4715	356.2578	352.58
RNF24	846.5342	503.3766	582.7649	630.727
RNF25	1139.204	1284.936	904.0516	751.7863
RNF26	2923.615	3088.939	3343.246	3308.35
RNF31	2613	2872.419	2112.363	2045.194
RNF32	72.28173	59.31329	71.01071	109.228
RNF34	903.7619	805.2292	781.2943	750.335
RNF38	2075.901	1968.575	2548.238	2610.585
RNF39	26.29377	23.24808	11.46735	15.5943
RNF4	3890.266	3519.877	2716.005	2692.659
RNF40	5721.562	5189.964	5255.593	5017.24
RNF41	3383.841	3425.714	2960.297	3087.244
RNF43	2.079212	1.367867	4.16258	0.512616
RNF44	4031.247	3819.934	3777.64	3506.725
RNF5	2782.619	2502.175	3547.226	3635.63
RNF6	3139.253	3155.952	2370.173	2064.726
RNF7	3198.907	3198.354	2044.772	1958.012
RNF8	864.3299	797.0871	766.115	762.4307
RNFT1	305.7429	316.3704	307.1346	326.0509
RNFT2	205.9079	220.605	419.2842	257.0122
RPS27A	26078.66	22246.76	23418.7	23530.7
RSPRY1	1496.044	1418.587	1700.02	1757.155
RWDD1	1926.427	1823.804	1758.756	1918.844
RWDD2A	593.5343	711.1997	822.149	885.1518

RWDD2B	631.0105	725.993	764.799	733.5697
RWDD3	230.319	311.4794	646.0304	671.4521
RWDD4	1595.32	1548.611	1101.894	1097.63
RYBP	2065.695	2084.638	1634.879	1519.46
SACS	2033.53	1618.284	950.632	801.82
SAE1	3523.706	3417.856	3155.814	3172.008
SDE2	1425.377	1463.818	1069.004	989.0687
SELENOS	1988.824	1951.724	1245.732	1298.619
SEM1	1012.331	891.1229	814.7355	802.7865
SENP1	980.9348	941.9454	734.3212	755.3206
SENP2	1698.391	1786.75	1730.126	1732.357
SENP3	3880.703	3390.262	2810.335	2501.794
SENP5	1440.523	1391.24	1026.584	1120.363
SENP6	4419.485	3781.074	3368.166	3245.17
SENP7	244.9181	257.8323	480.6512	458.5666
SENP8	184.1976	192.2468	169.8937	202.7887
SETDB1	1605.234	1517.828	1397.254	1449.825
SF3A1	5121.1	5499.118	5584.112	5327.104
SF3B3	8899.667	9030.416	7869.536	6094.176
SH3RF1	989.682	852.3198	573.4987	628.8032
SH3RF2	1270.459	1034.806	1353.022	940.2015
SH3RF3	0	0	0	0
SHARPIN	1848.457	2010.849	2362.335	2189.367
SHKBP1	2094.768	2317.843	2733.238	2713.243
SHPRH	1186.515	994.9751	1173.169	1077.73
SIAH1	1309.336	1360.243	1770.103	2103.787
SIAH2	1771.287	1678.738	1646.708	1772.888
SIAH3	0	0.747071	6.465342	4.982681
SIK1	0	0	0	0
SIK2	4202.102	4663.81	5016.477	4618.026
SIK3	1897.971	2039.408	1475.019	1492.254
SKP1	11591.06	11998.02	14323.09	12581.26
SKP2	368.917	381.6624	634.7466	567.4123
SLX4	602.1357	644.4137	823.7628	682.1662
SMARCAD1	2459.126	2400.503	2457.711	2029.808
SMN1	522.4129	396.3485	324.7891	294.2993
SMU1	2982.902	2716.505	2737.893	2445.192
SMURF1	2490.37	2441.945	2141.526	1946.312
SMURF2	1696.762	1579.772	1258.862	1388.383
SNRK	1451.742	1813.886	2103.744	1907.407
SNRNP25	568.8354	528.9771	731.1546	745.5962
SNRNP40	1289.765	1299.695	1203.097	1076.88
SOCS1	19.27676	29.92615	33.48977	31.50107
SOCS2	126.8668	145.0649	169.3738	191.3351
SOCS3	204.9394	264.1477	680.3731	758.6469
SOCS4	1534.787	1406.862	1316.738	1161.685
SOCS5	1339.259	1245.298	1549.979	1303.484
SOCS6	1924.076	1875.068	1337.251	1313.249

SOCS7	1770.684	1476.601	1321.949	1461.191
SPATS2	1592.654	1431.151	1839.755	1673.681
SPATS2L	6402.566	6009.262	10771.3	12471.11
SPOP	1639.527	1510.37	2011.583	2051.41
SPOPL	2280.9	2139.563	1430.435	1527.116
SPRTN	405.2209	426.5855	390.4323	328.1759
SPSB1	1116.813	1059.774	791.4377	800.1034
SPSB2	534.515	459.9299	357.0764	329.4192
SPSB3	1382.239	1566.324	1745.297	1832.336
SPSB4	9.548932	2.945102	6.535212	9.541432
SQSTM1	89968.71	84251.64	43794.16	41091.32
STAMBP	2633.968	2598.021	2695.062	2478.301
STAMBPL1	162.6974	138.69	116.1327	109.3752
STUB1	4595.141	4521.72	4160.082	4320.859
SUMO1	3234.983	3079.497	3116.914	2807.654
SUMO1P1	0	0	0	0
SUMO2	5238.396	4790.473	5458.694	5532.48
SUMO3	5638.886	5318.018	5527.657	5395.56
SUMO4	0.503653	0.249857	0	0.250226
SVIP	922.8256	970.4508	1111.982	986.5102
SYVN1	3286.226	3577.614	2547.414	2679.379
TAB2	3196.278	2938.694	4342.272	4582.461
TAB3	2669.817	2595.128	2390.858	2362.708
TAF15	3266.808	3112.823	3102.574	2836.657
TANK	1502.859	1818.08	1450.416	1542.609
TBCB	2156.247	2314.793	2906.541	2520.645
TBCE	1185.017	1074.4	1154.537	1047.824
TBCEL	1193.983	1146.66	772.7012	834.7124
TBK1	1409.357	1427.443	1362.635	1238.067
TBKBP1	1304.939	1283.792	1350.331	1751.585
TDG	1075.954	1202.149	796.1724	708.6004
TDP2	1445.173	1391.144	1315.391	1326.155
TDRD3	581.0927	542.4941	744.2229	824.2282
TECR	4641.754	5047.522	6518.48	6832.707
TECRL	0	0.460228	0	0
TEX13A	0	0	0	0
TEX13C	0	0	0	0
TEX13D	0	0	0	0
TINCR	10.38781	3.352984	0.18528	0.372127
TLE1	3393.889	2832.437	3613.995	3334.647
TLE2	289.819	222.1243	282.9002	378.7808
TLE3	706.2207	452.4473	1141.829	1071.967
TMEM129	5333.645	5714.067	6769.239	6804.94
TMEM183A	3185.096	3193.187	3450.946	3423.275
TMUB1	1747.701	1771.16	2473.915	2528.61
TMUB2	1758.405	1899.61	1984.826	1933.056
TNFAIP1	5208.209	4771.44	3844.511	3381.707
TNFAIP3	292.1576	590.0045	228.3527	182.9784

TNIP1	3942.708	4497.228	5618.092	5474.162
TNIP2	1792.565	1764.324	1217.922	1157.577
TNIP3	0	0.577153	0.577971	1.326002
TNK1	596.5541	619.8589	725.465	755.773
TNK2	2293.814	1891.707	2272.345	2398.455
TNRC6C	550.214	621.028	706.999	755.7071
TOPORS	1433.64	1428.696	1393.435	1302.636
TOR1AIP2	2056.975	2073.01	2346.441	2046.448
TRAF1	402.0297	403.8237	363.327	318.1929
TRAF2	723.891	553.0511	517.3714	451.4501
TRAF3	4913.848	4396.611	2679.699	2391.672
TRAF3IP2	1289.513	1824.798	1587.806	1174.046
TRAF4	3845.946	3465.64	2077.018	1952.008
TRAF5	770.3194	833.4224	694.8288	688.6956
TRAF6	1059.663	1131.753	1330.877	1171.012
TRAF7	6525.62	6741.488	6758.292	6709.326
TRAIP	100.2515	92.67103	93.57086	109.5434
TRIM10	0	0	0	0
TRIM11	2074.169	1896.223	1244.28	1148.724
TRIM13	1395.892	1270.218	1192.198	1191.656
TRIM14	1426.965	1370.992	1034.786	972.2581
TRIM15	0	0	0	0
TRIM16	2066.385	1587.59	1478.85	1872.189
TRIM16L	2071.664	1511.516	1836.05	2771.715
TRIM17	47.21667	70.09363	33.556	49.65608
TRIM2	1738.026	1934.434	2396.606	2185.9
TRIM21	455.3794	393.2222	484.3098	438.8094
TRIM22	2256.9	2270.903	3804.311	3365.742
TRIM23	1010.138	1045.297	998.3139	1042.536
TRIM24	984.8356	839.1799	1124.84	1021.913
TRIM25	3482.813	4779.776	2494.165	2339.862
TRIM26	911.4711	848.6433	798.9477	1044.364
TRIM27	4465.511	3957.515	3036.92	2747.667
TRIM28	14028.68	14620.47	12770.92	11975.72
TRIM29	6.257212	10.01627	6.500387	6.500977
TRIM3	516.1968	573.6649	692.8715	623.8634
TRIM31	0	0	0	0
TRIM32	1376.512	1356.875	1415.992	1230.505
TRIM33	2338.342	2349.524	2003.278	1835.179
TRIM34	58.44516	55.35159	330.2741	274.9681
TRIM35	1501.185	1272.602	1076.956	975.5711
TRIM36	130.6672	90.66725	171.1365	174.0484
TRIM37	2476.476	2309.2	2719.347	2568.207
TRIM38	1047.114	1356.109	1075.641	1006.274
TRIM39	1051.246	1044.647	963.3259	958.7618
TRIM4	2139.874	1960.573	2097.267	2034.124
TRIM40	0	0	0	0
TRIM41	1520.983	1702.025	1762.707	1494.96

TRIM42	0	0	0	0.248874
TRIM43	0	0	0	0
TRIM43B	0	0	0	0
TRIM44	8314.866	7527.22	5228.308	4360.713
TRIM45	315.8459	378.5499	500.8738	512.159
TRIM46	390.7528	290.157	369.481	321.1745
TRIM47	5469.221	3969.179	4057.924	3213.956
TRIM48	0	0	0	0
TRIM49	0	0	0	0
TRIM49B	0.500803	0	0	0
TRIM49C	0	0	0	0
TRIM49D1	3.994773	1.250088	0.747549	1.249414
TRIM5	602.4815	656.0031	1424.467	1195.008
TRIM50	0	0.740783	0	0
TRIM51	0	0.250412	0	0
TRIM52	687.1423	715.3742	868.7162	842.0472
TRIM54	6.636855	6.863376	0.255523	0
TRIM55	1.471893	0	0.976477	0.566235
TRIM56	4575.248	5143.56	4050.859	3892.142
TRIM58	1240.82	1040.305	940.7799	914.4717
TRIM59	256.6795	231.5332	255.5964	261.9434
TRIM6	63.03485	74.70521	223.1397	263.9991
TRIM60	0	0	0	0
TRIM61	15.23338	8.278168	3.089238	1.330657
TRIM62	421.2583	426.9017	406.3039	353.2313
TRIM63	0	0	0	0
TRIM64	0	0	0	0
TRIM64B	0	0.497279	0	0
TRIM64C	0	0	0	0
TRIM65	1604.613	1160.788	1366.801	1278.886
TRIM66	588.0656	696.2752	693.3712	611.6191
TRIM67	30.6715	41.15184	5.96033	11.45235
TRIM68	536.2776	578.0422	789.6796	811.1689
TRIM69	7.094025	11.87417	33.83119	28.89629
TRIM7	37.35134	29.36229	19.15661	21.19392
TRIM71	1.25164	1.744825	2.252741	0.746941
TRIM72	6.821896	5.566703	7.741876	5.794977
TRIM73	18.09087	25.0026	22.65959	22.48441
TRIM74	63.41912	73.32569	73.42152	59.81419
TRIM75	0	0	0	0.24952
TRIM77	0	0	0	0
TRIM8	5557.383	5737.15	6381.323	7201.13
TRIM9	163.1022	147.1951	105.3551	128.3439
TRIML1	0.742128	0	0	0
TRIML2	1.398188	2.959597	3.101433	1.79533
TRIP12	6693.058	6327.556	5476.803	5142.106
TRIP4	693.0218	735.198	593.3906	607.5506
TRPC4AP	5298.105	5324.215	5994.902	5663.238

TSFM	1243.955	1391.707	1569.123	1244.135
TTC3	13090.31	11971.22	16510.71	17559.97
TULP4	3682.884	3341.594	4166.157	3939.198
TXNIP	425.7033	263.3914	1446.367	2387.947
TXNL1	2805.597	2342.292	3076.853	3164.68
UBA1	12946.02	13609.36	18889.79	17127.73
UBA2	4424.703	4180.586	3216.19	3103.417
UBA3	1404.768	1527.486	1818.399	1793.392
UBA5	1444.671	1636.09	1821.228	1853.467
UBA52	17040.8	15639.32	17018.81	16638.18
UBA6	1585.886	1461.162	1299.131	1078.754
UBA7	264.9132	230.3663	396.1378	502.5084
UBAC1	1559.268	1494.891	2174.126	2230.528
UBAC2	2458.943	2353.763	3026.043	2921.695
UBALD1	2218.61	2196.633	1358.694	1129.683
UBALD2	933.3858	967.0687	1021.839	1191.476
UBAP1L	666.9579	764.8016	1034.235	822.1312
UBAP2	2359.215	2077.925	1900.892	1713.27
UBAP2L	7964.972	8365.318	7360.697	6409.039
UBASH3A	0	0	2.568112	0
UBASH3B	1408.836	1389.615	451.9745	407.6415
UBB	23508.02	24885.65	26226.18	27574.1
UBC	45659.38	55649.89	59925.75	59022.53
UBE2A	2575.381	2261.758	2238.366	2265.013
UBE2B	1880.871	1616.07	1704.262	1613.069
UBE2C	79.70527	66.80439	31.91204	105.9502
UBE2D1	1136.531	963.073	894.2672	786.5955
UBE2D2	5230.55	4619.248	3600.096	3638.288
UBE2D3	12463.3	11043.55	9876.109	9465.555
UBE2D4	1042.381	1096.344	1175.111	1137.043
UBE2DNL	0	0	0	0
UBE2E1	2547.814	2387.415	2436.487	2104.348
UBE2E2	1336.475	1424.748	1250.477	1336.614
UBE2E3	4943.141	4537.891	7042.373	7107.671
UBE2F	1425.846	1290.644	1071.877	1111.841
UBE2G1	2436.423	2176.973	2409.736	2383.112
UBE2G2	5146.937	5193.456	3384.876	3136.242
UBE2H	5037.549	4511.897	5839.429	5915.948
UBE2I	4573.189	4221.331	3392.387	3226.162
UBE2J1	4683.127	4375.449	4321.35	4590.612
UBE2J2	2661.399	2643.566	2086.815	2084.804
UBE2K	3493.357	3306.129	2909.309	2728.53
UBE2L3	4239.931	4289.027	4150.814	3946.874
UBE2L5	0.250462	0	0.999573	0.498038
UBE2L6	1306.489	1143.042	1940.613	2300.607
UBE2M	2897.408	2971.458	2335.446	2328.533
UBE2N	3540.854	3519.906	2388.458	2118.979
UBE2NL	0	0	0	0

UBE2O	2302.702	1983.237	1899.099	1828.429
UBE2Q1	3914.431	3995.422	4073.024	4091.198
UBE2Q2	2495.42	2368.034	2219.098	2304.575
UBE2QL1	66.44938	39.93621	70.72023	103.5012
UBE2R2	3657.801	3660.615	4037.232	4442.041
UBE2S	1885.179	1925.792	1011.572	1099.956
UBE2T	53.43396	51.01298	116.0148	95.93576
UBE2U	5.168084	4.873688	6.779683	7.453693
UBE2V1	4555.345	4330.983	4531.407	4706.828
UBE2V2	1824.315	1801.61	1546.586	1485.454
UBE2W	1386.609	1332.956	1049.569	1065.431
UBE2Z	8141.459	7860.309	5671.28	5448.749
UBE3A	3786.142	3106.862	2600.597	2623.823
UBE3B	2610.992	2894.052	3310.226	3293.762
UBE3C	7871.074	7125.917	6176.163	5844.702
UBE3D	145.3563	91.56778	213.0743	191.7583
UBE4A	3242.185	3383.633	3478.276	3094.897
UBE4B	4602.798	4640.88	6563.521	6022.59
UBFD1	5596.277	5633.196	5013.95	4698.33
UBL3	2884.313	2344.525	4639.261	4680.029
UBL4A	3086.652	2604.38	2934.084	2729.684
UBL4B	0	0	0	0
UBL5	1729.709	1687.786	2291.102	2063.194
UBL7	1458.84	1421.55	2153.698	2139.384
UBLCP1	1266.147	1183.747	1094.322	938.507
UBOX5	793.6533	698.8573	763.8083	781.1091
UBQLN1	7188.177	6188.073	5547.774	5080.768
UBQLN2	2758.406	2680.727	3252.61	3111.892
UBQLN3	0	0	0	0
UBQLN4	4394.675	4067.192	3232.755	2819.981
UBQLNL	5.237076	7.729964	11.47671	18.47307
UBR1	1304.222	1273.037	1605.85	1474.208
UBR2	2373.014	2080.757	2156.493	2009.144
UBR3	2124.401	2048.598	2423.293	2238.159
UBR4	5862.02	5785.686	5978.159	5536.959
UBR5	2919.334	2777.669	4191.796	3906.813
UBR7	1249.356	1223.714	1762.269	1697.59
UBTD1	1964.065	1731.02	2433.087	2580.07
UBTD2	1379.25	1088.049	1280.784	1342.58
UBXN1	4431.654	4278.786	4200.993	4713.28
UBXN10	1.005303	4.269695	0.755244	2.514715
UBXN11	1215.298	1313.369	1434.791	1146.452
UBXN2A	825.0715	820.88	1050.938	1006.443
UBXN2B	1973.807	1882.179	2101.695	1945.188
UBXN4	9982.355	8998.754	12771.81	12381.33
UBXN6	7732.869	8300.855	9387.877	9399.121
UBXN7	1746.195	1444.958	1909.802	1745.485
UBXN8	559.5759	541.32	512.9235	379.6082

UCHL1	10300.97	8652.004	9172.936	8441.05
UCHL3	627.084	580.3543	423.8289	404.7752
UCHL5	1039.383	888.3057	977.0026	915.4932
UFC1	2200.071	1989.245	2582.765	2798.335
UFD1	1377.755	1438.39	1316.325	1187.2
UFL1	1628.245	1680.216	1739.424	1505.894
UFM1	3455.584	3534.793	2702.219	2694.148
UFSP1	292.6243	291.5836	138.1795	108.5999
UFSP2	1053.441	926.0214	1360.911	1381.136
UHRF1	227.1985	141.7145	189.3924	189.8034
UHRF2	1730.415	1244.648	1209.191	1179.315
UIMC1	966.4136	936.5983	809.5637	688.4538
UNK	3959.942	3677.295	4571.264	4032.296
UNKL	982.5052	1153.912	916.3655	858.5175
URM1	2911.758	2996.701	2883.645	2491.003
USP1	1973.595	1661.889	1427.224	1460.927
USP10	4325.873	3996.346	3016.781	2542.638
USP11	3290.572	3859.934	5044.124	4997.313
USP12	3216.533	2326.397	2118.941	2131.365
USP13	974.2337	908.512	868.6446	771.7189
USP14	3987.967	3172.827	2466.116	2119.023
USP15	1706.809	1539.12	1263.764	995.7805
USP16	1519.669	1574.281	1336.3	1219.223
USP17L2	5.985417	7.00133	2.244797	4.242343
USP18	15.45865	19.67468	77.99932	57.37001
USP19	4020.628	4427.896	4934.09	4617.738
USP2	535.2985	556.6882	1425.388	1581.005
USP20	1616.755	1561.383	1826.161	1727.155
USP21	1157.81	1042.495	1622.269	1601.951
USP22	18083.73	17327.91	18540.36	17755.24
USP24	2843.294	2698.189	2419.464	2181.707
USP25	3696.776	3501.238	3430.421	3275.088
USP26	0	0	0	0
USP27X	348.4217	295.4968	290.1113	301.5828
USP28	819.0874	731.8039	1027.386	1051.228
USP29	0	0	0	0
USP3	1243.695	1101.779	1395.023	1296.283
USP30	680.5545	798.8772	1581.955	1676.528
USP31	1810.033	1278.15	622.7716	720.0444
USP32	4998.307	4268.153	5256.703	4531.297
USP33	3563.309	3687.359	5803.41	5900.363
USP34	5396.102	5005.924	7084.305	6412.76
USP35	723.7194	860.4468	1028.687	954.624
USP36	4534.512	4115.215	2266.446	2055.029
USP37	676.4314	511.4665	617.7319	594.2595
USP38	2352.474	2258.619	2009.026	1970.163
USP39	2136.079	2135.604	2064.369	2109.706
USP4	2579.301	2665.58	2891.435	2626.893

USP40	3239.279	3644.005	3473.164	3357.163
USP42	1123.915	1120.48	873.3623	868.9918
USP43	233.7212	197.2945	288.5501	186.5098
USP44	240.9937	228.7137	185.9081	191.9309
USP45	1047.255	842.9884	635.2664	657.5422
USP46	2455.688	2457.721	2119.785	2130.563
USP47	3119.974	2935.518	3090.511	2958.871
USP48	2600.556	2500.511	3448.892	3457.165
USP49	257.7989	242.2263	267.2389	286.8808
USP5	3345.65	3408.635	3732.312	3253.795
USP50	11.27158	24.19206	12.59797	12.47945
USP51	340.8245	374.2698	489.6025	523.4195
USP53	2099.384	1957.486	1321.719	1311.506
USP54	1648.027	1752.852	3123.596	2724.784
USP6	3.793323	2.069754	1.988754	4.344867
USP7	5514.442	4741.756	3933.503	3583.313
USP8	2695.742	2693.742	2686.228	2432.44
USP9X	5469.863	5064.144	5536.695	5236.996
USP9Y	361.1604	532.5101	572.95	486.4136
USPL1	1119.94	961.9053	643.8358	629.5045
VCP	19158.96	20552.96	19925.28	17552.16
VCPIP1	1189.994	1166.023	1044.58	983.0162
VHL	2072.886	2009.289	1739.604	1755.4
VHLL	4.798365	0.250336	0	0
VPS11	2716.414	2850.703	3752.004	3367.309
VPS41	4819.338	4877.459	5601.338	5248.641
VPS8	1101.099	1164.771	1459.693	1417.223
WDR12	2604.923	2563.518	1850.446	1545.402
WDR26	3544.538	3672.332	3581.224	3428.043
WDR48	1944.056	1637.963	1921.139	1935.889
WDR5	3399.735	2869.441	2056.677	1892.728
WDR53	313.8836	242.4255	267.0281	227.131
WDR59	1941.667	1980.225	1872.002	1789.616
WDR5B	449.5613	452.8944	391.6581	408.7392
WDR76	132.2397	116.41	253.2562	290.291
WDR82	6715.424	6594.295	6815.384	6260.993
WDSUB1	497.0727	540.2629	700.735	752.4132
WDTC1	4112.302	4400.872	5469.297	5764.217
WRNIP1	3411.494	3508.049	3235.184	3352.029
WSB1	2977.959	3780.414	3516.011	4479.461
WSB2	7830.119	7819.986	6483.072	6480.709
WWP1	2473.467	2372.749	2834.847	2846.561
WWP2	3146.505	3647.821	3425.451	3162.131
XIAP	2153.434	2004.683	1899.249	1834.362
XRCC4	154.5335	190.6671	190.297	221.6378
YAF2	580.1101	642.6421	545.0778	500.6998
YAP1	11566.22	10842.12	9423.48	9123.481
YOD1	1915.357	1990.177	1839.333	1386.573

YPEL5	3569.948	3755.642	4138.636	4206.353
ZBED1	1997.132	2420.16	2115.427	1852.975
ZC3H12A	510.1941	727.7895	501.9273	466.2179
ZC3H12B	84.20518	88.11693	117.2397	148.7855
ZC3H12C	1141.737	1279.624	973.1756	889.1917
ZC3H12D	6.245476	4.280232	3.987302	11.14887
ZCCHC12	2.518461	4.958152	0.74085	1.000645
ZER1	2903.143	3320.493	4359.186	4222.542
ZFAND2A	679.0005	711.7091	283.6937	318.3299
ZFAND2B	516.3889	435.6435	625.2185	695.7218
ZFAND3	6875.714	7677.892	5197.339	5080.727
ZFAND4	312.7511	330.5257	340.2705	341.7588
ZFAND5	8270.605	9179.387	15908.89	19260.66
ZFAND6	3220.109	3261.815	3162.14	3001.3
ZFP91	4991.089	4622.937	4167.635	3828.812
ZMYM2	3368.686	3167.788	3782.233	3869.334
ZMYM3	910.262	930.8518	2542.868	2442.157
ZNF451	1388.326	1328.424	1257.103	1296.644
ZNF598	3304.629	3115.804	1849.252	1659.769
ZNRF1	2406.966	2430.155	1671.395	1255.751
ZNRF2	496.2661	568.0376	645.7996	791.1443
ZNRF3	1309.402	1352.147	1209.687	1082.691
ZNRF4	0	0	0	0
ZRANB1	1881.789	1824.751	1838.795	1918.496
ZRANB2	2822.112	2621.265	2682.088	2425.015
ZRANB3	171.5521	206.6999	274.8307	240.6972
ZSWIM2	0	0	0	0
ZSWIM8	4777.188	6200.946	5238.01	5178.735
ZUP1	355.935	344.2854	259.0497	256.6541
ZYG11A	7.384381	5.922979	2.520344	3.948872
ZYG11B	2067.998	2254.817	3679.173	3295.154

Supplementary Table S7: Chaperone-mediated pathway genes

Gene	POS (4Hrs)	POS (6Hrs)	POS (24Hrs)	POS (48Hrs)
A2M	3.881319	1.029871	5.50226	5.81591
AAAS	1224.136	1134.66	1899.03	1758.673
AARS1	16647.83	18043.92	10105.84	11938.96
AARS2	1197.079	1118.157	1565.897	1130.316
AARSD1	813.9666	726.1803	726.6535	632.3414
AATF	3568.161	3428.947	2440.455	2304.731
ABCE1	6460.057	5295.418	3264.687	2649.082
ABCF2	4559.559	4364.318	2320.609	2268.913
AFG3L2	6358.172	5753.874	5226.316	4859.459
AGK	1196.303	1214.401	1225.227	1132.529
AGR2	0	0	0	0
AGR3	0	0	0	0
AHCTF1	3000.104	2554.06	2023.377	1849.876
AHSA1	4144.61	3889.635	3195.228	2858.189
AIMP1	1915.593	1761.615	1411.184	1284.499
AIMP2	1775.59	2047.877	1244.537	1192.559
AIP	1713.571	1758.782	2366.991	2640.049
AIPL1	1.250871	1.559054	1.754181	0.498798
AMFR	7752.562	8256.74	8327.31	8511.277
ANKZF1	1011.813	1193.538	1342.793	1427.996
ANP32E	1544.561	1123.535	1939.824	1743.795
APCS	0	0	0	0
APOO	410.0881	401.7548	712.0065	637.4198
APOOL	2143.844	2104.85	1780.135	1547.316
ARCN1	6406.321	6149.444	6039.904	5813.977
ARF1	13300.26	13066.9	15433.21	14911.75
ASCC1	985.0738	861.5672	1358.816	1330.411
ASCC2	2465.725	2455.959	2335.113	2085.668
ASCC3	2640.222	2922.316	3087.904	2745.524
ASF1A	718.7095	584.799	899.2281	939.5362
ASF1B	145.7736	55.9448	86.91072	151.1372
ATAD1	3541.981	3820.402	3843.517	3722.231
ATF3	1717.747	1817.997	336.7189	383.6697
ATF4	26102.14	25247.02	8625.732	10732.82
ATF6	4640.39	4967.294	4709.253	3787.912
ATP13A1	5991.198	6733.157	6226.312	4824.003
ATP23	103.176	112.1979	239.145	285.8804
ATRX	2247.737	2308.635	3148.997	2828.248
AUP1	5333.068	5295.691	4455.035	4011.471
BAG1	7046.34	7321.246	6300.993	6341.863
BAG2	5209.773	3942.933	2306.177	1853.081
BAG3	3370.14	3061.08	2380.384	2252.977
BAG4	877.5457	916.4137	1001.65	1024.578
BAG5	3581.782	3305.383	2690.493	2421.404

BAG6	6408.107	7162.851	8472.846	7431.858
BBS10	992.6096	1095.142	934.1575	895.8881
BBS12	366.1755	405.0791	500.773	547.9376
BCAP31	8032.497	8416.523	10609.04	9083.838
BCCIP	2134.289	1801.149	1489.514	1341.511
BCS1L	1154.455	1123.604	1112.412	984.4226
BMS1	2718.657	2434.878	1898.379	1751.608
BOP1	2949.25	2991.609	1920.469	1662.391
BSG	72793.95	74304.81	91566.86	91475.51
BUD23	2776.916	2882.875	2422.655	2113.296
BYSL	1655.361	1641.329	861.5066	721.4623
BZW1	12238.39	11736.49	7821.482	6934.76
BZW2	3150.163	2478.51	3272.916	2665.064
C1R	33490.4	46813.41	48731.6	48666.7
C1S	22757.14	30812.35	29855.99	32972.54
CABIN1	2442.577	2321.823	3324.632	3019.572
CACYBP	2104.702	1790.379	2418.869	2330.233
CALR	34404.74	34526.84	41032	37692.97
CALR3	1.502007	0.748684	0.998595	0.249957
CAMLG	1724.737	1580.187	2352.258	2616.536
CANX	7727.381	8115.966	9292.236	7408.185
CARS1	6189.402	5909.189	2613.083	2559.593
CARS2	2341.613	2548.435	2497.618	2265.963
CASQ1	4.992477	5.234631	12.2348	8.241733
CASQ2	0	0	0	0.748797
CCDC47	8296.527	7978.209	8007.286	7223.718
CCT2	11608.2	10806.62	6767.124	5586.682
CCT3	12721.18	12330.15	10954.76	9257.16
CCT4	7640.388	6491.011	6544.216	5923.213
CCT5	11671.34	10961.39	6936.246	5569.505
CCT6A	10205.7	8984.203	6619.932	5872.149
CCT6B	66.81804	60.35633	84.23216	87.81756
CCT7	11031.95	11186.07	10439.68	8624.433
CCT8	7827.966	7217.424	6946.434	6471.136
CCT8L2	0	1.499354	0	1.247618
CDC37	6469.001	6859.49	6196.963	4996.337
CDC37L1	874.852	813.7295	815.3804	886.8426
CDK5RAP3	5648.75	5944.391	6039.642	5735.219
CHAF1A	502.9875	570.7174	754.768	639.3309
CHAF1B	236.0004	226.0392	251.9322	196.4142
CHCHD3	3675.58	3780.993	3451.102	3057.525
CHCHD4	913.5935	794.3363	684.9968	635.9898
CHCHD6	526.2723	636.9344	750.5359	861.3402
CHORDC1	1795.752	1300.967	798.7603	755.1393
CLGN	1637.094	1019.559	1389.23	1841.94
CLPB	1058.959	1113.563	1436.244	1233.472
CLPP	2237.849	2451.185	2537.634	2347.177
CLPX	2240.489	2152.937	2684.537	2309.261

CLU	90034.06	137947.3	152940.3	162298.5
CNOT4	817.8269	845.701	903.8483	908.5761
COLGALT1	5813.929	5178.318	3613.953	3087.678
COLGALT2	367.855	382.0264	617.4028	660.2893
COPA	9301.935	9260.083	11931.29	10567.22
COPB1	4992.799	5028.568	7285.756	6292.568
COPB2	5637.755	5865.03	6545.392	5675.107
COPE	5269.39	5987.329	7005.668	5943.244
COPG1	6910.309	7756.218	8169.37	7405.952
COPG2	1373.869	1408.528	2286.496	2055.055
COPZ1	4902.249	4896.69	6346.583	5711.88
COPZ2	316.4055	344.4874	397.0042	499.5968
CRNKL1	1388.454	1331.111	1279.344	1142.003
CRTAP	32208.68	28397.69	43326.57	42427.64
CRYAA	0	0	0	0
CRYAB	50665.64	52457.78	61851.09	51789.06
CSE1L	3653.207	3217.165	3275.915	2778.281
CSN2	0	0.744819	0.497577	0
CSNK2B	4530.498	4616.499	4367.391	4354.806
CWC27	651.7584	624.8234	751.7715	714.476
CYB5R4	602.3679	684.4092	662.2159	534.4781
DAD1	3865.286	3757.686	4773.453	3958.219
DARS1	3806.819	3586.987	4687.653	4653.282
DARS2	1297.514	1208.627	1930.682	1449.607
DAXX	2613.193	2188.076	2153.887	1985.464
DDIT3	4519.358	4794.042	1442.876	1870.893
DDOST	11261.46	11112.03	14572.04	13491.41
DDRGK1	2463.25	2626.62	2556.776	2454.062
DDX19B	1655.189	1958.413	1286.753	1256.169
DDX21	11090.49	9103	3325.282	2779.787
DDX27	2323.488	2351.763	1562.603	1332.004
DDX28	1264.309	1321.246	860.6142	795.0965
DDX3X	17090.69	16574.04	13884.58	13553.62
DDX3Y	742.4677	911.9405	811.7031	728.7865
DELE1	3900.422	4607.213	5089.818	4703.993
DENR	3334.706	3062.525	2234.835	2194.311
DERL1	3013.299	3055.423	2782.962	2697.231
DERL2	2129.676	1939.123	1608.24	1579.206
DERL3	41.05264	27.1371	67.64648	39.25805
DHX15	6906.159	7071.868	5076.556	4368.922
DHX29	4995.675	5398.453	3949.243	2535.918
DHX30	6880.959	7752.168	6939.177	6367.863
DIMT1	1646.545	1417.427	887.4034	731.2755
DIS3	2451.616	2390.917	1935.418	1672.086
DKC1	5145.151	4449.401	2099.188	1827.942
DNAAF11	83.12678	106.932	216.3142	191.115
DNAAF4	91.8802	109.8371	142.8098	96.02648
DNAAF6	0	0	0.74819	0.24894

DNAJA1	11653.65	9408.121	5767.207	5430.218
DNAJA2	4317.943	4063.89	4040.332	3959.495
DNAJA3	5517.901	5476.485	3324.401	3119.148
DNAJA4	83.98124	87.65548	64.34086	109.3658
DNAJB1	7247.679	5636.149	5681.728	5513.025
DNAJB11	1948.167	2107.781	1614.859	1493.946
DNAJB12	3804.363	4160.44	3472.49	3200.142
DNAJB13	2.357633	0.342879	2.080067	7.066216
DNAJB14	2036.619	2111.499	2496.102	2046.402
DNAJB2	3891.654	3949.412	4557.088	4607.639
DNAJB4	657.6538	648.4393	722.3272	760.8399
DNAJB5	677.0374	723.122	647.4539	707.8809
DNAJB6	6636.806	6295.145	5236.964	4924.523
DNAJB7	2.312331	2.558486	1.059374	0.913161
DNAJB8	0	0	0	0
DNAJB9	1799.608	1798.116	1823.307	2035.149
DNAJC1	1732.583	1741.865	1453.678	1840.946
DNAJC10	6214.185	6449.065	6251.045	5129.681
DNAJC11	4020.039	3961.728	3186.938	2757.421
DNAJC12	0	0.702552	0	0.75967
DNAJC13	3125.078	2999.678	3260.428	2980.448
DNAJC14	2018.176	1789.116	2076.061	1969.083
DNAJC15	1356.991	1389.821	1680.919	1551.176
DNAJC16	1522.118	1554.462	1514.688	1374.654
DNAJC17	434.6524	388.6649	354.2892	261.0635
DNAJC18	1138.562	1201.508	1365.798	1381.825
DNAJC19	834.4284	854.9903	912.7221	889.9078
DNAJC2	1376.225	1158.193	683.555	582.1887
DNAJC21	2525.447	2073.589	2452.678	2341.518
DNAJC22	625.7846	670.3774	914.6076	811.1943
DNAJC24	805.9057	730.487	902.7767	848.4236
DNAJC25	639.8928	671.7121	498.2207	462.2313
DNAJC25-GNG10	13.19778	8.817258	12.75627	4.865483
DNAJC27	453.6483	465.5508	446.1709	458.6479
DNAJC28	45.45837	38.51464	58.91012	84.64222
DNAJC3	3075.647	3055.653	4113.355	4160.758
DNAJC30	1297.185	1393.819	1263.82	1284.808
DNAJC4	1708.416	1674.367	1912.56	2422.977
DNAJC5	5745.202	5890.922	5445.645	5418.841
DNAJC5B	0	0	2.29397	0
DNAJC5G	0	0	0	0
DNAJC6	560.6126	416.3281	385.4322	368.2822
DNAJC7	2969.196	2751.127	2566.298	2355.633
DNAJC8	4215.511	3850.822	4400.969	3946.128
DNAJC9	939.0769	802.5421	880.7703	800.673
DRG2	1597.981	1751.177	1440.09	1323.074
DTD1	1469.209	1546.707	1382.114	1214.398

DTD2	1083.084	963.4605	970.1915	842.8959
DUSP12	722.0619	739.2363	441.7009	469.2011
EARS2	2160.228	2337.734	1443.762	1172.077
EDEM1	2431.09	2049.797	1472.124	1408.359
EDEM2	1047.228	1052.4	1821.846	1668.056
EDEM3	1267.418	1080.968	1127.669	1133.962
EDF1	5614.814	5257.984	4709.875	4584.295
EEF1A1	696529.5	611117.2	703618.4	710491.6
EEF1A2	1.24834	0.249935	14.00779	0.999675
EEF1B2	19477.07	15102.8	16581.29	16251.9
EEF1D	14636.99	12905.69	14143.66	14543.62
EEF1E1	907.1334	790.7962	490.3958	434.3271
EEF1G	88283.08	68135.33	84185.85	88752.06
EEF2	204261.2	169776.1	194184.6	224578.4
EEF2K	1068.737	1193.668	2640.047	2485.629
EEF2KMT	1616.304	1560.46	871.7204	745.391
EEFSEC	715.9096	919.1641	708.9756	696.9565
EFL1	1358.159	1365.141	1428.164	1318.155
EIF1	25143.61	21709.11	13947.64	16211.85
EIF1AX	5895.926	5912.677	4511.982	3857.602
EIF1AY	211.0231	253.9131	238.7066	214.0155
EIF2A	3300.312	3386.69	4123.673	4042.909
EIF2AK1	5716.091	5537.435	7413.607	7131.326
EIF2AK2	2232.493	2012.856	2432.145	2229.629
EIF2AK3	2016.237	1594.862	1663.332	1541.413
EIF2AK4	2039.111	2037.207	2380.711	2416.402
EIF2B1	2068.183	2049.549	2019.162	1701.16
EIF2B2	1386.193	1511.638	1280.9	1155.808
EIF2B3	827.8036	848.7725	725.9117	600.8251
EIF2B4	1654.369	1563.609	1634.183	1549.281
EIF2B5	2831.015	2886.331	2804.592	2490.46
EIF2D	2589.912	2149.2	2820.642	2933.915
EIF2S1	4093.54	3486.955	2129.793	1925.031
EIF2S2	7533.47	6972.935	3353.646	3606.091
EIF2S3	10501.43	9572.898	10855.18	10190.17
EIF2S3B	0	0.282385	0	0
EIF3A	18841.18	16352.67	14106.38	12439.15
EIF3B	14533.04	14131.94	8080.713	6945.455
EIF3C	20927.52	20262.97	20196.21	16605.17
EIF3D	10107.22	8950.387	9227.537	9901.92
EIF3E	12360.2	9964.714	9965.492	11712.93
EIF3F	10239.59	9265.923	11256.64	12840.37
EIF3G	6281.521	6077.632	5087.088	5606.529
EIF3H	8064.316	6977.161	7764.916	8355.23
EIF3I	7513.061	7614.677	7918.288	6897.971
EIF3J	3938.917	3010.896	1712.337	1613.963
EIF3K	6187.11	6153.518	8074.076	8197.845
EIF3L	20007.23	16359.29	19125.14	20967.28

EIF3M	5426.672	4943.239	3756.337	3859.291
EIF4A1	29970.95	27832.61	17887.54	15078.73
EIF4A2	14869.93	11772.66	18618.35	20038.22
EIF4B	31335.51	25217.38	34019.54	38350.3
EIF4E	4343.05	3878.917	2743.868	2409.855
EIF4E2	2511.787	2327.203	2099.429	1960.714
EIF4E3	1389.922	1437.677	1718.967	1570.761
EIF4EBP1	5425.731	5459.228	1246.578	1298.809
EIF4EBP2	9207.469	9997.013	12578.49	12620.2
EIF4EBP3	1148.762	1043.832	1139.268	1002.581
EIF4G1	27911.75	28419.49	19971.55	17322.65
EIF4G2	47554.79	44529.13	46205.6	43939.33
EIF4G3	5517.888	5961.139	6198.325	5559.324
EIF4H	20525.39	19716.85	16781.95	16126.64
EIF5	11130.72	10900.94	10357.88	9967.665
EIF5A	17583.89	17631.46	13247.9	9908.738
EIF5A2	1112.331	883.6279	955.8839	935.6222
EIF5AL1	4.48708	6.129437	3.492504	1.994746
EIF5B	4983.947	4638.48	3280.136	2832.412
EIF6	5584.335	6124.395	4378.079	3861.225
ELAC2	3530.758	3600.878	3596.328	3093.249
EMB	2417.96	1858.462	2228.156	1744.023
EMC1	4360.545	4296.587	3517.27	3208.03
EMC10	10417.9	11639.33	13753.44	14320.45
EMC2	849.3347	885.6766	1489.643	1372.448
EMC3	2212.702	2499.572	2598.667	2487.994
EMC4	2543.322	2494.129	2408.372	2227.729
EMC6	1486.178	1488.899	1235.115	1227.99
EMC7	2610.298	2449.011	2298.026	2146.657
EMC8	1848.513	1949.849	1422.104	1285.059
EMC9	909.9347	975.8977	1024.516	1064.014
EMG1	2265.392	2133.681	1471.369	1201.835
ENGASE	1376.879	1413.459	1698.959	1767.726
EPRS1	10499.46	9883.279	6584.439	6295.242
ERAL1	1983.72	2098.463	2001.304	1693.481
ERI1	576.009	397.384	361.6738	359.459
ERLEC1	2623.232	2869.601	3872.665	3525.55
ERLIN1	2176.353	2016.568	2386.75	2062.598
ERLIN2	4475.957	4417.78	6882.996	6519.172
ERN1	706.5068	796.7532	485.9544	481.9902
ERO1A	4389.112	4080.344	5001.196	4445.824
ERO1B	363.4885	365.8365	367.395	346.088
ERP27	0	0	0	0
ERP29	7113.876	6555.822	7915.292	8325.562
ERP44	1855.412	1983.204	2105.323	2123.845
ETF1	9058.565	7777.173	4558.872	4130.162
EXOSC10	2535.106	2654.493	2388.214	2341.159
F2	2.253514	0.497938	0	0

FAF2	3457.584	3642.418	3332.849	2926.445
FAM8A1	2436.324	2530.272	3088.612	2996.661
FARS2	387.5806	508.7291	852.741	742.4938
FARSA	4939.564	5056.297	3447.107	3035.156
FARSB	2691.896	2359.012	1524.783	1209.795
FASTKD2	2402.792	2349.541	2029.675	1733.904
FAU	8909.983	7992.319	7882.524	7797.947
FBL	2668.272	2261.308	2138.29	2200.392
FCF1	1583.756	1592.652	1222.857	1162.61
FICD	1860.046	2295.269	1028.935	1092.731
FKBP10	3634.752	3281.899	4772.52	4504.517
FKBP11	374.3242	268.9152	313.2481	274.6005
FKBP14	424.3626	444.4384	482.7708	495.2104
FKBP15	1995.274	2071.374	2172.585	1959.904
FKBP1A	8319.229	7682.685	9197.009	7254.95
FKBP1B	143.5575	148.8423	196.4283	211.6316
FKBP2	1442.302	1589.383	1940.062	1690.024
FKBP3	1151.269	1105.281	1434.414	1292.613
FKBP4	8257.962	6795.384	4500.817	4243.767
FKBP5	461.7213	296.7039	217.9577	171.3747
FKBP6	24.84648	27.60381	18.7423	16.81968
FKBP7	110.8956	88.61834	158.8685	211.0015
FKBP8	12567.93	12324.68	15046.16	15655.5
FKBP9	5569.61	5773.691	7555.873	8185.707
FKBPL	139.4015	137.6573	234.0524	217.3974
FNIP1	1710.126	2030.901	2267.076	1921.83
FNIP2	3760.055	3096.12	2852.232	2675.331
GADD45GIP1	2843.286	3129.127	2826.824	2561.398
GAK	4412.062	4816.597	4016.335	3682.294
GANAB	16209.09	14988.84	16876.02	14911.17
GAR1	952.8484	848.2992	577.8798	515.8906
GARS1	17040.03	16436.26	4764.373	5486.278
GATB	941.9479	936.6081	1555.577	1381.547
GATC	2856.038	2443.469	1644.83	1477.592
GCN1	7920.841	8185.716	6791.785	5813.292
GET1	1638.672	1602.436	2517.476	2449.199
GET3	3963.043	4084.845	5062.723	4296.33
GET4	2171.851	2088.472	1920.366	1817.394
GFER	1434.346	1379.707	1320.474	1398.427
GFM1	3832.504	3912.146	3569.285	2948.843
GFM2	1731.168	1789.798	1985.209	1871.05
GIGYF1	5111.05	5386.398	4191.174	3693.408
GIGYF2	4072.887	3864.444	3981.412	3802.109
GLE1	1689.332	1854.733	2202.312	1848.096
GNL2	2269.986	1855.13	1293.271	1217.703
GRPEL1	1819.857	1718.365	1076.047	1077.071
GRPEL2	867.5772	872.218	589.9694	514.545
GSPT1	8501.397	7447.021	5133.855	4479.607

GSPT2	679.2848	707.9686	847.3118	750.3534
GTPBP1	4570.815	4441.939	4245.153	4554.015
GTPBP10	818.4596	761.9266	653.3567	648.5002
GTPBP2	5741.936	6832.516	1923.796	2032.459
GTPBP4	3926.477	3722.685	1716.171	1476.626
GTPBP6	3267.413	3606.906	2806.813	2497.35
GUF1	1821.785	1819.577	1467.77	1430.605
HACD3	6516.727	6050.99	7126.822	7303.121
HAPSTR1	4946.31	4156.823	3280.015	3160.792
HAPSTR2	1.006022	0	0	0.749692
HARS1	3198.051	3320.083	2297.245	2227.569
HARS2	1411.87	1331.721	1435.642	1408.145
HAT1	477.6722	535.0235	400.7614	367.0779
HBS1L	2136.944	1986.288	1945.843	1881.562
HEATR1	4560.724	4010.521	2524.006	1940.995
HEATR3	1001.869	912.0187	862.7746	649.4124
HERPUD1	4292.851	4269.254	6324.654	6597.141
HERPUD2	1419.374	1620.809	1546.552	1427.975
HIF1A	10405.99	11396.25	8536.65	10314.76
HIKESHI	764.5608	777.6542	838.9886	875.3619
HIRA	4112.102	3642.205	2643.859	2527.093
HJURP	184.3094	109.6696	57.21465	112.4615
HP	0	0	0.999953	0
HSCB	291.969	329.9108	299.3917	325.011
HSD17B10	1667.299	1751.078	1878.509	1691.936
HSF1	3891.152	3607.502	3488.547	3805.074
HSF2	523.6281	574.7481	795.7948	751.2037
HSF4	785.7156	784.4484	1129.742	1497.838
HSF5	0	0.502129	0	0.499787
HSP90AA1	56446.76	51539.81	47440.52	37673.46
HSP90AB1	59794.97	59324.43	54864.52	47704.46
HSP90B1	22156.81	22294.86	29763.93	19809.14
HSPA12A	1270.175	1096.118	818.5907	649.2793
HSPA12B	7.152228	8.683397	2.041365	4.853542
HSPA13	3077.871	3298.131	1934.678	1806.646
HSPA14	553.4552	454.859	333.9145	281.9943
HSPA1A	10768.61	9448.769	6583.007	7042.784
HSPA1B	11300.89	6277.058	5356.385	6157.636
HSPA1L	107.4679	88.64344	134.4287	159.9115
HSPA2	1434.69	1330.092	2197.422	1996.199
HSPA4	8771.544	8374.842	6831.357	5664.479
HSPA4L	1386.066	1476.136	1963.556	1766.376
HSPA5	33652.39	33375.93	19145.63	21266.71
HSPA6	29.16467	27.96877	53.16321	72.10697
HSPA8	71575.72	71334.57	57925.72	50703.72
HSPA9	34483.43	35322.06	16897.86	15582.22
HSPB1	15222.7	16173.08	20750.61	17546.11
HSPB2	465.2636	444.9757	635.3897	719.1399

HSPB3	74.53699	116.9925	88.69706	47.98601
HSPB6	39.79157	26.20503	44.23767	44.4613
HSPB7	44.29586	45.29799	33.63365	45.05063
HSPB8	13506.8	9977.2	14500.05	14852.44
HSPB9	3.552232	4.405758	1.250099	6.551713
HSPBAP1	345.0731	312.7906	548.2906	483.6903
HSPBP1	3121.747	3439.323	3110.905	2427.007
HSPD1	26573.1	23019.56	18825.54	16331.15
HSPE1	2555.95	2209.031	2404.204	2098.196
HSPE1-MOB4	13.04834	4.205535	30.39763	10.83097
HSPH1	8218.018	5789.736	4011.517	3642.775
HTRA2	1437.641	1510.206	1241.294	1254.779
HUWE1	9002.564	8086.092	9109.357	8000.746
HYOU1	10617.77	11243.81	5630.104	6052.559
IARS1	24892.79	25226.47	9827.787	10518.14
IARS2	5804.869	5739.521	8550.429	6970.576
IDE	3059.777	3184.946	3174.184	2480.513
ILF2	6421.244	5791.791	5305.228	4668.13
ILF3	15363.68	15360.41	12846.75	10561.15
IMMP1L	90.18311	100.9569	88.8829	92.66381
IMMP2L	753.0267	800.7032	1159.195	1020.694
IMMT	4617.466	4394.001	5118.652	4711.862
IMP3	3162.709	3535.015	3324.261	3052.718
IMP4	3714.684	3815.494	2428.031	2043.677
IPO11	1254.287	1190.63	1065.304	889.4633
IPO13	3141.241	3408.924	3788.733	4058.39
IPO4	4913.895	5349.156	3236.807	2458.747
IPO5	12146.54	11748.54	10325.92	9874.594
IPO7	13444.68	11740.14	10705.37	9842.504
IPO8	2215.705	2012.131	2415.695	2003.671
IPO9	4743.27	4934.883	4957.654	4463.834
ISG20L2	1984.108	1781.79	1245.709	1182.6
ITGB1BP2	6.435812	5.106585	1.049596	1.697116
ITM2B	38017.63	39505.68	48370.14	54499.87
JKAMP	2202.376	2107.644	2594.802	2395.2
KARS1	5934.884	5422.598	5411.621	5040.104
KDELR1	8835.924	8468.144	11458.57	11036.06
KDELR2	9359.388	9444.071	9444.988	9218.047
KDELR3	234.9132	195.4794	395.6427	373.7579
KEAP1	5136.063	4650.983	5046.455	5396.198
KPNA1	4252.764	4126.199	3331.937	2974.983
KPNA2	2788.242	2355.01	1936.153	1866.869
KPNA3	2654.002	2092.276	2039.532	1947.257
KPNA4	6655.562	5690.786	3860.406	3746.501
KPNA5	679.3699	598.739	676.1132	560.4777
KPNA6	4550.932	4549.06	4331.598	4048.631
KPNA7	5.297035	1.701138	0.250094	2.898117
KPNB1	15317.96	14746.12	10566.19	9526.307

KRTCAP2	1823.913	1660.139	2058.93	1886.503
LARS1	7997.771	7701.192	6770.737	5889.147
LARS2	1912.608	1920.348	2268.029	1970.003
LLPH	1501.845	1517.427	1004.456	870.4161
LMAN1	6547.894	5519.576	7321.735	6779.023
LMAN1L	0	0	0.745215	0.993816
LMAN2	6624.479	7020.368	8946.732	8230.306
LMAN2L	1361.959	1398.549	2036.305	1837.922
LOC124902573	0	0	0	0.745334
LONP1	14087.81	15267.9	7628.463	8191.724
LRPPRC	9616.606	8924.252	10875.83	9450.908
LSG1	2087.969	2139.592	1490.825	1265.069
LTN1	1521.07	1415.003	1246.438	1143.944
LTV1	1375.946	1303.181	869.1518	678.7855
MAGT1	2907.888	2902.97	3295.877	2834.373
MALSU1	1275.252	1486.426	1413.806	1278.006
MAN1B1	3256.322	3063.477	5277.429	4939.953
MAN2C1	3636.008	3987.294	4247.111	3414.522
MANF	1687.689	1557.125	1158.812	1282.715
MAP3K20	3223.313	2535.423	3685.012	3979.259
MARCHF5	2643.716	2468.517	2637.706	2506.459
MARCHF6	6167.944	5975.232	6998.451	7081.247
MARS1	12858.88	14093.15	4701.943	5162.269
MARS2	984.5374	977.7252	556.4248	470.9794
MCM2	1373.702	1178.127	1830.737	1431.512
MCTS1	902.5533	961.5278	1028.564	979.4379
METAP1D	591.1175	652.8322	628.5676	510.9399
METTL15	1010.444	943.0361	867.905	744.8614
MICOS10	2331.929	2383.416	2261.113	1913.952
MICOS13	1341.517	1308.846	1526.73	1372.007
MIEF1	3796.887	3860.152	3193.162	2746.127
MIF	22048.55	24602.96	30320.01	29471.16
MIPEP	1068.553	1153.758	1812.903	1489.75
MIR124-1	0	0	0	0
MIR124-2	0	0	0	0
MIR124-3	0	0	0	0
MIR224	0	0	0	0
MIR24-1	0	0	0	0
MIR24-2	0	0	0	0
MIR27B	0	0	0	0
MIR488	0	0	0	0
MKKS	1187.283	1253.661	1584.857	1500.728
MKRN1	3219.614	3381.704	3850.541	3830.964
MKRN2	1910.669	1601.403	1968.379	1703.298
MLEC	10397.2	9903.453	11545.2	10797.02
MME	884.903	784.2679	591.4582	374.3012
MMGT1	1755.143	1543.212	1304.319	1295.055
MOGS	3555.154	3278.816	3209.285	2986.515

MPHOSPH10	1006.657	1032.087	909.1944	801.1616
MPV17L2	888.7491	856.9412	570.458	490.5898
MRM1	414.517	490.444	373.5278	312.6846
MRM2	1114.359	1071.049	868.1429	787.3203
MRM3	804.0326	790.1513	739.4874	662.7771
MRPL1	559.8979	459.4087	515.4631	500.8867
MRPL10	1705.794	1688.025	2144.699	2092.34
MRPL11	1071.806	934.4312	1039.322	914.9933
MRPL12	3861.04	4057.334	3463.793	3053.094
MRPL13	815.0037	813.9567	822.3751	781.7705
MRPL14	1431.614	1311.158	1201.174	1120.074
MRPL15	2029.666	1870.979	2323.871	2091.062
MRPL16	895.0256	886.7545	1487.71	1465.771
MRPL17	1621.287	1492.61	1612.659	1492.608
MRPL18	1651.256	1575.402	1530.787	1444.752
MRPL19	2159.733	1813.683	1927.688	1648.906
MRPL2	910.8543	952.3024	1091.891	937.8091
MRPL20	1898.498	1893.79	1522.204	1439.782
MRPL21	1231.269	1104.135	1048.982	874.4123
MRPL22	645.0378	578.937	684.8905	595.9098
MRPL23	1574.389	1588.495	1792.056	1673.743
MRPL24	1360.408	1304.265	1706.254	1296.74
MRPL27	1190.079	1210.84	1314.557	1054.458
MRPL28	2034.534	2203.382	2074.581	1917.505
MRPL3	4383.545	4220.154	3189.173	2703.639
MRPL30	1835.069	1661.138	1811.871	1626.856
MRPL32	1034.077	1047.257	955.4237	912.5052
MRPL33	449.4545	392.6399	379.1543	371.5722
MRPL34	2750.556	2604.52	4432.77	4350.964
MRPL35	1204.013	1208.338	1561.896	1326.002
MRPL36	871.6904	892.8197	731.8138	651.3004
MRPL37	4147.927	3931.282	4189.556	4128.567
MRPL38	2656.167	2834.679	2501.168	2391.632
MRPL39	516.8959	504.2739	519.357	464.5032
MRPL4	3997.06	3985.241	2738.162	2383.069
MRPL40	1148.863	1056.101	1282.309	1246.346
MRPL41	1808.944	1581.319	2003.609	1990.357
MRPL42	2164.013	1942.073	2032.551	1812.345
MRPL43	2067.127	2046.988	3068.367	2987.369
MRPL44	1292.732	1375.578	1716.598	1660.598
MRPL45	2028.383	1736.695	2059.043	2311.507
MRPL46	687.9536	713.1052	747.4119	673.3173
MRPL47	1095.232	1061.032	1040.518	888.7699
MRPL48	657.3989	579.8561	802.0473	754.1927
MRPL49	4926.15	4398.744	4331.921	4072.984
MRPL50	873.7878	786.2641	656.0918	599.1036
MRPL51	1711.804	1557.775	1626.294	1372.673
MRPL52	1001.195	947.6323	944.7651	904.6344

MRPL53	1247.805	1223.829	1308.223	1330.621
MRPL54	866.438	939.5762	823.2687	647.5444
MRPL55	932.3222	940.4612	827.835	836.1424
MRPL57	2106.727	2225.008	2409.035	2368.335
MRPL58	760.4185	709.2666	884.863	789.4518
MRPL9	1420.29	1376.812	1401.87	1267.756
MRPS10	2220.607	1930.64	1715.991	1588.929
MRPS11	1255.819	1369.962	1439.971	1385.627
MRPS12	2100.485	2271.343	1768.032	1530.617
MRPS14	1035.055	1029.079	1242.306	1150.552
MRPS15	1951.164	1818.078	2048.448	1956.932
MRPS16	5176.89	5668.071	4868.761	4461.779
MRPS17	766.0361	559.9104	473.7636	477.1763
MRPS18A	1620.743	1538.711	2108.573	2015.116
MRPS18B	2241.855	2098.566	3112.749	3024.346
MRPS18C	600.3301	546.5629	500.7945	461.6484
MRPS2	3207.603	3126.643	2941.908	2874.75
MRPS21	2222.804	2115.733	2328.016	2272.727
MRPS22	1036.78	933.4061	936.4487	844.317
MRPS23	2159.574	2056.106	1528.651	1411.13
MRPS24	1949.931	1796.175	2251.889	2149.996
MRPS25	3791.774	3913.39	4029.715	3360.809
MRPS26	2319.289	2395.556	1907.002	1905.404
MRPS27	3739.127	3194.511	4703.723	4661.59
MRPS28	741.7617	793.4427	915.8801	741.0948
MRPS30	1533.191	1604.199	1411.939	1256.404
MRPS31	422.0584	375.0476	434.4437	439.3269
MRPS33	1108.568	1018.014	1313.678	1290.336
MRPS34	1990.67	1809.361	2117.42	2090.166
MRPS35	3052.728	2828.471	2854.258	2418.809
MRPS5	1943.569	1650.964	1903.96	1760.118
MRPS6	4070.885	4468.065	9606.88	8769.126
MRPS7	2671.883	2705.761	2816.632	2578.22
MRPS9	990.2949	913.9646	1065.179	977.5572
MRRF	1518.044	1610.743	1641.043	1122.878
MRTO4	3617.806	3508.757	1702.792	1286.654
MTCH1	11584.46	12535.3	11606.54	11690.88
MTCH2	3610.624	3522.051	4368.311	3871.676
MTERF3	565.8766	502.6941	440.4275	467.1643
MTERF4	1181.013	1241.23	1315.256	1307.926
MTG1	1428.025	1602.904	1660.077	1359.642
MTG2	1482.6	1493.408	1117.743	1012.751
MTIF2	1656.135	1460.928	1816.962	1712.317
MTIF3	601.9566	593.6324	1159.352	1046.035
MTRES1	345.5266	346.786	527.8298	515.3348
MTRF1	276.2611	300.0281	440.811	420.5813
MTRF1L	2007.062	2101.361	1599.122	1410.511
MTRFR	585.8576	520.654	639.3049	688.5386

MTX1	1915.608	1876.937	2048.629	1809.485
MTX2	1197.101	1140.299	1321.67	1243.808
MTX3	1158.164	1157.197	918.5709	862.9279
MYDGF	3141.666	2969.593	2873.422	2602.143
MZB1	0	0.861641	0	0.27591
NAP1L1	26453.81	23283.75	21012.78	23683.33
NAP1L2	31.6773	40.49177	143.009	111.2677
NAP1L3	286.0434	346.9859	837.9906	770.3606
NAP1L4	4412.92	4243.288	6653.049	6518.09
NAP1L5	295.6416	312.5748	558.5116	677.6305
NARS1	15390.65	15927.71	10814.29	9572.488
NARS2	1440.821	1357.346	2248.074	2172.547
NASP	1081.35	1058.138	1160.327	1052.033
NAT10	3513.174	3219.501	2405.077	2023.133
NCL	27483.07	23732.95	18077.59	14741.67
NCLN	6615.157	7580.918	5005.668	3660.364
NDC1	1683.41	1746.958	1747.219	1364.544
NDUFAB1	1832.465	1863.056	2101.077	1915.943
NEMF	1770.13	1742.659	1631.022	1411.91
NFE2L2	7336.62	8271.734	6841.443	7166.456
NFKB1	1965.088	1705.816	1679.382	1769.792
NFKB2	2522.499	2521.727	1204.376	1040.12
NGDN	854.496	875.8195	692.4108	634.2936
NGLY1	2286.947	2023.229	2413.534	2219.789
NGRN	7871.572	7723.88	6582.001	6509.941
NHP2	3407.51	3178.522	2820.092	2120.461
NKRF	928.5269	882.0041	730.1554	667.8424
NKTR	2442.711	2626.06	2107.182	1656.287
NLN	2134.458	1592.637	1191.169	970.5404
NMD3	2932.293	2736.256	3534.818	3122.177
NOA1	1737.951	1647.713	2321.438	2385.384
NOB1	3800.166	3204.471	2826.948	2879.031
NOC4L	1365.277	1425.524	887.1396	711.955
NOL10	1784.145	1508.639	1078.834	1022.884
NOL11	1971.781	1744.783	1432.384	1288.14
NOL6	5778.65	5934.219	2574.135	2037.726
NOMO1	7327.226	7182.542	7951.7	7538.952
NOMO2	5729.618	5471.203	5635.321	5511.805
NOMO3	5.388653	26.00511	75.305	7.285656
NOP10	1342.948	1183.861	1107.571	1027.12
NOP14	3857.567	3844.142	2423.458	2052.188
NOP2	2943.233	2646.288	1300.779	906.4187
NOP53	15242.88	12455.3	13400.88	15630.31
NOP56	7147.762	5938.595	3043.285	2518.186
NOP58	2909.208	2508.168	1426.348	1049.144
NPLOC4	9135.516	9543.676	9703.509	9523.56
NPM1	28763.88	23075.44	23861.88	24460
NPM2	127.5209	139.0898	153.4887	172.9817

NPM3	877.5771	856.0425	627.3775	534.4734
NSUN4	1408.76	1251.3	1097.554	1119.243
NSUN5	1616.484	1600.365	1131.138	898.1147
NUDC	4718.551	4657.67	3449.791	3208.32
NUDCD1	983.6572	876.342	588.6974	560.0861
NUDCD2	911.1768	833.0268	877.7424	871.306
NUDCD3	3945.031	4083.462	4041.749	3739.41
NUP107	1490.187	1425.771	1241.269	1028.435
NUP133	2122.282	2262.652	2542.366	2302.038
NUP153	2878.85	2583.893	2159.775	1952.763
NUP155	1767.399	1437.756	1349.473	1078.426
NUP160	2220.574	1995.155	1677.129	1451.775
NUP188	4978.25	4917.088	2749.79	2480.138
NUP205	2218.531	2138.331	2154.168	1713.421
NUP210	3410.769	3170.493	2399.874	2232.577
NUP214	2536.79	2659.362	2146.86	2097.538
NUP35	641.831	539.5096	324.6193	273.2637
NUP37	370.5409	387.0807	587.4515	552.0006
NUP42	896.6206	658.3303	638.3599	615.7405
NUP43	1415.847	1449.195	1620.506	1274.091
NUP50	2306.446	2026.844	1939.942	1796.607
NUP54	1021.417	1000.778	889.6216	831.6189
NUP58	2896.015	2514.23	1687.916	1580.277
NUP62	3417.799	3013.377	3081.679	2700.889
NUP62CL	17.51154	16.34631	11.9446	13.21993
NUP85	1288.545	1188.834	1180.629	1025.149
NUP88	2211.462	2156.103	1516.024	1352.875
NUP93	1973.104	1905.376	1939.367	1801.406
NUP98	4806.217	4504.142	3198.537	2905.986
ODF1	0	0	0	0
OGT	7063.571	6844.758	5542.551	5136.2
OMA1	366.6601	359.7468	953.1382	808.7107
OS9	11730.46	12944.41	15480.82	16193.99
OSTC	2143.769	2170.11	2299.85	1994.504
OTUB1	4868.033	5213.025	4695.062	4225.486
OTUD3	668.5741	634.4171	461.0976	457.1672
OXA1L	6400.158	6195.634	8636.556	9218.928
P3H1	1931.84	2184.07	2784.935	2606.319
P3H2	1702.073	1475.856	1393.291	1427.17
P3H3	5248.241	5297.609	6905.424	7298.39
P4HA1	2858.949	2790.331	5592.277	5728.969
P4HA2	1604.549	1387.788	2889.363	3108.741
P4HA3	81.34592	82.86246	100.7422	152.0373
P4HB	25709.25	27552.6	35444.81	34048.2
PABPC1	54987.77	45118.28	56352.37	66505.63
PABPC1L	1910.486	2360.561	1192.358	929.2656
PABPC1L2A	0	0	0	0
PABPC3	0.249583	0	0.248811	0

PABPC4	9514.333	8929.029	9202.983	8779.14
PABPC4L	127.473	108.0671	114.0956	129.9512
PABPC5	10.2378	7.248869	18.23273	12.97006
PAM16	462.4264	505.1922	368.2621	280.2752
PARK7	5740.107	5428.121	6990.051	5926.409
PARN	1497.237	1636.384	1807.51	1762.708
PARS2	546.7509	500.5211	419.938	423.7585
PCSK9	0	3.408994	3.30235	2.824331
PDC	0	0.497878	0.251124	0.501243
PDCL	613.4141	692.0208	890.6114	751.2593
PDCL2	1.755152	0	0	0
PDCL3	718.6236	735.8274	651.3637	495.2809
PDF	1562.228	1664.61	788.2459	718.6194
PDIA2	27.2185	18.70926	10.73816	11.73767
PDIA3	15184.29	15331.02	18787.28	16540.68
PDIA4	5402.312	5192.293	7532.837	6012.004
PDIA5	844.4152	680.1521	1042.667	1002.432
PDIA6	10097.93	9319.451	12210.75	10661.25
PDILT	0	0	0	0
PDRG1	954.5344	959.7795	679.511	695.3356
PELO	1866.366	1936.953	1384.286	1350.074
PELP1	3807.046	3870.481	3142.54	2663.582
PES1	5659.982	5922.47	3469.018	2919.278
PFDN1	2061.945	2057.18	2103.595	2049.477
PFDN2	1114.42	980.6257	727.4869	660.0905
PFDN4	523.9166	526.3629	448.4154	470.2725
PFDN5	5167.211	4507.25	5739.324	5990.492
PFDN6	929.1581	895.8985	721.6633	572.6929
PGGHG	1134.764	925.9702	869.703	946.7973
PHB1	6980.177	6841.935	8602.747	6337.053
PHB2	8973.175	8377.966	8259.544	8106.611
PIH1D1	1556.961	1705.798	1637.493	1645.846
PIH1D2	36.74842	36.67032	68.76979	50.41105
PITRM1	4206.311	4288.778	4900.917	4404.19
PLOD1	4761.588	5021.667	7281.425	7465.202
PLOD2	7493.904	5900.327	9594.879	9677.445
PLOD3	3193.973	3103.748	3314.546	3018.802
PMPCA	3768.529	3664.994	2991.56	2634.159
PMPCB	3313.184	3107.312	3797.602	3779.087
PNO1	2291.081	2024.134	927.4836	795.795
POM121	8551.465	8732.622	5979.182	5318.874
POM121C	6487.601	6921.83	4941.97	4516.509
POM121L12	0	0	0	0
POM121L2	0.993652	0.499161	0.745009	0.249285
POMT1	1923.895	2024.576	2252.907	2200.755
POMT2	3214.634	3382.291	3424.249	2878.38
PPIA	24929.45	22042.24	28111.01	24044
PPIAL4A	0	0	0.252832	0

PPIAL4C	0	0	0	0.253361
PPIAL4D	0	0	0	0
PPIAL4E	0	0	0	0
PPIAL4F	0	0	0	0
PPIAL4G	4.547446	0.249942	1.013422	3.504566
PPIB	10317.47	10342.31	10573.52	9592.746
PPIC	1188.421	1208.683	2033.14	1950.942
PPID	996.5973	807.9067	962.1728	916.7646
PPIE	1751.546	1481.319	1626.291	1496.024
PPIF	4478.996	4588.238	3699.034	3541.895
PPIG	2126.655	2288.37	2236.633	1946.298
PPIH	335.1386	307.9279	408.7347	312.3053
PPIL1	1960.35	2045.285	1437.8	1358.914
PPIL2	1801.992	2048.423	1936.044	1691.261
PPIL3	434.4819	362.329	584.563	596.8161
PPIL4	871.0578	765.883	684.1142	649.7628
PPIL6	312.7321	296.9517	202.7413	232.5018
PPP1R15A	5132.343	6673.708	1764.21	1485.495
PPP1R15B	4593.678	4862.612	3006.948	2607.748
PPP5C	2804.762	3027.736	3167.573	2933.552
PPWD1	627.2065	613.4331	609.0806	597.2687
PRDX1	11069.62	10982.32	15486.62	12942.92
PRDX3	9384.654	8563.896	13077.1	11607.42
PRDX4	2767.386	2656.931	3475.66	3219.516
PRKCSH	11870.93	12100.78	15455.12	14094.89
PRORP	877.9915	972.6767	1328.56	1111.401
PSTK	174.7271	185.5432	170.4936	168.7577
PTCD1	1341.658	1190.393	1110.184	983.8071
PTCD3	3198.797	3040.493	3988.22	3217.534
PTGES3	10615.64	9330.604	7547.353	7041.112
PWP2	0	0	3.834276	0.450242
PZP	0	0	0	0.202568
QARS1	7983.555	7122.114	11198.74	11717.33
QRSL1	1100.419	967.6602	1058.06	1076.587
RACK1	59552.76	48445.83	53401.12	57401.6
RAE1	1024.988	1104.346	1025.444	867.7379
RAN	15919.85	15091.3	10054.57	8706.619
RANBP17	243.0973	302.059	300.9574	205.7271
RANBP2	4403.098	3934.13	3736.987	3263.385
RANBP3	3053.74	3225.966	3176.489	3188.158
RANBP3L	2.213228	1.981239	13.97014	22.29882
RANBP6	1333.444	1321.869	1508.134	1294.954
RANGAP1	9437.775	10643.32	5961.528	5356.861
RARS1	3378.48	3056.98	2933.477	2550.953
RARS2	1474.144	1511.315	1528.772	1350.783
RBBP4	5775.937	5346.76	6081.408	5802.278
RBBP7	5491.312	5521.508	4961.054	4843.583
RBFA	526.98	488.5878	559.981	510.8648

RCC1	1736.292	1610.83	1618.125	1381.746
RCC1L	3755.745	4143.741	3909.05	3611.899
RCL1	1063.782	1041.462	1052.666	892.8834
REL	545.5924	482.9164	491.5327	487.731
RELA	5122.565	5263.214	3969.881	3612.959
RELB	1400.848	1453.801	303.0654	249.3726
REXO2	1654.886	1520.727	1440.705	1527.496
RGPD1	25.51486	11.90618	9.120256	6.971493
RGPD2	6.722297	4.521802	5.90743	0.697528
RGPD3	74.84717	78.85302	86.85811	65.80463
RGPD4	38.94156	47.60912	22.69049	47.14775
RGPD5	1457.566	779.0608	861.7412	1357.98
RGPD6	916.8287	1354.331	1030.491	532.9333
RGPD8	995.4285	1158.254	899.5781	799.6035
RHBDD1	1604.358	1670.63	1039.485	1121.318
RHBDD2	6772.52	7851.843	5475.718	5634.001
RHBDL3	3.929927	5.674601	3.452136	6.467745
RIC3	116.6948	163.5539	107.7285	105.3179
RIOK1	1018.47	803.6978	507.7636	447.4888
RIOK2	850.7592	778.6449	706.6061	704.6583
RIOK3	3624.665	4015.536	3389.534	2900.954
RMRP	1.066691	0	2.175449	0.737245
RNF103	3398.4	3889.709	3681.986	3498.385
RNF139	2204.241	2268.566	2292.364	2068.058
RNF14	2486.888	2424.463	2959.291	2886.759
RNF145	4403.049	5018.826	4436.185	3688.716
RNF170	615.3865	556.8043	878.659	823.115
RNF185	3970.443	4199.949	4208.052	3667.402
RNF25	1139.204	1284.936	904.0516	751.7863
RNF5	2782.619	2502.175	3547.226	3635.63
RNFT1	305.7429	316.3704	307.1346	326.0509
RPAP3	1136.156	1004.629	921.2563	841.5135
RPF2	2481.24	2002.51	1242.369	1142.918
RPL10	56420.3	49222.65	55887.53	55839.44
RPL10A	27021.08	23339.43	26560.34	26149.57
RPL10L	0	0	0	0
RPL11	22238.05	17246.64	20591.24	20548.58
RPL12	35091.48	27708.09	30739.24	31704.37
RPL13	83562.13	70017.65	77161.39	81832.29
RPL13A	100086.1	81877.63	96529.77	101767.4
RPL14	27877.34	24766.96	24658.88	24874.09
RPL15	61617.8	53077.28	57179.39	59578.83
RPL17	32095.94	27621.5	28169.14	28865.79
RPL18	27635.67	23676.69	24362.51	26117.54
RPL18A	49928.01	39905.87	47277.78	51932.36
RPL19	42010.76	35125.06	41161.87	40086.62
RPL21	22145.04	17996.73	20278.81	20231.08
RPL22	20631.6	16654.56	18652.59	18547.35

RPL23	23688.57	21938.34	21833.64	22109.14
RPL23A	29371.14	23539.96	25768.1	25968.28
RPL24	14211.44	12576.15	12755.06	13074.15
RPL26	23881.57	21101.71	21840.62	22122.79
RPL26L1	1149.873	1291.214	923.1753	759.4795
RPL27	19048.08	16158.75	17688.02	17508.3
RPL27A	31351.89	25945.02	29050.8	29395.85
RPL28	29122.99	28218.94	28697.23	31511.09
RPL29	38027.04	29574.32	35337.95	36510.74
RPL3	112041.9	89278.78	110252.1	112268.4
RPL30	14310.23	11588.79	12809.32	13598.26
RPL31	28332.23	23342.91	24234.13	25476.67
RPL32	22843.93	18754.03	21287.36	21780.5
RPL34	15537.13	12799.91	14921.48	15017.47
RPL35	15197.56	12238.82	13453.97	12516.09
RPL35A	11555.31	9335.467	9930.159	10361.41
RPL36	11199.87	8837.857	10062.45	9664.632
RPL36A	11938.33	8121.859	9309.717	9088.187
RPL36AL	3592.051	3175.034	3071.498	3103.194
RPL37	14047.64	11151.12	11236.26	11619.77
RPL37A	17942.99	16186.55	15669.52	16378.67
RPL38	3320.813	2896.618	2802.151	2862.662
RPL39	10619.14	7468.103	8754.948	8630.307
RPL39L	127.5334	133.6932	155.909	171.1901
RPL3L	17.85685	37.01542	50.75325	91.3414
RPL4	81399.5	64268.94	72116.46	74923.03
RPL41	40505.82	32295.1	41058.45	42379.73
RPL5	44444.99	35726.78	36920.73	39402.14
RPL6	42592.39	36827.51	41650.41	42296.96
RPL7	42604.31	34426.72	39060.39	39309.38
RPL7A	59514.3	49759.82	60497.84	59734.32
RPL7L1	5634.05	4582.258	3844.055	3539.306
RPL8	62428.36	52016.21	58600.3	56332.86
RPL9	35803.14	30697.33	31773.24	31584.77
RPLP0	89899.25	71449.11	92512.81	92422.64
RPLP1	58425.36	48675.49	57776.87	60782.14
RPLP2	20965.44	15318.05	18121.35	18025.22
RPN1	11282.76	12123.82	11547.96	10160.27
RPN2	13408.95	14633.92	17846.08	17239.56
RPS10	28000.37	22610.65	26668.05	26399.71
RPS11	34511.06	27441.86	32301.39	33709.87
RPS12	38254.51	31092.2	35857.24	37127.76
RPS13	12381.15	9970.594	10690.22	10413.59
RPS14	33007.86	28002.67	31629.61	32171.14
RPS15	23836.25	21047.34	23441.21	23155.97
RPS15A	13382.82	12893.8	12526.39	13070.03
RPS16	25902.46	22193.19	23549.56	25341.1
RPS17	23662.51	18813.96	21030.84	21021.11

RPS18	43864.19	32761.42	39561.75	41666.07
RPS19	32900.62	26652.95	30361.64	31835.41
RPS2	99788.29	73998.53	87012.99	86938.46
RPS20	24297.35	22356.63	23885.91	25128.5
RPS21	6514.962	5339.243	6078.803	6164.081
RPS23	26781.25	22519.03	24544.84	25097.94
RPS24	22644.31	17574.57	21164.25	21227.41
RPS25	17718.48	13980.1	16346.81	17055.77
RPS26	17492.29	14598.24	16753.17	17103.18
RPS27	16387.85	11952.74	13110.46	13260.59
RPS27A	26078.66	22246.76	23418.7	23530.7
RPS27L	5188.609	5507.68	4558.911	3551.989
RPS28	15196.24	14991.53	16239.25	17878.12
RPS29	2866.709	3123.856	2848.78	3043.705
RPS3	40759.7	31586.88	36620.81	37246.98
RPS3A	54309.84	47700.42	50765.42	51950.04
RPS4X	38269.54	31858.09	34159.23	35987.31
RPS4Y1	11654.97	9212.129	11405.34	11241.47
RPS4Y2	0	0	0	0
RPS5	29251.45	24042.73	27841.98	28900.88
RPS6	52642.17	40861.35	49054.96	48741.98
RPS7	20866.56	18703.36	20192.89	20299.06
RPS8	48110.64	37518.94	40535.14	40599.79
RPS9	36634.69	30135.18	36171.28	38651.05
RPSA	38216.53	30359.5	38456.92	38219.38
RPUSD4	1182.667	1001.815	1172.424	1071.4
RRP12	4410.783	3803.205	1608.266	1242.981
RRP36	1614.622	1504.11	1254.524	1119.783
RRP7A	5181.935	5196.412	2986.956	2653.627
RRP8	845.1043	920.191	868.2178	785.7282
RRP9	1819.852	1679.735	863.3156	683.4864
RRS1	2748.327	2690.629	973.5285	751.015
RSL24D1	3497.619	3017.499	2403.875	2590.209
RUVBL1	2949.658	2908.663	2107.572	1756.219
RUVBL2	3934.244	4069.857	3263.107	2810.988
S100A1	107.9514	155.1141	186.1838	195.4852
SACS	2033.53	1618.284	950.632	801.82
SAMM50	2195.907	2044.109	3519.175	3427.208
SAR1A	5702.178	5451.896	5059.705	4929.302
SAR1B	2618.575	2818.561	2753.185	2569.26
SARS1	15807.4	15767.35	7358.94	8359.407
SARS2	603.6213	631.9855	901.6366	801.99
SBDS	5121.363	4585.706	3581.628	3520.146
SCG5	3185.12	3475.295	3801.51	4306.235
SCYL1	5775.42	6176.49	5493.292	5398.527
SDF2	1112.809	979.0562	1179.561	1208.029
SDF2L1	674.3592	778.9844	793.9352	611.6699
SEC11A	6081.275	5349.326	5373.975	5590.812

SEC11C	1079.342	1126.55	1219.71	924.3377
SEC13	3530.527	3657.911	3410.739	3131.055
SEC23A	4267.388	4246.445	4838.44	4258.869
SEC23B	3434.022	3493.969	3280.151	2981.241
SEC23IP	3278.032	3484.344	2684.649	2247.82
SEC24A	1653.344	1729.356	1745.696	1756.549
SEC24B	2281.08	2209.347	2499.52	2231.996
SEC24C	5220.429	5180.832	5880.275	5336.902
SEC24D	781.0165	703.615	901.5717	1042.665
SEC31A	7932.125	8092.998	8913.293	8217.328
SEC31B	782.1884	901.5862	1012.917	976.2542
SEC61A1	18486.76	18694.74	16110.64	15502.62
SEC61A2	467.2031	488.9743	363.6966	326.6951
SEC61B	1743.357	1749.433	1521.479	1453.477
SEC61G	875.8167	885.2327	728.7927	668.8786
SEC62	6953.353	6875.742	6523.134	5962.834
SEC63	5503.641	5180.417	4764.787	5020.389
SECISBP2	3328.519	3145.711	2176.136	2145.44
SEH1L	3696.751	2961.451	1607.628	1228.809
SEL1L	5498.197	5709.44	5349.675	4998.219
SEL1L2	0	0	0	0.106597
SELENOF	5581.84	5393.866	6637.675	6142.483
SELENOS	1988.824	1951.724	1245.732	1298.619
SEPSECS	393.1662	440.4152	433.8059	450.5262
SERP1	4329.61	4073.427	3470.692	3735.147
SERP2	392.8681	333.9031	478.2282	478.3825
SERPINA5	137.0393	119.0177	107.559	302.9635
SERPINH1	8292.827	7519.574	6603.187	6581.685
SERPINI1	74.65314	72.63335	98.05133	119.1858
SET	27635.14	25694.41	17174.31	16073.79
SFTPC	0.31716	0.151045	0	0
SGTA	5522.935	5734.222	4347.704	4167.915
SGTB	1367.428	1239.731	901.3249	903.0725
SIL1	1946.827	2045.905	3009.914	2868.429
SLIRP	677.917	624.6477	590.0246	520.9938
SNORD3A	1.089905	1.430665	0.345962	0.256117
SNU13	7607.357	7310.641	6527.598	5639.848
SNUPN	820.1278	782.9189	1244.847	1204.113
SPAG1	471.3233	471.1606	457.773	428.6834
SPARC	25835.73	32255.86	58784.83	54331.86
SPCS1	3126.259	3046.64	3630.464	3019.802
SPCS2	3050.699	2909.897	3328.834	3170.74
SPCS3	5146.236	5242.404	4890.974	4359.195
SPG7	3169.749	3132.921	3673.94	3223.982
SPTY2D1	1609.406	1676.996	1469.502	1378.022
SRP14	9233.428	9259.185	11791.64	12333.45
SRP19	1450.688	1545.876	1394.057	1316.734
SRP54	2352.958	2410.307	2087.815	1926.663

SRP68	3693.891	3388.933	3270.274	3049.487
SRP72	5973.349	5617.196	4798.582	4338.057
SRP9	5329.104	5455.985	5878.975	5170.266
SRPRA	8783.427	9468.616	8483.69	7657.987
SRPRB	3506.686	3490.921	2947.127	2631.728
SSR1	6068.279	5638.945	7120.66	6599.297
SSR2	6870.725	6348.205	7697.962	7853.643
SSR3	8149.785	7295.098	7021.166	6884.652
SSR4	5610.319	5981.998	7639.566	7562.478
SSRP1	6566.445	5959.619	4548.036	4378.201
ST13	14760.94	13052.19	14582.62	15077.63
STIP1	7978.36	6543.531	4909.562	4423.133
STT3A	6510.928	6517.747	7705.913	6384.376
STT3B	8444.7	7858.907	9863.444	9156.924
STUB1	4595.141	4521.72	4160.082	4320.859
SUGT1	1733.975	1490.687	1613.962	1483.583
SUPT16H	6517.344	6220.698	5211.278	4563.864
SUPT6H	10113.18	9886.624	7365.481	7305.634
SVIP	922.8256	970.4508	1111.982	986.5102
SYVN1	3286.226	3577.614	2547.414	2679.379
TARS1	8059.03	7988.752	3679.295	3852.39
TARS2	1272.661	1534.041	2116.052	1618.79
TARS3	537.3429	487.4407	452.8829	461.5631
TBCA	2265.539	2069.768	2162.102	2095.962
TBCB	2156.247	2314.793	2906.541	2520.645
TBCC	1027.958	1143.171	1017.276	995.9256
TBCD	4645.853	5116.32	6350.596	5615.326
TBCE	1185.017	1074.4	1154.537	1047.824
TBL3	3252.703	3736.973	2791.273	2350.745
TCF25	6248.775	6365.687	6035.96	5909.087
TCP1	8399.462	7265.599	5738.32	5497.609
TEX10	1663.172	1621.441	1117.935	914.8708
TFB1M	384.843	453.3515	451.0719	402.7825
TIMM10	697.2157	710.3255	437.554	414.6526
TIMM10B	3419.943	2851.47	2516.822	2288.166
TIMM13	4101.582	4035.894	3372.684	2937.397
TIMM17A	3328.315	3039.702	2609.616	2383.029
TIMM17B	1021.776	902.1294	1318.094	1224.946
TIMM21	1046.959	1001.83	1116.144	934.9543
TIMM22	1012.139	899.3564	1000.624	873.2635
TIMM23	2542.831	2559.041	1913.657	1762.072
TIMM23B	1072.968	943.3182	448.6917	406.133
TIMM29	623.9043	646.3599	667.3634	641.1313
TIMM44	2970.192	2879.764	1980.487	1910.559
TIMM50	3259.622	3564.22	2754.906	2259.56
TIMM8A	761.3604	729.844	373.9371	309.3402
TIMM8B	1290.108	1438.044	1120.553	1038.07
TIMM9	1125.911	826.1397	952.5981	974.8185

TMA16	782.7854	624.705	505.1951	422.5409
TMCO1	3102.61	3145.836	3645.715	3262.248
TMED3	4414.48	4386.844	5422.843	5022.64
TMED7	6953.693	7282.883	7084.183	6104.712
TMEM129	5333.645	5714.067	6769.239	6804.94
TMEM147	6116.338	5894.735	7635.141	6913.932
TMEM67	462.0298	476.2825	561.2974	553.0574
TMTC1	7989.46	8046.064	7539.433	7405.19
TMTC2	561.0418	644.933	579.3908	555.1298
TMTC3	979.9507	870.9454	966.5247	883.8851
TMTC4	1573.007	1565.139	1801.532	1559.942
TMUB1	1747.701	1771.16	2473.915	2528.61
TMX1	2421.867	2310.18	2803.838	2496.691
TMX2	5759.414	5772.378	5085.744	4682.871
TMX3	2478.623	2588.85	2317.78	1990.501
TMX4	3367.2	3624.29	4324.815	4138.768
TNPO1	9615.181	9438.735	7564.584	6645.048
TNPO2	5093.067	5108.975	4424.353	4580.467
TNPO3	2983.312	2660.856	2533.675	2500.434
TOMM20	15854.62	14497.47	14520.56	14407.51
TOMM20L	4.648834	4.951439	6.079432	0.697753
TOMM22	2198.061	2157.953	1993.614	1834.231
TOMM34	4084.424	3930.801	3689.676	3090.908
TOMM40	6677.843	7177.458	4005.955	3278.94
TOMM40L	678.6158	578.4461	1108.264	1024.835
TOMM5	1888.529	1682.893	1233.598	1045.767
TOMM6	2427.24	2250.066	2751.004	2201.518
TOMM7	1529.031	1198.153	1438.744	1570.287
TOMM70	4006.457	3898.189	3380.063	3001.165
TONSL	369.6072	372.0531	527.9455	487.9244
TPR	5446.722	5696.102	5004.545	4512.17
TRAM1	9618.231	9340.979	11069.31	9647.744
TRAM1L1	129.093	151.5636	324.752	255.5751
TRAM2	5960.788	6976.286	7936.304	7129.568
TRAP1	5352.403	5296.58	4709.194	4073.062
TRIB3	22011.53	23076.43	2486.449	3431.769
TRIM13	1395.892	1270.218	1192.198	1191.656
TRIM25	3482.813	4779.776	2494.165	2339.862
TRIP4	693.0218	735.198	593.3906	607.5506
TRMT10C	1135.615	1059.989	850.4901	678.4138
TRMT112	3548.67	3359.839	3363.111	2801.093
TRMT2B	818.5666	895.1922	1337.119	1305.623
TRMT61B	526.9082	612.3326	651.007	511.1563
TSC1	2623.93	2847.371	2972.979	2634.938
TSFM	1243.955	1391.707	1569.123	1244.135
TSR1	5733.345	5274.01	2919.76	2522.085
TSR2	1822.713	1907.633	2010.973	1861.75
TSR3	2869.996	2907.49	2246.752	2179.279

TTC1	2373.416	2598.893	2115.955	1957.72
TTC4	1157.003	942.4613	760.7847	635.8115
TTR	196.3203	281.0215	332.015	473.6286
TUFM	10587.79	10348.63	12063.68	10646.58
TUSC3	3595.998	3818.749	4994.542	4608.435
TXNDC11	3606.857	4166.463	5014.01	4420.969
TXNDC12	2341.821	2444.586	2145.899	2082.929
TXNDC16	587.8702	544.402	803.7956	959.1479
TXNDC5	11567.51	10976.91	11700.14	10461.28
TXNDC9	943.7805	871.0187	965.6818	866.0428
UBA5	1444.671	1636.09	1821.228	1853.467
UBA52	17040.8	15639.32	17018.81	16638.18
UBE2D1	1136.531	963.073	894.2672	786.5955
UBE2G1	2436.423	2176.973	2409.736	2383.112
UBE2G2	5146.937	5193.456	3384.876	3136.242
UBE2J1	4683.127	4375.449	4321.35	4590.612
UBE2J2	2661.399	2643.566	2086.815	2084.804
UBE2K	3493.357	3306.129	2909.309	2728.53
UBE4B	4602.798	4640.88	6563.521	6022.59
UBL4A	3086.652	2604.38	2934.084	2729.684
UBN1	3756.891	3178.962	3437.891	3420.875
UBQLN1	7188.177	6188.073	5547.774	5080.768
UBQLN2	2758.406	2680.727	3252.61	3111.892
UBQLN3	0	0	0	0
UBQLN4	4394.675	4067.192	3232.755	2819.981
UBQLNL	5.237076	7.729964	11.47671	18.47307
UBXN4	9982.355	8998.754	12771.81	12381.33
UBXN8	559.5759	541.32	512.9235	379.6082
UFC1	2200.071	1989.245	2582.765	2798.335
UFD1	1377.755	1438.39	1316.325	1187.2
UFL1	1628.245	1680.216	1739.424	1505.894
UFM1	3455.584	3534.793	2702.219	2694.148
UFSP1	292.6243	291.5836	138.1795	108.5999
UFSP2	1053.441	926.0214	1360.911	1381.136
UGGT1	3441.561	3122.853	3277.208	2745.989
UGGT2	1503.278	1466.262	1330.161	1194.163
UNC45A	3407.155	3475.149	5245.47	5017.631
UNC45B	0	0	0	0
UPF1	8739.939	8875.732	6030.791	5739.665
UPF2	1723.78	1539.36	1270.629	1240.459
UPF3A	2893.449	2832.314	2680.552	2502.384
UPF3B	727.2828	629.2332	505.5698	473.8235
URI1	2988.172	2767.13	2595.09	2478.627
USP10	4325.873	3996.346	3016.781	2542.638
USP14	3987.967	3172.827	2466.116	2119.023
USP19	4020.628	4427.896	4934.09	4617.738
USP21	1157.81	1042.495	1622.269	1601.951
USP30	680.5545	798.8772	1581.955	1676.528

UTP14A	1141.806	1027.748	791.332	698.2916
UTP15	1338.549	1245.757	635.1375	560.0145
UTP18	2439.234	2070.29	1730.709	1539.018
UTP4	3108.867	2815.462	1563.507	1447.301
UTP6	1947.783	1838.478	1303.5	1114.663
UXT	1128.183	1099.526	1252.23	1173.23
VARS1	4676.256	4642.542	4844.649	3447.105
VARS2	896.1369	906.6373	1360.317	1211.265
VBP1	1558.561	1429.417	1586.682	1363.168
VCP	19158.96	20552.96	19925.28	17552.16
VPS72	1402.798	1277.029	1449.357	1286.633
VTN	1001.662	1142.6	1350.572	1470.429
WARS1	15055.75	17600.61	4787.396	6384.841
WARS2	750.4038	788.3578	889.1927	900.6916
WDR12	2604.923	2563.518	1850.446	1545.402
WDR18	1648.277	1872.394	1641.309	1502.841
WDR3	4037.299	2997.807	1665.085	1367.405
WDR36	6798.266	5787.303	2942.964	2267.539
WDR43	4153.446	3653.116	1806.334	1521.482
WDR75	1594.797	1428.535	1086.746	962.0958
WDR83OS	3187.802	3214.013	3579.487	3530.166
XBP1	6214.21	6073.861	3157.027	3469.872
XPNPEP3	953.1234	927.4898	1037.071	894.4223
XPO1	3869.755	3192.454	6530.782	5621.57
XPO4	2519.385	2202.214	1908.148	1760.622
XPO5	4469.411	4121.262	2312.678	2021.313
XPO6	8512.796	8310.618	5499.435	5002.464
XPO7	3575.066	3418.338	4848.774	4485.405
XPOT	19056.08	17179.16	7461.913	8847.956
XRN2	4964.285	4596.208	5124.342	4662.634
YARS1	10335.95	10713.38	3116.78	3366.377
YARS2	823.0737	804.3703	699.5703	676.1148
YBEY	384.7759	324.6422	427.4172	419.9281
YME1L1	8674.334	8308.501	6267.325	6140.5
YOD1	1915.357	1990.177	1839.333	1386.573
YWHAE	17365.3	16680.79	17222.53	15612.1
YWHAZ	27710.35	21345.68	23355.14	22510.98
ZCCHC4	344.0206	372.8264	343.9463	372.334
ZFP42	72.40686	31.19585	16.83433	22.5042
ZMYND10	63.97616	53.13938	94.59695	94.42467
ZNF593	1332.638	1376.584	743.6813	656.8906
ZNF598	3304.629	3115.804	1849.252	1659.769

Supplementary Table S8: Autophagy-lysosomal pathway genes showing significance within timepoint and between control vs. POS exposure

Adjusted p<0.05	Adjusted p<0.1	p<0.01	p<0.05	p<0.1
IGF1	*ABHD5*	*ABHD5*	*ABHD5*	*ABHD5*
MAP2K1	*IGF1*	*ACP3*	*ACP3*	*ACBD5*
PLD1	*MAP2K1*	*ADRB2*	*ADRB2*	*ACP3*
RILP	*PLD1*	*ATG16L1*	*ATG10*	*ADRB2*
STBD1	*PPARGC1A*	*ATP6V0E2*	*ATG16L1*	*AGA*
	RILP	*CASTOR1*	*ATG2B*	*ARSG*
	STBD1	*DRAM1*	*ATG4C*	*ATF5*
		FBXW7	*ATG4D*	*ATF6*
		GJA1	*ATL3*	*ATG10*
		GPLD1	*ATP13A2*	*ATG16L1*
		GRAMD1A	*ATP5IF1*	*ATG16L2*
		IGF1	*ATP6V0A2*	*ATG2B*
		IL17RB	*ATP6V0E2*	*ATG4C*
		KIF5B	*ATP6V1G2*	*ATG4D*
		KLHL22	*ATP6V1G3*	*ATL3*
		LAMTOR2	*BCL2L13*	*ATP13A2*
		MAP2K1	*BMF*	*ATP5IF1*
		NFKB2	*BSG*	*ATP6AP1*
		NFKBIA	*CASTOR1*	*ATP6AP2*
		NLRX1	*CCZ1*	*ATP6V0A2*
		PHLPP1	*CEBPB*	*ATP6V0B*
		PLA2G15	*CHMP4C*	*ATP6V0E2*
		PLD1	*CHMP6*	*ATP6V1G2*
		PPARGC1A	*CSNK1D*	*ATP6V1G3*
		PRKAG2	*CSNK2A2*	*BAG3*
		QSOX1	*CTSH*	*BCL2L13*
		RAB17	*CTSK*	*BCLAF1*
		RAB23	*CUL3*	*BLOC1S1*
		RELB	*DAP*	*BMF*
		RHEB	*DAPK1*	*BORCS6*
		RICTOR	*DAPK3*	*BRSK2*
		RILP	*DEPTOR*	*BSG*
		RUBCNL	*DNASE2*	*CAB39*
		SAMM50	*DRAM1*	*CAMKK2*
		SH3BP4	*EEF1A2*	*CASTOR1*
		STBD1	*EIF2S1*	*CCDC88A*
		STRADA	*EVA1A*	*CCPG1*
		TNFAIP3	*FBXW7*	*CCZ1*
		TSPO	*FIG4*	*CDK5*
		USP14	*FKBP8*	*CDKN1B*
			FOSB	*CEBPB*
			FOSL1	*CHMP3*
			FOXO3	*CHMP4C*

GFAP	*CHMP5*
GJA1	*CHMP6*
GLB1	*CHUK*
GLI1	*CSNK1D*
GPLD1	*CSNK2A2*
GPSM1	*CTSB*
GRAMD1A	*CTSH*
GRN	*CTSK*
HK2	*CTSS*
HS1BP3	*CUL1*
HSP90AB1	*CUL3*
HSPB8	*CUL4A*
HYAL2	*DAGLB*
IGF1	*DAP*
IL10RA	*DAPK1*
IL17RB	*DAPK2*
INPP5E	*DAPK3*
KDR	*DAXX*
KIF5B	*DCAF12*
KLHL22	*DDX5*
KRAS	*DELE1*
LAMP1	*DEPDC5*
LAMTOR2	*DEPTOR*
LITAF	*DNASE2*
MAP1LC3B	*DRAM1*
MAP2K1	*DRD2*
MAP3K7	*EEF1A2*
MAPK15	*EHMT2*
MCOLN3	*EIF2S1*
MON1B	*ERN1*
MTMR8	*EVA1A*
MTMR9	*FBXW5*
NEDD4L	*FBXW7*
NFKB2	*FIG4*
NFKBIA	*FKBP8*
NLRX1	*FOSB*
NOX4	*FOSL1*
NPRL2	*FOSL2*
NRAS	*FOXO3*
NRBF2	*FUNDC1*
NUFIP1	*FYCO1*
PAQR3	*GAA*
PDCD6IP	*GABARAP*
PHLPP1	*GFAP*
PICALM	*GJA1*
PIK3CD	*GLB1*
PLA2G15	*GLI1*
PLA2G5	*GNAI3*

PLD1	*GPLD1*
PLD3	*GPSM1*
PPARGC1A	*GRAMD1A*
PPP1R13L	*GRN*
PPP2R2A	*HGSNAT*
PRKAG2	*HIF1A*
PRKCQ	*HK2*
PSME1	*HMOX1*
PTPN2	*HPSE*
PYGB	*HS1BP3*
QSOX1	*HSP90AB1*
RAB17	*HSPB8*
RAB23	*HYAL2*
RAB29	*IFT88*
RAB5A	*IGF1*
RABGEF1	*IGFBP3*
REL	*IL10RA*
RELA	*IL17RB*
RELB	*INPP5E*
RHEB	*ITPR1*
RICTOR	*JMY*
RILP	*KDR*
RIPK1	*KIF1B*
RNF185	*KIF5B*
RUBCN	*KLHL22*
RUBCNL	*KRAS*
RUFY3	*KXD1*
SAMM50	*LAMP1*
SEH1L	*LAMTOR2*
SH3BP4	*LEPR*
SKP2	*LIPA*
SMURF1	*LITAF*
SNX7	*MAN2B1*
SPP1	*MAN2B2*
STBD1	*MAP1LC3B*
STRADA	*MAP1LC3C*
STX17	*MAP2K1*
TBC1D14	*MAP2K2*
TEX264	*MAP3K7*
TFEB	*MAPK15*
TICAM1	*MAPK8*
TMEM175	*MAPKAPK2*
TMEM41B	*MCL1*
TNFAIP3	*MCOLN3*
TOM1	*MFN1*
TP53INP2	*MLST8*
TSC1	*MON1B*
TSPO	*MTMR14*

		UBA5	*MTMR8*
		UFM1	*MTMR9*
		USP14	*MYO6*
		USP35	*NEDD4L*
		VAMP2	*NEU1*
		VPS33A	*NFKB2*
		VPS4B	*NFKBIA*
		WASHC1	*NIPSNAP1*
		WDR24	*NLRX1*
		WIPI2	*NOX4*
		YAP1	*NPRL2*
		ZKSCAN3	*NPRL3*
			NRAS
			NRBF2
			NUFIP1
			OGT
			PAQR3
			PDCD6IP
			PEA15
			PHB2
			PHLPP1
			PICALM
			PIK3C3
			PIK3CA
			PIK3CD
			PIK3R2
			PIKFYVE
			PIP5K1B
			PLA2G15
			PLA2G5
			PLD1
			PLD3
			PML
			PPARGC1A
			PPP1R13L
			PPP2R2A
			PRKACA
			PRKAG2
			PRKAG3
			PRKCQ
			PRKD1
			PRTN3
			PSME1
			PTK2
			PTPN2
			PYGB
			QSOX1
			RAB17

RAB23
RAB26
RAB29
RAB5A
RAB8B
RABGEF1
RAF1
RARA
REL
RELA
RELB
RHEB
RICTOR
RILP
RIPK1
RMC1
RNF152
RNF185
RUBCN
RUBCNL
RUFY3
SAMM50
SEC23A
SEC23B
SEC23IP
SEC24A
SEC24D
SEC31B
SEH1L
SESN3
SH3BP4
SIRT2
SKP2
SLC25A4
SMPD1
SMURF1
SNCAIP
SNX7
SPP1
SRC
SREBF2
STBD1
STK3
STK4
STRADA
STX17
STX6
STX7

	STX8
	SUPT20H
	SYT11
	TAB3
	TBC1D14
	TBC1D2
	TBK1
	TEX264
	TFEB
	TICAM1
	TLR4
	TMEM150B
	TMEM175
	TMEM41B
	TMEM59
	TNFAIP3
	TOM1
	TOMM7
	TP53INP2
	TPCN2
	TRAPPC1
	TRIM17
	TSC1
	TSPO
	TUFM
	TXNDC5
	UBA5
	UBXN6
	UFM1
	USP14
	USP15
	USP35
	VAMP2
	VMP1
	VPS13D
	VPS33A
	VPS4B
	WASHC1
	WDR24
	WDR45B
	WDR59
	WDR91
	WIPI1
	WIPI2
	YAP1
	YWHAG
	ZFYVE26
	ZKSCAN3

Timepoint post POS exposure (hours)	Upregulated	Downregulated
4	9 GO terms: Protein binding, GTPase activity, GTP binding, GDP binding, protein-containing complex binding, protein-membrane adaptor activity, G-protein activity, protein kinase binding, ubiquitin-like protein transferase activity.	3 GO terms: Protein binding, protein dimerization activity, cysteine-type endopeptidase activity.
6	13 GO terms: Protein binding, identical protein binding, GDP binding, protein-containing complex binding, GTPase activity, GTP binding, ATP binding, protein-membrane adaptor activity, G-protein activity, protein serine kinase activity, protein serine/threonine kinase activity, ubiquitin-like protein transferase activity, GTPase binding.	3 GO terms: Cysteine-type endopeptidase activity, protein-phosphatidylethanolamide deconjugating activity, cysteine-type peptidase activity.
24	8 GO terms: Protein binding, cysteine-type endopeptidase activity, protein-phosphatidylethanolamide deconjugating activity, cysteine-type peptidase activity, protein serine/threonine kinase activator activity, GDP binding, protein-macromolecule adaptor activity, protein dimerization activity.	21 GO terms: Protein binding, Rho-dependent protein serine/threonine kinase activity, eukaryotic translation initiation factor 2alpha kinase activity, ribosomal protein S6 kinase activity, various histone kinase activity (14 sub-types), 3-phosphoinositide-dependent protein kinase activity, DNA-dependent protein kinase activity, AMP-activated protein kinase activity.
48	4 GO terms: Protein binding, protein dimerization activity, GTPase binding, cysteine-type endopeptidase activity.	2 GO terms: Protein binding, identical protein binding.

Supplementary Table S9: All upregulated and downregulated differentially expressed genes (DEGs) for each timepoint from Figure 6 were inputted into the UniProt ID mapping tool (https://www.uniprot.org/id-mapping). Gene ontology (GO) networks were subsequently identified mapping to their respective molecular functions using the Database for Annotation, Visualization, and Integrated Discovery (DAVID) bioinformatics tool (https://davidbioinformatics.nih.gov/). Genes are listed in the order following the most coverage to the least.
